# Effect of Remote Amine Groups on Ground- and Excited-State Properties of Terpyridyl d-Metal Complexes

**DOI:** 10.3390/molecules30112386

**Published:** 2025-05-29

**Authors:** Anna Kryczka, Joanna Palion-Gazda, Katarzyna Choroba, Barbara Machura

**Affiliations:** Institute of Chemistry, University of Silesia, 9 Szkolna Str., 40-006 Katowice, Poland; anna.kryczka@us.edu.pl (A.K.); katarzyna.choroba@us.edu.pl (K.C.)

**Keywords:** amine-functionalized terpyridines, d-metal complexes, photophysics, electrochemistry, intraligand charge transfer transitions

## Abstract

Over the last nine decades, 2,2′:6′,2″-terpyridine (terpy) derivatives and their transition d-metal complexes have been extensively explored due to their unique and widely tuned optical, electrochemical, and biological properties. Terpyridyl transition metal complexes occupy a prominent position among functional molecular materials for applications in optoelectronics, life science, catalysis, and photocatalysis, as well as they have played a key role in determining structure–property relationships. This review summarizes the developments of amine-functionalized R-C_6_H_4_-terpy systems and their d-metal complexes, largely concentrating on their photophysical and electrochemical properties. Functionalization of the terpy core with the electron-rich group, attached to the central pyridine ring of the terpy backbone via the phenylene linker, gives rise to organic push–pull systems showing the photoinduced charge flow process from the peripheral donor substituent to the terpy acceptor. The introduction of amine-functionalized R-C_6_H_4_-terpy systems into the coordination sphere of a d-metal ion offers an additional way for controlling the photophysics of these systems, in agreement with the formation of the excited state of intraligand charge transfer (ILCT) nature. Within this review, a detailed discussion has been presented for R-C_6_H_4_-terpys modified with acyclic and cyclic amine groups and their Cr(III), Mn(I), Re(I), Fe(II), Ru(II), Os(II), Pt(II), and Zn(II) coordination compounds.

## 1. Introduction

2,2′:6′,2″-Terpyridine derivatives constitute a class of multi-N-donor building blocks with excellent complexing abilities towards the vast majority of metal ions, extensively explored in coordination and supramolecular chemistry for over nine decades [1,2,3,4,5,6,7,8,9,10,11,12,13,14,15,16,17,18,19,20,21,22]. Their d-metal complexes occupy a prominent position among functional molecular materials appealing for employment in optoelectronics, life science, catalysis, and photocatalysis [7,10,18,22,23,24,25,26,27,28,29,30,31,32,33,34,35]. Owing to a wide range of possible structural modifications of the terpy backbone by the efficient Kröhnke condensation [36,37], terpyridyl metal complexes have also played a key role in determining structure–properties relationships, crucial in a view of their potential applications and making further progress in obtaining materials with optimal set of functional parameters. Depending on structural modifications of the terpy framework, ancillary ligands and metal ions, these systems may offer different excited states to control their photophysical parameters, including the most common metal-to-ligand charge transfer (MLCT) and other ones: ligand-to-ligand charge transfer (LLCT), ligand-centered (IL), metal-centered (MC), or their superposition. Photophysics of terpyridyl transition metal compounds become even more complex when the terpy backbone is modified with electron-donating moieties [38,39,40,41].

Functionalization of the terpy core with an electron-rich group, attached to the central pyridine ring of the terpy backbone via the phenylene linker, gives rise to organic push–pull systems (D–π–A) with the photoinduced charge flow process from the peripheral donor substituent to the terpy core. The efficiency of the intramolecular charge transfer in donor–π–acceptor terpy-based compounds is controlled by the electron-donating component, electronic coupling between donor and acceptor moieties, as well as the environment–solvent polarity, and hydrogen bonding with polar solvents [42,43,44]. The introduction of R-C_6_H_4_-terpy with a strong electron-donating group (R) into a coordination sphere of d-metal ions significantly enhances the electron-accepting properties of the terpy backbone and offers an additional way to control of photophysics of resulting coordination compounds owing to the involvement of intraligand charge transfer (ILCT) transitions. Also, the hyperpolarizability of these systems may increase upon R-C_6_H_4_-terpy coordination to metal ions, making it possible to obtain materials with nonlinear optical (NLO) responses for employment in optoelectronics and photonics [45,46,47,48].

Last but not least, terpy-based ligands provide the possibility to modify the photophysical properties of resulting metal complexes through different coordination modes, especially a bidentate terpy-κ^2^N and tridentate terpy-κ^3^N, as clearly demonstrated for Re(I) compounds [38].

In this review, we summarize the developments concerning R-C_6_H_4_-terpys functionalized with acyclic and cyclic amines, and their d-metal complexes, largely concentrating on their electrochemical and photophysical features in view of structure–property relationships and potential applications. In our discussion, the findings for Cr(III), Mn(I), Re(I), Fe(II), Ru(II), Os(II), Pt(II), and Zn(II) complexes with amine-functionalized R-C_6_H_4_-terpy ligands were taken into consideration. According to the literature survey, the d-metal complexes given above are the most recognizable and most extensively investigated in view of their ground and excited-state properties.

## 2. 2,2′:6′,2″-Terpyridines Modified with Acyclic and Cyclic Amines

**Scheme 1:**
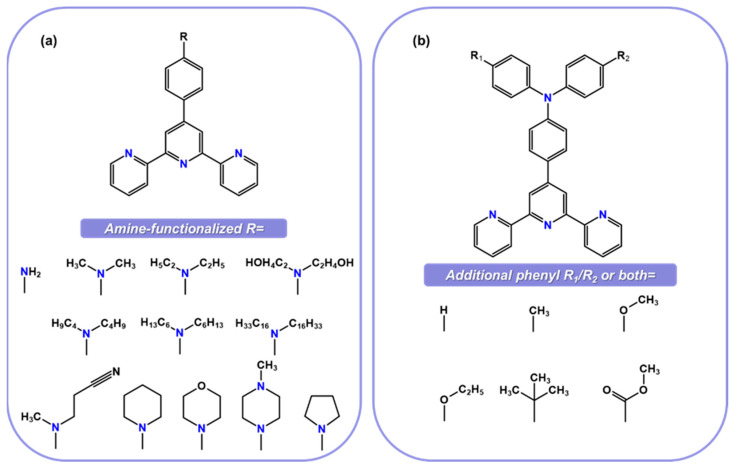
Molecular structures of amine-functionalized R-C_6_H_4_-terpy ligands (**a**) and Ph_2_N-C_6_H_4_-terpy with additional substituents introduced into the phenyl rings of the amine component (**b**).

R-C_6_H_4_-terpys with acyclic and cyclic amines attached to the central pyridine ring of the π-accepting component (terpy) via the phenylene linker (Scheme 1) are typical examples of push–pull systems of the D–π–A type. According to the two-form model developed by Barzoukas and Blanchard-Desce [49], the electronic properties of such systems are determined by the superposition of the limiting electronic states: a neutral D–π–A and a charge transfer one D^+^–π–A^−^, with the degree of mixing strongly affected by polarity, viscosity, and pH of the environment.

X-ray studies of amine-functionalized R-C_6_H_4_-terpys confirm a high degree of π-delocalization of the amine donor with π-accepting terpy unit [50,51]. The distances C–N_Ph-N(amine)_ are noticeably shorter than the single C–N bond length (1.47 Å), and the dihedral angle between the phenyl and terpy ring falls in the range 4.20–44.78° (Table S1).

**Figure 1:**
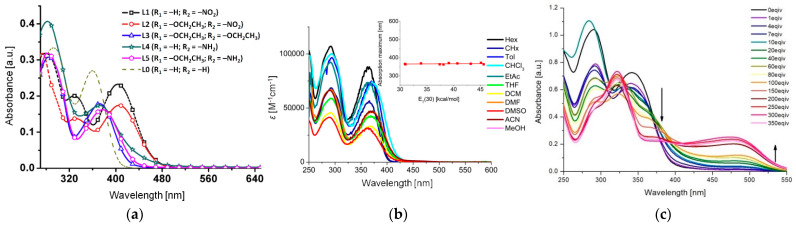
UV-Vis spectra of Ph_2_N-C_6_H_4_-terpy with additional substituents introduced into the phenyl rings of the amine component in benzene solution (**a**), 4′-(4-(di(4-tert-butylphenyl)amine)phenyl)-2,2′,6′,2″-terpyridine in solvents of different polarities (**b**), and variations in the absorption spectra of Me_2_N-C_6_H_4_-terpy in CHCl_3_ upon addition of trifluoroacetic acid (TFA) (**c**). The spectra are readapted from the works [62], [67], and [66], respectively.

The most extensive studies of amine-functionalized R-C_6_H_4_-terpys were performed by the Authors of reports [52,53,54,55,56,57,58,59,60,61,62,63,64,65,66,67,68,69] (Tables S2 and S3). The lowest energy absorption band of these systems, absent in separate donor and acceptor components [67], was assigned experimentally and theoretically to the intramolecular charge transfer transition (ICT) [52,53,54,55,56,57,58,59,60,61,62,63,64,65,66,67,68,69]. As demonstrated in Table S2 and Figure 1, its energy position is significantly affected by the electron-donating ability of the remote amine group, contrary to the environment polarity, which was evidenced to have a rather negligible impact on the ICT absorption maximum. As the amine substituent was found to raise the HOMO (Highest Occupied Molecular Orbital) energy level, the lowest energy absorption of R-C_6_H_4_-terpys occurs at longer wavelengths compared to Ph-terpy. High intensity of the ICT absorption of amine-functionalized R-C_6_H_4_-terpys was rationalized by a large overlap between the HOMO and LUMO (Lowest Unoccupied Molecular Orbital), involved in S_0_→S_1_ transition assigned to the ICT absorption. Although HOMO and LUMO are generally localized on different molecule parts (the amine for the HOMO and terpy for the LUMO), both are noticeably contributed by phenylene orbitals, which results in an increased value of the S_0_→S_1_ transition moment [56,58,65,67]. The dominated contribution of the amine in the HOMO and terpy in the LUMO was also evidenced by electrochemical studies, which confirmed that the oxidation process in these systems is associated with oxidation of the electron-rich group, and the reduction process occurs within the terpy moiety [57,65,66].

Acidification of solutions of amine-functionalized R-C_6_H_4_-terpys (Figure 1c) results in a remarkable intensity decrease of the lowest energy absorption and appearance of a new and substantially red-shifted band, in agreement with the enhanced electron-withdrawing ability of terpy upon protonation [53,67]. An even greater shift towards longer wavelengths in the ICT absorption is evoked by the coordination of the amine-functionalized R-C_6_H_4_-terpy to metal ions [53,60,61,64].

**Figure 2:**
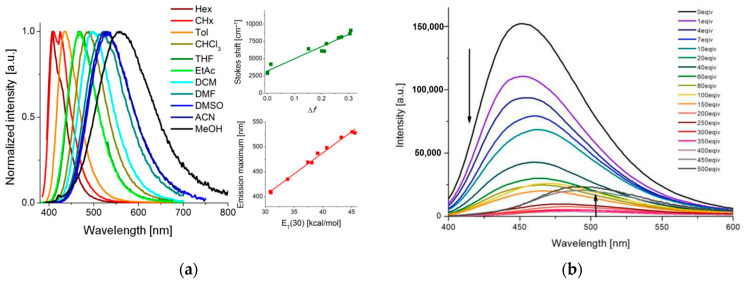
Fluorescence spectra of 4′-(4-(di(4-tert-butylphenyl)amine)phenyl)-2,2′,6′,2″-terpyridine in solvents of different polarities, along with Lippert–Mataga plots (**a**) and variations in the fluorescence spectra of Me_2_N-C_6_H_4_-terpy in CHCl_3_ upon addition of trifluoroacetic acid (TFA) (**b**). The spectra are readapted from works [67] and [66], respectively.

Contrary to the absorption behavior, the emission of amine-modified R-C_6_H_4_-terpys was found to show very strong positive solvatochromism (Figure 2a), indicating large stabilization of the excited state in more polar solvents. The red-shift of the emission with an increased polarity is accompanied by changes in the spectral profile of the band, from structured in non-polar solvents to structureless and very broad band in the polar environment, typically of push–pull systems, where the locally excited (LE), dominated in the non-polar environment, is switched to ICT in polar solvents. In solvents of medium polarity, these systems may show dual emission behavior attributed to the occurrence of both LE to ICT emissions. Also, the fluorescence quantum yield (Φ) of R-C_6_H_4_-terpys was demonstrated to be greatly affected by the solvent polarity, generally showing a marked decrease with increasing solvent polarity due to the acceleration of ICT→TICT transfer (TICT—twisted intramolecular charge transfer). A dramatic reduction in Φ in methanol or ethanol solutions was assigned to interactions between alcohol and R-C_6_H_4_-terpy molecules through hydrogen bonds [52,53,54,55,56,57,58,59,60,61,62,63,64,65,66,67,68,69]. Except for R-C_6_H_4_-terpys with triphenylamine-based moieties, which were found to be strongly emissive, amine-modified R-C_6_H_4_-terpys generally exhibit moderate or weak emission (Table S2).

The effect of protonation on the emission of amine-modified R-C_6_H_4_-terpys was reported in [53,54,66,67]. The formation of the protonated form was manifested in a progressive decrease in the emission intensity of the non-protonated R-C_6_H_4_-terpy and the appearance of a new emission band at longer wavelengths (Figure 2b).

With the use of femtosecond fluorescence up-conversion (FlUC) and femtosecond transient absorption (TA) spectroscopy, the presence of three ICT conformers was evidenced for 4′-(4-(di(4-tert-butylphenyl)amine)phenyl)-2,2′,6′,2″-terpyridine. The photoexcited state ICT_1_ was demonstrated to undergo a transfer into two conformers, ICT_1_′ and ICT_2_, as a result of structural changes in dihedral angles between the terpy, phenylene, and amine units. While ICT_1_′ was evidenced to decay to the ground state through a fluorescence channel, ICT_2_ was a dark state. The ultrafast photo-response of 4′-(4-(di(4-tert-butylphenyl)amine)phenyl)-2,2′,6′,2″-terpyridine was investigated as a function of the solvent polarity and viscosity. It was found that the transfer process ICT_1_→ICT_1_′ is slowed down by the solvent viscosity, while the energy barrier of the ICT_1_→ICT_1_′ process is reduced by the solvent polarity, leading to its acceleration in polar solvents [69].

Amine-modified R-C_6_H_4_-terpys, yielding large static quadratic hyperpolarizabilities due to large dipole changes, were also reported to show a remarkable two-photon activity (TPA). Some of these compounds were successfully utilized for cell bioimaging under two-photon confocal microscopy [55,58,59,60,62]. According to the two-form model developed by Barzoukas and Blanchard-Desce [49], the TPA cross-section (δ_TPA_), which determines NLO properties, is a function of the MIX parameter, specifying the degree of mixing of the limiting D–π–A and D^+^–π–A^−^ states. The values of the TPA cross-section of amine-modified R-C_6_H_4_-terpys fall in the range 87–1060 GM (1 GM = 10^−50^ cm^4^ s photon^−1^), confirming a profound impact of the amine electron-donating ability (Table S4). By analogy to the one-photon excited fluorescence behavior, two-photon excited fluorescence spectra of amine-modified R-C_6_H_4_-terpys obtained under NIR photoexcitation show positive solvatochromism (Table S4). The bathochromic shift between two-photon and one-photon emission bands in the same solvent was rationalized by the reabsorption effects, being non-negligible in the case of two-photon excited spectra recorded in concentrated solutions [58,60,62]. The impact of electron-withdrawing and electron-donating substituents attached to the triphenylamine unit of Ph_2_N-C_6_H_4_-terpy on the nonlinear optical properties of resulting D–π–A chromophores was discussed in refs. [58,59,62]. It was demonstrated that additional electron-rich groups resulted in an enhanced TPA activity, contrary to electron-withdrawing substituents, which were found to reduce TPA cross-sections. Also, the coordination of R-C_6_H_4_-terpys to metal ions may induce an increase in TPA response [60]. Regarding that two-photon confocal microscopy is a superior alternative to one-photon one, providing a deeper tissue penetration, lower tissue autofluorescence, suppressed background signals, and reduced phototoxicity, the development of new push-pull molecules with improved nonlinear properties is strongly desirable and remains one of the most important tasks of modern chemistry.

## 3. Zn(II) Complexes

**Scheme 2:**
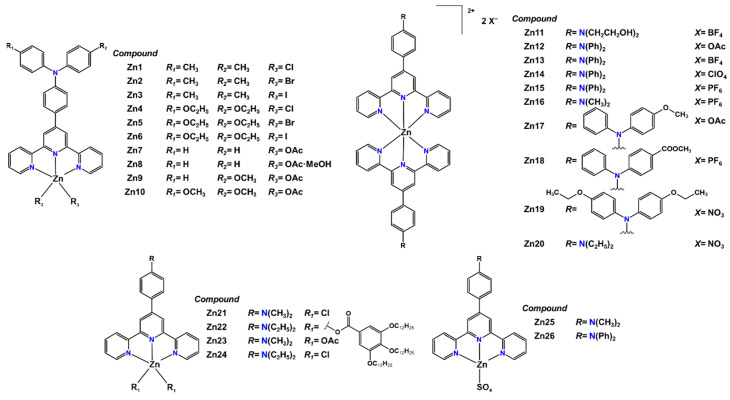
Zinc(II) complexes with amine-modified R-C_6_H_4_-terpy ligands.

The photophysical and relevant electrochemical data of Zn(II) complexes with amine-substituted R-C_6_H_4_-terpy ligands (Scheme 2) are gathered in Tables S5–S7 [60,64,70,71,72,73,74,75,76,77,78,79,80,81,82,83]. Some of these systems were designed and comprehensively investigated as biosensors and efficient two-photon probes for bioimaging. Zn(II) is the second most abundant d-metal ion in the human body, and its compounds exhibit excellent biocompatibility. The introduction of push–pull terpy-based ligands into the Zn(II) coordination sphere results in the strong intraligand charge transfer (ILCT), which is necessary for NLO response. By analogy to free ligands, the HOMO of these systems is mainly localized on the amine moiety, while LUMO is contributed by π* orbitals of the terpy framework. Another great advantages of zinc-based terpyridine complexes are their low cost and convenient and efficient synthesis through the reaction of Zn(II) salts with appropriate R-C_6_H_4_-terpy ligands.

The maximum of the longest wavelength absorption, predominantly originating from ILCT transitions, falls in the range 325–431 nm for mono-terpyridyl Zn(II) complexes and 407–458 nm for bis-terpyridyl ones. In agreement with the ILCT mechanism, the low-energy absorptions of [ZnX_2_(R-C_6_H_4_-terpy)] and [Zn(R-C_6_H_4_-terpy)_2_]X_2_ are strongly solvent-dependent and bathochromically shifted relative to those of free ligands (Tables S2 and S3). Upon coordination of R-C_6_H_4_-terpy to the Zn(II) ion, the ionic charge of the metal center makes the terpy backbone a stronger π-acceptor group, reducing the HOMO–LUMO gap of Zn(II) systems by the LUMO stabilization.

To some extent, the lowest energy absorption of **Zn1**–**Zn6** was also found to be contributed by ligand-to-ligand charge transfer (LLCT) transitions [77,78]. A regular bathochromic shift in the order **Zn1** < **Zn2** < **Zn3** was explained by the increased polarizability of halogen ions, resulting in the energy lowering of the whole molecule [77].

The concentration-dependent UV-Vis spectra of **Zn7** in DMSO revealed the dissociation/association equilibrium [Zn(Ph_2_N-C_6_H_4_-terpy)(OAc)_2_] ↔ [Ph_2_N-C_6_H_4_-terpy+Zn(OAc)_2_], manifested by the presence of a clear isosbestic point and overlapping the spectra of **Zn7** recorded at the lower concentration with that of the free ligand. Relative to the second-row transition metal ions, Zn(II) ions form weaker coordination bonds with the chelating terpy ligand, making possible the reversible dissociation/association equilibrium [64].

The absorption properties of **Zn21**, designed as a sensor, were additionally investigated in the presence of numerous anions. It was demonstrated that an addition of pyrophosphate (PPi) anions and nucleotide triphosphates (ATP, GTP and CTP) induced a noticeable bathochromic shift (~30 nm) and decrease in the absorption intensity, contrary to anions F^−^, Br^−^, I^−^, SO_4_^2−^, AcO^−^, NO_3_^−^, CO_3_^2−^, HPO_4_^2−^, and PO_4_^3−^, whose presence resulted in negligible changes in the spectra profile of **Zn21 [71]**.

In agreement with the closed-shell electronic configuration of Zn(II) ion (d^10^), the emissive excited state of [ZnX_2_(R-C_6_H_4_-terpy)] and [Zn(R-C_6_H_4_-terpy)_2_]X_2_ complexes is of ^1^ILCT character. Their broad and structureless emission band is affected by the electron-donating ability of the appended amine group, co-planarity between the donor and acceptor moieties of R-C_6_H_4_-terpy, and the environment polarity [61,84]. The positive solvatochromism, typical of the ILCT emission, was demonstrated for **Zn11** [60]. As reported in works [77,78,80], the luminescent properties of these systems are also affected by the nature of X ligands. The decrease in the quantum yield of **Zn1** > **Zn2** > **Zn3** was correlated with the impact of the halide ions on the molecular planarity of the Zn(II) complexes. The larger the molecular planarity was, the enhanced the fluorescence quantum yield was observed [77]. The difference in the emission color of [Zn(Ph_2_N-C_6_H_4_-terpy)_2_]X_2_ (X = CH_3_COO^−^, BF_4_^−^, ClO_4_^−^, PF_6_^−^) (**Zn12**–**Zn16**), from green-yellow to orange-red (from 549 to 622 nm), in CH_2_Cl_2_ solution was assigned to variations in the electrostatic interaction between Zn(II) ion and counterions. These systems were evidenced to possess two competitive ILCT excited states with energies controlled by the electrostatic interaction between counterions and Zn(II) ions. The controllable excited-state properties of [Zn(Ph_2_N-C_6_H_4_-terpy)_2_]X_2_ make these complexes very appealing for optoelectronic applications. In particular, by doping of 0.6 wt% [Zn(Ph_2_N-C_6_H_4_-terpy)_2_](PF_6_)_2_ into poly(ethyleneglycol)-block-poly(propyleneglycol)-block-poly(ethyleneglycol) (PEG–PPG–PEG), the white emission was achieved [80].

The solid-state luminescence of **Zn7** (λ_em_ = 555 nm, τ = 2.2 ns, Φ = 0.13) and **Zn8** (λ_em_ = 520 nm, τ = 3.3 ns, Φ = 0.43) was found to be impacted by the crystal solvent, which induces variations in the molecular conformation and crystal packing. The striking difference in their molecular structure was the dihedral angle between the central pyridine and phenylene rings, equal to 4.34° for **Zn7** and 33.86° for **Zn8**. Expectedly, the reduction in the dihedral angle, resulting in a better communication between the amine and terpy units and larger π-conjugation, leads to the red-shifted photoluminescence of the solvate-free form. The microcrystalline solid of **Zn7** was further demonstrated to exhibit vapoluminochromism. Its emission color was altered reversibly by the permeation of solvent vapors (toluene, dichloromethane, and acetone). In turn, the mechanochromism was found for the compound **Zn8** [76].

The luminescence studies of **Zn7** in solution revealed the dissociation/association equilibrium [Zn(Ph_2_N-C_6_H_4_-terpy)(OAc)_2_] ↔ Ph_2_N-C_6_H_4_-terpy + Zn(OAc)_2_], as mentioned above, supported also by the concentration-dependent UV-Vis spectra. Due to the reversible ligand dissociation, the complex **Zn7** features multi-functional luminochromism. It shows solvatoluminochromism covering the whole visible range, and it can be used to identify the mixing ratio of two solvents. Concerning that the couple [Zn(Ph_2_N-C_6_H_4_-terpy)(OAc)_2_]/Ph_2_N-C_6_H_4_-terpy undergoes reversible thermoluminochromism, it can also be utilized as a molecular thermometer. Last but not least, the complex **Zn7** exhibits a distinctive acid response [64].

Outstanding emissive photophysical properties were revealed for the room-temperature metallomesogen **Zn22**. The liquid crystallinity of this system was induced by two gallate monocoordinating ligands (Scheme 2). The quantum yield of its ILCT fluorescence (λ_em_ = 488 nm) in solution reaches the value of 95%. In the mesophase (542 nm), it drops to Φ = 20.2% but still stays at a high level. Both quantum yield values of **Zn22** are superior to those confirmed for the analogous system with a methoxy group instead of –N(C_2_H_5_)_2_ (6.5% in solution and 4% in the mesophase). The polarized emission of the oriented film of **Zn22** exhibits a dichroic emission ratio (I_|_/I_⊥_) of 1.30 [75].

The excited properties of zinc-based terpyridine complexes were also extensively explored in view of their potential applications. The formed nanoaggregates of **Zn21** in the presence of pyrophosphate (PPi) were found to emit very strong fluorescence (with 500-fold enhancement), which allowed for to selective detection of PPi at nanomolar concentrations, also among other ATP, ADP, GTP, and CTP nucleotide phosphates. Due to the excellent sensitivity towards PPi, the Zn(II) complex was successfully employed to stain and record confocal fluorescence microscopy images of HeLa cells [71]. Aggregation-induced emission (AIE) materials based on the water-soluble terpyridine zinc complexes **Zn7**, **Zn9**, **Zn10**, and **Zn23** were demonstrated to possess great potential in the visualization of latent fingerprints under UV light or ambient sunlight. By modifying the appended amine group, the image color was tuned from green to orange. Important advantages of these systems are extremely low detection limitation (<20 × 10^−6^ M) and very short incubation time in water (<3 s), in accompany with high contrast and ease of development on various surfaces [70].

The complexes **Zn25** and **Zn26** were evaluated as photocatalysts for the degradation of methylene blue. This widely used and highly toxic heterocyclic aromatic cationic dye easily accumulates in the environment, and its removal is one of the most challenging tasks. It was the first report concerning the photocatalytic activity of the terpyridyl metal complexes against the degradation of methylene blue, confirming that terpyridyl complexes have great potential to be efficient, eco-friendly photocatalysts. Relative to [Fe(Me_2_N-C_6_H_4_-terpy)_2_](PF_6_)_2_ (**Fe3**) and [Fe(Ph_2_N-C_6_H_4_-terpy)_2_](PF_6_)_2_ (**Fe2**) (see Section 4), zinc-based terpyridine complexes exhibited higher photocatalytic degradation efficiencies, 61.56% for **Zn25** and 65.91% for **Zn26**, which was rationalized by the presence of vacant sites in Zn(II) systems, allowing their better interactions with dye molecules [73]. The authors of the report [72] developed a fluorometric method for selective detection of long-chain lipid pyrophosphate monoesters over pyrophosphate diesters with the use of **Zn21**.

A particular scientific attention was devoted to two-photon active Zn(II) fluorophores, achieving their excited states by using photons of nearly half the energy of the corresponding one-photon transition, and thus being suitable for their applications as biosensors in two-photon confocal microscopy. The studies of two-photon properties were performed for **Zn11** [60], **Zn18** [83], **Zn4**–**Zn6**, **Zn19** [78], and **Zn20** [79]. Relative to the free ligands, a noticeable enhancement of TPA response was evidenced for **Zn18** and **Zn20**. Exemplarily, in the wavelength range (680–920 nm), the TPA of **Zn18** was enhanced to 140 GM, showing a 3-fold increase in relation to the free ligand. Using this complex, an electrochemiluminescence sensor with good biocompatibility and long-term stability was fabricated to detect ctDNA [83]. Regarding the complexes **Zn4**–**Zn6** and **Zn19** bearing the same ligand, it can be noticed that the symmetrical Zn(II) complex **Zn19** possesses significantly enhanced third-order NLO response in the NIR region compared to **Zn4**–**Zn6**, which was assigned to stronger ILCT transitions [78]. The complex **Zn19** was successfully utilized as a two-photon probe for cellular lipid membranes, and its high sensitivity and selectivity were confirmed by two-photon confocal and transmission electron microscopies [78]. Promising results were also reported for **Zn20**. With the use of the two-photon confocal and transmission electron microscopies, this complex was evidenced to accumulate in mitochondria in living cells and migrate to the nucleolus in apoptotic cells. Thanks to this dual-functional probe, this system can be regarded as an excellent indicator for monitoring cell death processes [18,79].

## 4. Fe(II), Ru(II), and Os(II) Complexes

**Scheme 3:**
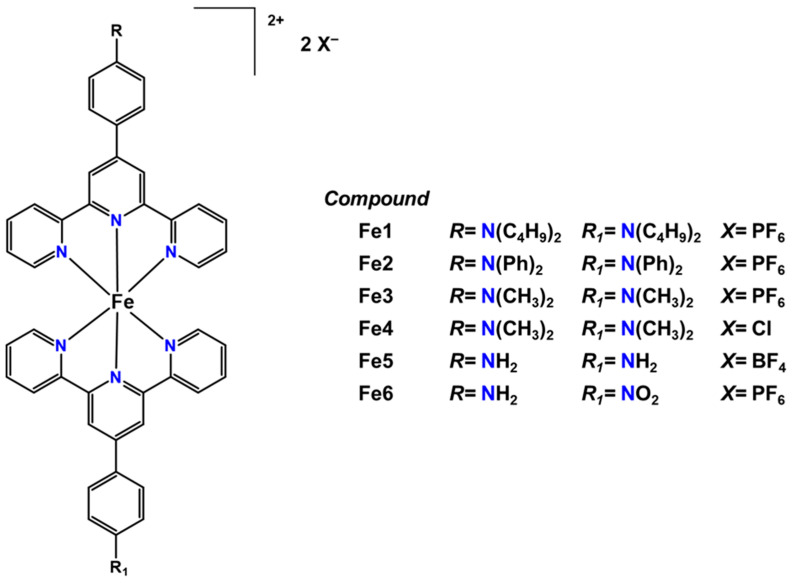
Iron(II) complexes with amine-modified R-C_6_H_4_-terpy ligands.

Optical and electrochemical properties of Fe(II) complexes with amine-functionalized R-C_6_H_4_-terpy ligands were the subject of investigations reported in [73,85,86,87,88,89,90,91]. In all iron-based terpyridine complexes (Scheme 3), the Fe(II) ion is in the low-spin electronic configuration (t_2g_^6^), in agreement with acting of R-C_6_H_4_-terpys as π-acceptor ligands due to possessing low-lying π* orbitals [92,93].

The most important advantages of [Fe(R-C_6_H_4_-terpy)_2_]X_2_ are convenient and efficient synthesis through the reaction of Fe(II) salts with appropriate R-C_6_H_4_-terpy ligands, high stability, intense visible light absorptivity, and rich electrochemical properties. The visible region in the UV-Vis electronic spectra of purple complexes [Fe(R-C_6_H_4_-terpy)_2_]X_2_ are dominated by the intense band above 500 nm attributed to spin-allowed MLCT transitions, in accompany with distinctive absorption at ~420 nm due to ILCT transitions of R-C_6_H_4_-terpy in the case of **Fe1**–**Fe4** (Table S9). Compared to the model chromophore [Fe(terpy)_2_]X_2_, the longest wavelength absorption is considerably more intense and slightly red-shifted. This effect was rationalized by an energy rising of HOMO and increased contribution of the R-C_6_H_4_- unit in the HOMO (Table S9). Nonlinear optical properties were confirmed for **Fe1**, which show TPA cross-section of 4699.19 GM at 800 nm wavelength and four-photon cross-section of 3.26 × 10^−73^ cm^8^ s^3^ photon^−3^ at 2200 nm, indicating its potential for biological applications [91].

Bis-terpyridyl Fe(II) complex with 4′-(4-anilino)-2,2′:6′,2″-terpyridine was successfully tested to construct multicomponent architectures by its coupling with a multifunctional organic reagent. Among others, pyromellitic dianhydride in combination with pyridine–acetic anhydride, terephthaloyl chloride, and adipoyl chloride were used. The resulting macromolecules and metallo-oligomers were investigated in view of their magnetic and optical features [85,86].

**Figure 3:**
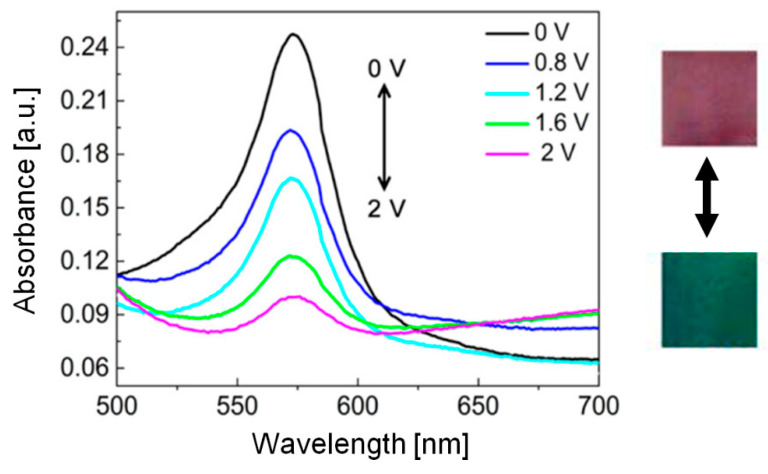
Absorption spectral changes in the electro-polymerized film **Fe2** within the voltage range from 0 to 2 V, along with photographs of films in the neutral (purple) and oxidized (blue) states. The figure was readapted with permission from the work [88]. Copyright © 2015 American Chemical Society.

The complex **Fe2** was found to exhibit high electropolymerization activity within the voltage range from 0 to 2 V, accompanied by the reversible color change from purple for its neutral state to blue for the oxidation one (Figure 3). The long memory time (~12 min), defined as the time that the electrochromic film can keep the half absorbance of the oxidation state after turning off the potential, was associated with the strong donating ability of the triphenylamine unit, which effectively promotes intraligand charge transfer from Ph_3_N^−^ to Fe^3+^ [88].

The latest investigations of [Fe(R-C_6_H_4_-terpy)_2_]X_2_ have been largely focused on their photocatalytic activity. Possessing strong visible absorptivity and rich redox properties, these systems are regarded as ideal candidates to develop efficient catalysts. Importantly, they fulfil the requirements of green technology, offering numerous environmental and economic benefits. Most importantly, iron is an environmentally friendly element, with high abundance (5%) in Earth’s crust [94]. The complex **Fe3** was successfully utilized in combination with diphenyliodonium hexafluorophosphate and N-vinylcarbazole (0.2%/2%/3%) as a photoinitiating system for free radical polymerization of acrylates and free radical-promoted cationic polymerization of epoxides upon exposure to a visible violet LED at 405 nm. The final conversion of the 3,4-epoxycyclohexylcarboxylate (EPOX) and trimethylolpropane triacrylate (TMPTA) was ~25% after irradiation for 800 s for EPOX and 400 s for TMPTA [89]. The authors of [90] tested the complex [Fe(Me_2_N-C_6_H_4_-terpy)_2_]Cl_2_ (**Fe4**) in combination with an organic thermally activated delayed fluorescence dye 4CzIPN for photocatalytic CO_2_ reduction to CO upon visible-light irradiation in DMF/H_2_O (*v*/*v* = 3:2). In this fully noble metal free photocatalytic system, the Fe(II) complex was employed as catalyst, while 4CzIPN acted as the photosensitizer. Solar-driven CO_2_ reduction is regarded as one of the most promising approaches for CO_2_ utilization. Compared to other investigated iron-based terpyridine complexes [Fe(Cl-C_6_H_4_-terpy)_2_]Cl_2_ and [Fe(Ph-terpy)_2_]Cl_2_, **Fe4** was found be the most efficient (turnover number of CO = 6320, turnover frequency = 127 min^−1^, selectivity = 99.4%, apparent quantum yield = 9.5% at 440 nm). It was postulated that the amine electron-donating group is responsible for the enhancement of the nucleophilicity of the Fe(II) center, which seems to be essential for the whole catalytic cycle. 4CzIPN was demonstrated to be more efficient than the well-known photosensitizer [Ru(bipy)_3_]^2+^.

The photocatalytic activity of **Fe2** and **Fe3** was evaluated for the degradation of methylene blue, and the photocatalytic degradation efficiencies of **Fe2** and **Fe3** were found to be 59.16 and 56.3%, respectively. The higher photocatalytic activity of **Fe2** was attributed to the presence of two aromatic rings that are a rich source of delocalized electrons [73].

**Scheme 4:**
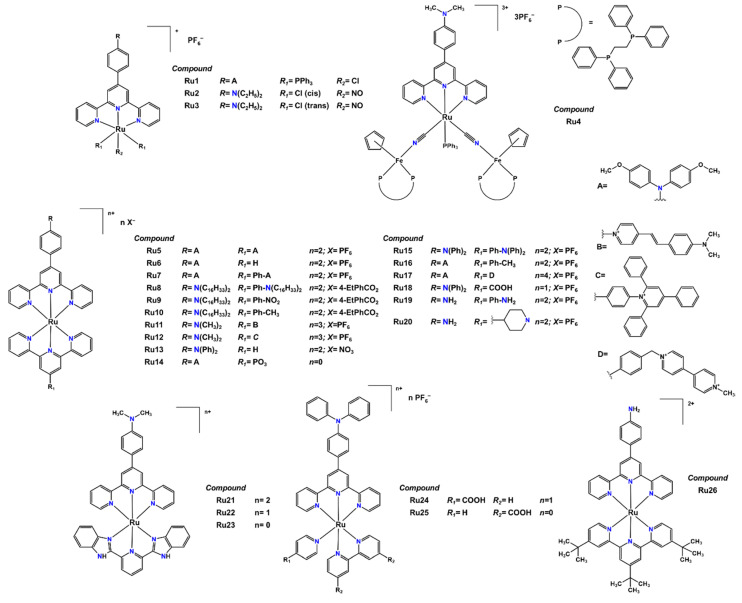
Ruthenium(II) complexes with amine-modified R-C_6_H_4_-terpy ligands.

Mono- and bis-terpyridyl ruthenium(II) complexes with amine-modified R-C_6_H_4_-terpy ligands investigated in view of their optical and electrochemical behavior are summarized in Scheme 4 (Tables S10 and S11). The most studied Ru(II) complexes with this ligand type are bis-terpyridyl systems. In contrast to mostly investigated tris-bipyridine Ru(II) complexes, the geometry of [Ru(R-C_6_H_4_-terpy)_2_]^2+^ does not lead to isomeric mixtures. Furthermore, it is possible to design species in which substituents of different functionality lie in opposing directions.

By analogy to iron-based terpyridine complexes, the incorporation of the electron-donating amine group was found to induce a significant bathochromic-hyperchromic effect on the absorptivity of these systems in relation to appropriate unsubstituted Ru(II) chromophores (Tables S10 and S11). In relation to related [Fe(R-C_6_H_4_-terpy_2_)]^2+^, the longest wavelength absorption of bis-terpyridyl ruthenium(II) complexes is considerably blue-shifted due to a much stronger ligand field for the second-row transition metals [95].

Strong visible absorptions of Ru(II) complexes with amine-functionalized R-C_6_H_4_-terpy ligands are attributed to the superposition of MLCT and ILCT transitions [57,87,96,97,98,99,100]. Upon protonation of amine groups, when pendant amine substituents lose their donating ability, the progressive disappearance of the ILCT absorption band can be observed, providing evidence for the contribution of MLCT absorptions [96].

The assignment of the visible absorptions of these systems to both MLCT and ILCT transitions was further supported by electrochemical studies. Contrary to the well-known model chromophore [Ru(terpy_2_)]^2+^, which shows the oxidation wave assigned to Ru^II^/Ru^III^ process and reduction wave due to the one-electron reduction of terpy, bis-terpyridyl ruthenium complexes [Ru(R-C_6_H_4_-terpy_2_)]^2+^ possess richer electrochemistry, displaying additional amine oxidation potentials [57,96,98,100,101,102]. In agreement with the stronger stabilization of the metal-centered HOMO in the 4*d* compared to 3*d* metal complexes, the Ru(II) ion is harder to oxidize than the Fe(II) ion (Tables S8–S11).

**Figure 4:**
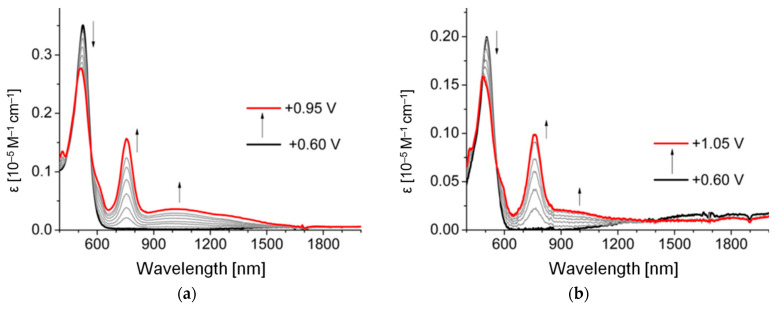
Absorption spectral changes of **Ru5** (**a**) and **Ru6** (**b**) upon stepwise electrolysis at an ITO glass electrode in CH_2_Cl_2_ (↓—absorption decrease, ↑—absorption increase). The spectra are readapted with permission from the work [98]. Copyright © 2014 American Chemical Society.

The complexes **Ru1** [102], **Ru4** [103]_,_ **Ru5**, **Ru6**, and **Ru7** [98] were subjected to the successive oxidation, and absorption properties of the resulting mono- and di-oxidized forms were investigated in the UV-Vis-NIR range in order to investigate amine–amine electronic coupling, verify the occurrence of inter-valence charge transfer (IVCT) transitions, and determine their sensitivity to the electron-donating ability of the remote amine group. Most remarkably, the mono-oxidized form of **Ru5** was evidenced to show the charge transfer band at 1000 nm originating from the ruthenium ion to the ammonium radical cation of the MeOPh_2_N-C_6_H_4_-terpy ligand, while the reversed charge transfer, that is from metal center to the ammonium radical cation of the (MeOPh)_2_N-terpy ligand represented by the band at 1380 nm, occurred for two-oxidized form, confirming IVCT transitions (Figure 4).

The possibility to distinguish mono- and di-oxidized states of **Ru5** by the NIR absorption signals gives rise to their potential applications as electrochromic materials and molecular logic gates [98]. A reversibly distinguishable color, from orange-red to yellow, was also confirmed under voltages between 0 and 2 V for polymer films {[Ru(Ph_2_N-C_6_H_4_-terpy)_2_](PF_6_)_2_}_n_ obtained by the electrochemical polymerization [88].

In the cyanide-bridged trinuclear Fe–Ru–Fe complex **Ru4** [103], the visible absorption (with λ_max_ at ~420 nm) was identified as MLCT, and three reversible redox waves were assigned to Fe^II^–Ru^II^–Fe^II^/ Fe^II^–Ru^II^–Fe^III^, Fe^II^–Ru^II^–Fe^III^/ Fe^III^–Ru^II^–Fe^III^, and Fe^III^–Ru^II^–Fe^III^/ Fe^III^–Ru^III^–Fe^III^. The new energy bands at 815 and 1530 nm, appearing after one-electron oxidation, were attributed to Fe^II^–Fe^III^ and Ru^II^–Fe^III^ MMCT transitions. The further oxidation of **Ru4** resulted in the disappearance of the band in the Vis-NIR region. The strong band attributed to Ru^II^–Fe^III^ MMCT transitions was observed at 800 nm. All these findings indicate a noticeable impact of the remote amine substituent on the MMCT absorptions in terpyridyl heterometallic structures.

The coordination of push–pull ligands to Ru(II) ions was also reported to increase their quadratic hyperpolarizability [96,104,105]. The beneficial impact of the remote electron-donating group on nonlinear optical properties was widely discussed for complexes **Ru8**, **Ru9**, and **Ru10** [104], **Ru11** [96], and **Ru21**–**Ru23** [105]. The metal ion in **Ru9** was found to act as a bridge between the ligands bearing donor and acceptor groups, respectively. It resulted in its enhanced quadratic hyperpolarizability in relation to the homoleptic systems **Ru8** [104]. The hyperpolarizability of **Ru11** was evidenced to be almost the same as for the free R^1^-terpy ligand (Scheme 4). In the case of the last systems **Ru21**–**Ru23**, the stepwise deprotonations of the benzimidazole resulted in an additional increase in electron density at this moiety, subsequently leading to an efficient second-order NLO switching [105]. The third-order NLO behavior was investigated for the linear polyad of the donor–photosensitizer–acceptor type, **Ru12**, with pyridino (acceptor) and amino (donor) nitrogen atoms *trans*-located at equal distances from the metal ion. Such systems are expected to form a charge-separated excited state [106].

Regarding photoluminescence properties, the vast majority of the investigated Ru(II) complexes with amine-modified R-C_6_H_4_-terpy ligands were found to be non-emissive at room temperature, most likely due to the small energy gap between ^3^MLCT and ^3^MC, and thus, radiationless deactivation via the ^3^MC pathway. The detectable emission at room temperature was confirmed for **Ru13** in MeCN (λ_ex_= 493 nm, λ_em_ = 660 nm, τ = 1.1 ns, Φ = 1.8 × 10^−4^) [57], **Ru14** in EtOH (λ_em_ = 686 nm) [99], **Ru11** in MeCN (λ_ex_ = 469 nm, λ_em_ = 612 nm, Φ = 0.31 × 10^−2^) [96]. As can be seen, a drawback of Ru(II) coordination compounds is also a short excited-state lifetime. All these features make these systems less appealing for applications in photocatalysis. Noteworthy, the emission of **Ru15** was found to be excitation-dependent, showing fluorescence at 467 nm upon excitation at 375 nm and phosphorescence at 633 nm when it is excited at 430 nm. The femtosecond fluorescence upconversion studies of this complex revealed the ultrafast component assigned to the decay of ^1^MLCT emission and intersystem crossing to the ^3^MLCT excited state [100]. For complexes **Ru16** and **Ru17**, transient absorption studies revealed the signals (at ~740 nm) typical of the amine radical cation, indicating formation of the triplet charge-separated excited state in these systems. The TA bands of **Ru16** and **Ru17** were found to decay with lifetimes of 450 ns and 100 ns, respectively. For both complexes, the characteristic ^3^CT emission was observed in the frozen propionitrile–butyronitrile (at 90 K, λ_em_ = 656 nm, τ = 13.9 µs for **Ru16** and λ_em_ = 656 nm, τ = 13.4 µs for **Ru17**). The temperature increase induces a significant decrease in the emission intensity and lifetime of **Ru16** and **Ru17** (at 150 K, λ_em_ = 678 nm, τ = 0.4 µs for **Ru16** and λ_em_ = 675 nm, τ = 0.1 µs for **Ru17**). At room temperature, the complex **Ru17** becomes non-emissive (λ_em_ = 676 nm, τ = 0.04 µs for **Ru16**) [101].

Photoinduced charge separation processes of **Ru14** [97,99], **Ru24**, **Ru25**, and **Ru18** [107] were explored in dye-sensitized solar cells (DSSCs), where Ru(II) sensitizer was anchored at the TiO_2_ surface by phosphonate or carboxylate groups. The sensitizer [Ru(MeOPh)_2_NC_6_H_4_terpy)(terpyPO_3_)] was reported not to allow the efficient charge separation at the TiO_2_ surface owing to a fast recombination process D^+^–S|(e^−^)TiO_2_→D–S|TiO_2_ [97]. Further investigations [99] revealed that the efficiency of the photoinduced charge separation in **Ru14** may be noticeably improved by an elongation of the D–S distance and, thus, variations in the orientation of molecules in the monolayer. The explorations of DSSCs with **Ru24**, **Ru25**, and **Ru18** evidenced that Ru(II) sensitizers anchored in a more upright position and with a larger number of carboxylate groups induce better overall photoconversion efficiency [107].

The authors of [108] presented a new strategy to improve the photocatalytic activities of bis-terpyridyl ruthenium(II), despite their short excited-state lifetimes. A significant improvement in the photocatalytic efficiency in the Heck reaction was achieved by the attachment of **Ru19** to graphene oxide. With the use of DFT calculations, the hole transfer from the metal complex moiety to graphene oxide was postulated as the initial step in the photocatalytic cycle.

Nitrosyl Ru(II) complexes **Ru2** and **Ru3** were investigated in view of their ability to deliver NO in a controlled manner by visible irradiation. NO is a signaling molecule that participates in numerous physiological processes, such as neurotransmission, immunology, vasodilatation, and angiogenesis. On the basis of spectrophotometrical studies, it was evidenced that the photocleavage of the Ru–NO bond is affected by the ligand arrangement, and the better NO releasing was demonstrated for the *cis*-(Cl, Cl)-isomer, where the nitrosyl ligand is *trans*-located to the chloride one [109].

**Scheme 5:**
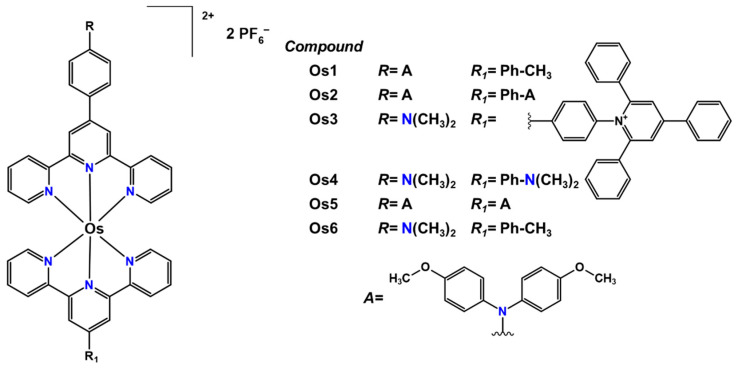
Osmium(II) complexes with amine-modified R-C_6_H_4_-terpy ligands.

To date, only bis-terpyridyl osmium(II) complexes with amine-modified R-C_6_H_4_-terpy ligands (Scheme 5) were reported [106,110,111,112].

By analogy to related bis-terpyridyl Ru(II) complexes, the electrochemical and photophysical properties of bis-terpyridyl Os(II) complexes are significantly tuned by the remote redox-active groups (Tables S12 and S13). In contrast to Ru(II) complexes, due to the enhanced spin–orbit coupling of the third-row elements compared to the second-row elements, Os(II) complexes display triplet–triplet transitions (600–800 nm), not observed in related Ru(II) complexes. In the range 450–600 nm, high similarity in absorption properties of Os(II) and Ru(II) bis-terpyridyl complexes is observed [106,110,111,112].

Detectable emission at room temperature was revealed for compounds **Os1**–**Os4.** It appears in the red or NIR light range [110,112].

Stepwise oxidations of **Os1**, **Os2**, and **Os5** revealed further differences between Ru(II) and Os(II) analogues. Most importantly, a stronger electronic coupling was confirmed between (MeOPh)_2_N-C_6_H_4_-terpy and Os(terpy) moieties owing to the lower Os(III/II) potential compared to Ru(III/II) one. No presence of osmium-mediated amine–amine electronic coupling was evidenced for Os(II) systems with two amine groups [110].

## 5. Cr(III) Complexes

**Scheme 6:**
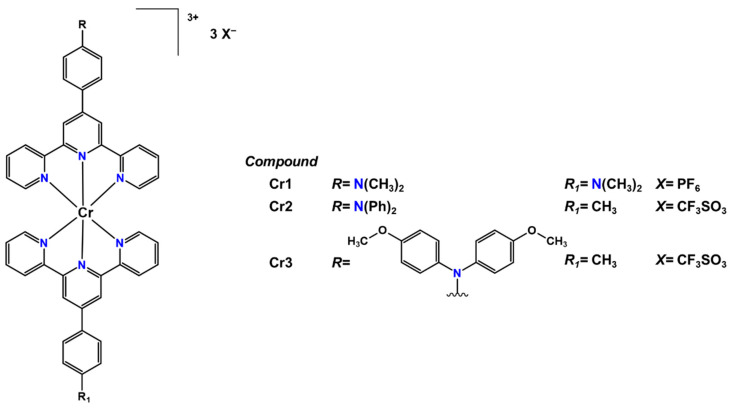
Chromium(III) complexes with amine-substituted R-C_6_H_4_-terpy ligands.

In recent years, the photophysics of Cr(III) polypyridyl chromophores has received renewed interest. These systems are regarded as cheaper alternatives to Ru(II) photosensitizers. Chromium is an inexpensive, earth-abundant metal, and its coordination compounds possess appealing photophysical properties and electrochemical activity [113,114,115,116,117,118,119,120,121]. Contrary to well-known Cr(III) bipyridyl systems, however, terpyridine Cr(III) complexes remain underexplored. In particular, chromium(III) compounds with amine-substituted R-C_6_H_4_-terpy ligands [122,123] have received little scientific attention. To date, only three compounds of this type have been reported (Scheme 6).

**Figure 5:**
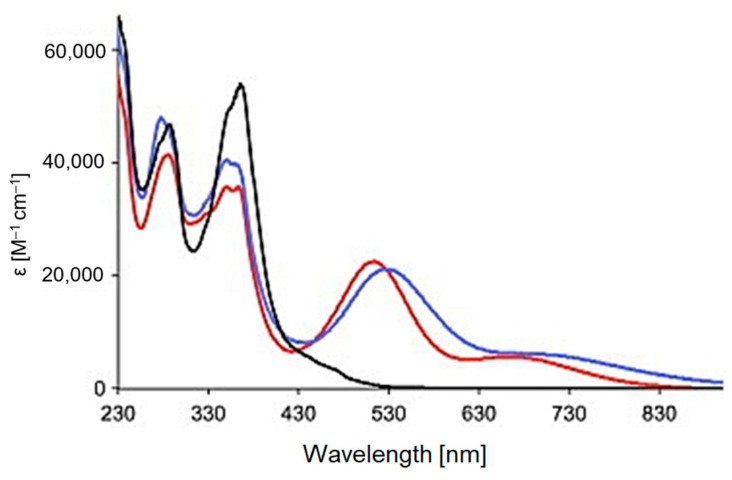
The absorption properties of **Cr2** (red line) and **Cr3** (blue line) in relation to the model chromophore [Cr(CH_3_-C_6_H_4_-terpy)_2_]^3+^ (black line). The spectra are readapted with permission from the work [122].

Compared to the unsubstituted chromophores, [Cr(terpy)_2_]^3+^, [Cr(Ph-terpy)_2_]^3+^ and [Cr(CH_3_-C_6_H_4_-terpy)_2_]^3+^ (Table S14), the complexes **Cr1**, **Cr2** absorb energy in a noticeably wider range of wavelengths, up to 830 nm (Figure 5, Table S15). The molar extinction coefficient of the absorption at 532 nm, dominated in the visible region of **Cr1**, exceeds 55,000 M^−1^ cm^−1^ [123].

The ILCT absorptions of the heteroleptic systems **Cr2** and **Cr3** were found to show negative solvatochromism when they were examined in CHCl_3_, CH_2_Cl_2_, Me_2_CO, EtOH, and MeCN solutions. In turn, in H_2_O, MeOH_,_ DMSO, and DMF_,_ the terpy ligands of these systems were evidenced to be labile, and variations in their UV-Vis spectra were supportive of the formation of [Cr(R-terpy)(solvent)_3_]^3+^ [122].

The emission studies were performed only for the homoleptic compound **Cr1** and revealed that it is non-emissive upon excitation at wavelengths of the ILCT band. Worthy of note, the introduction of the less electron-donating methoxy group resulted in an emission enhancement and elongation of excited-state lifetime of [Cr(Me)-C_6_H_4_-terpy)_2_]^3+^ (λ_em_ = 785 nm, τ = 600 ns) relative to [Cr(terpy)_2_]^3+^ (λ_em_ = 770 nm, τ = 140 ns) [123].

## 6. Ir(III) Complexes

**Scheme 7:**
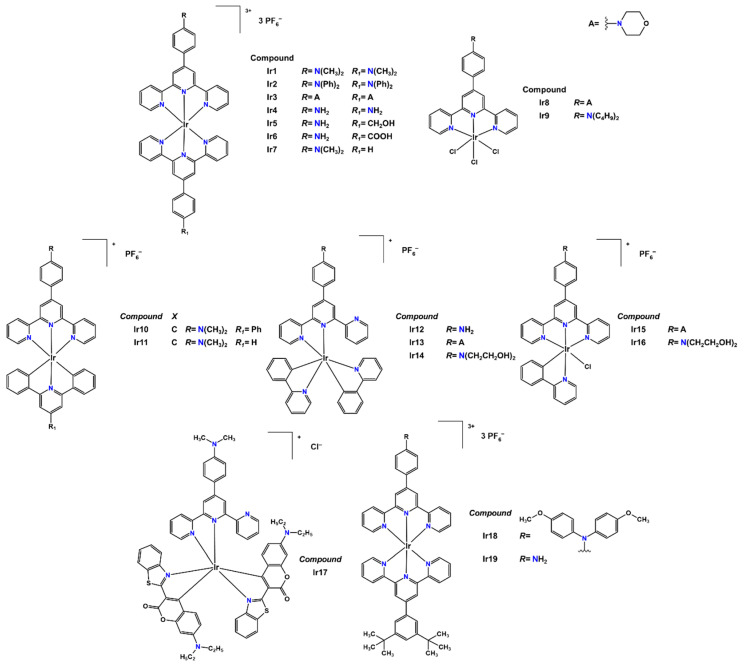
Iridium(III) complexes with amine-substituted R-C_6_H_4_-terpy ligands.

The role of the remote amine group in controlling of photophysical behavior was investigated for the series of homo- and heteroleptic bis-terpyridyl [Ir(R-C_6_H_4_-terpy-κ^3^N)_2_]^3+^, mono-terpyridyl [IrCl_3_(R-C_6_H_4_-terpy-κ^3^N)_2_]^3+^, as well as mono- and bis-cyclometalated Ir(III) complexes of general formula [Ir(C^∩^N^∩^C)_2_(R-terpy-κ^3^N)]^+^, [IrCl(N^∩^C)(R-terpy-κ^3^N)]^+^, and [Ir(N^∩^C)_2_(R-terpy-κ^2^N)]^+^ (Scheme 7 and Table S17).

**Figure 6:**
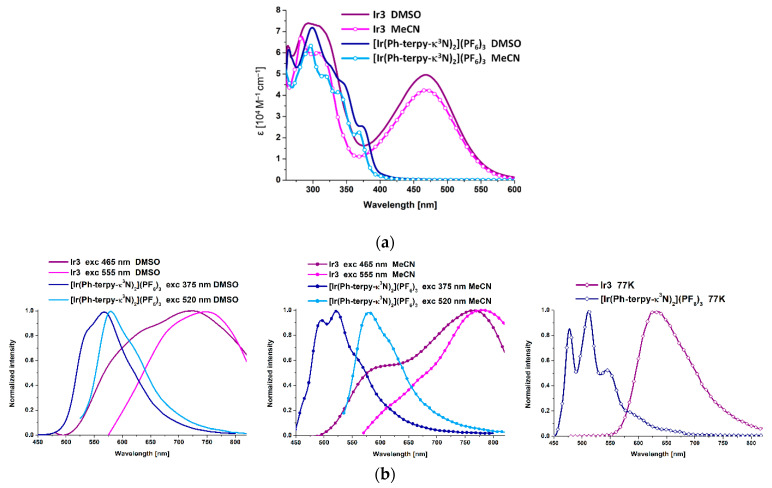
Absorption (**a**) and emission (**b**) properties of **Ir3** in relation to the model chromophore [Ir(Ph-terpy-κ^3^N)_2_](PF_6_)_3_. The spectra are readapted from our previous work [124].

The absorption and emission properties of homo- and heteroleptic bis-terpyridyl Ir(III) complexes were found to be markedly impacted by the attachment of the amine group to the central pyridine ring of the terpy backbone via the phenyl linker. The strongly electron-donating amine group was shown to alter the nature of the HOMO orbital and noticeably reduce the HOMO–LUMO gap of [Ir(R-C_6_H_4_-terpy-κ^3^N)_2_]^3+^ relative to the parent chromophore [Ir(Ph-terpy-κ^3^N)_2_]^3+^ [124,125,126,127]. The HOMO of the amine-substituted systems is predominately contributed by the amine group and phenylene linker, while LUMO, likewise in the unsubstituted chromophore, largely resides on the terpy core. Consequently, deeply red [Ir(R-C_6_H_4_-terpy-κ^3^N)_2_]^3+^ complexes display a new intense absorption band in the visible region (425–557 nm, Table S17) corresponding to ILCT excitations, not observed in the UV-Vis spectrum of the yellow model chromophore (Figure 6).

Consistent with the stronger electron-donating properties of –NMe_2_ relative to –NPh_2_, the absorption maximum of **Ir1** is red-shifted (by 10 nm in CH_2_Cl_2_) in relation to **Ir2** [125]. The difference between the homo- and heteroleptic bis-terpyridyl Ir(III) complexes bearing H_2_N-C_6_H_4_-terpy (**Ir4**, **Ir5**, and **Ir6**) mainly concerns the absorption intensity of the ILCT band, which is almost two-times larger in the case of the homoleptic complex (Table S17), where two amine groups make contribution into ILCT excitations [126]. The sensitivity of the ILCT absorption of [Ir(R-C_6_H_4_-terpy-κ^3^N)_2_]^3+^ to variations in the solvent nature and pH environment was discussed in [125,126]. The formation of the ILCT excited state in these systems generally evokes a slight positive solvatochromism, but changes in ILCT band maximum are difficult to analyze owing to the impact of other solvent interactions, including hydrogen bonding. At low pH, due to protonation of the amine group, charge transfer processes can be deactivated, leading to the disappearance of the ILCT band [126].

Dramatic changes are also observed in the emission characteristics of [Ir(R-C_6_H_4_-terpy-κ^3^N)_2_]^3+^ and [Ir(R^1^-C_6_H_4_-terpy-κ^3^N)(R^2^-C_6_H_4_-terpy-κ^3^N)]^3+^ in comparison to [Ir(Ph-terpy-κ^3^N)_2_]^3+^ (Table S17). Upon excitation at the ILCT band, the complexes **Ir1**, **Ir2**, and **Ir3** were found to show emission in the NIR region, originating from ^3^ILCT and markedly red-shifted relative to the photoluminescence of the model complex, occurring in the green light region. For all these systems, **Ir1**–**Ir7**, the attachment of the remote amine group and formation of ^3^ILCT excited state resulted in the disappearance of a weak vibronic progression in the emission profile and noticeable decrease in the quantum yields and lifetimes relative to the emission of [Ir(Ph-terpy-κ^3^N)_2_]^3+^. The stronger and longer-lived emission of [Ir(Ph-terpy-κ^3^N)_2_]^3+^ was attributed to the triplet ligand-centered (^3^IL) or mixed ^3^IL–^3^MLCT excited states [124,128]. The different nature of the lowest triplet excited states in complexes **Ir3** and [Ir(Ph-terpy-κ^3^N)_2_](PF_6_)_3_ was further evidenced by transient absorption (TA) spectroscopy. The fs-TA data of [Ir(Ph-terpy-κ^3^N)_2_](PF_6_)_3_ are supportive of a mixed IL-MLCT character of the lowest triplet excited state, which undergoes switching into ^3^ILCT after the attachment of the morpholinyl group [125].

The excitation of acetonitrile and water solutions of **Ir4**, **Ir5**, and **Ir6** at 350 nm resulted in a structureless emission band with the maximum and lifetime falling in the range 495–506 nm and 1.1–19 μs, respectively (Table S17). The presence of the –NH_2_ group generally resulted in a weaker emission compared to the parent complex, and a further noticeable quenching effect was observed in water solution of **Ir4** and **Ir5** [126]. As reported in [129], the formation of ILCT state is expected to be retarded in water owing to hydrogen bonding.

Investigations of **Ir8** revealed that the photophysical behavior of mono-terpyridyl Ir(III) systems is significantly less affected by the remote amine substituent. The absorption and emission properties of **Ir8** were attributed to ^1^MLLCT–^1^ILCT and ^3^MLLCT–^3^ILCT excited states, respectively. The contribution of ILCT was clearly manifested in an enhancement of the visible absorptivity and bathochromic emission shift relative to the parent chromophore. The red shift of the emission band was accompanied by a slight decrease in the lifetime [124]. For the **Ir9** complex, the second-order NLO response was investigated. It was revealed that the nonlinear optical properties of the mono-terpyridyl system [IrCl_3_(R-C_6_H_4_-terpy-κ^3^N)_2_]^3+^ are also contributed by ILCT and MLCT transitions [130].

The cyclometalation effect in Ir(III) complexes bearing amine-substituted R-C_6_H_4_-terpy ligands was demonstrated in [127]. The replacement of Ph-terpy in **Ir7** by Ph-dppy (dppy: 2,6-diphenylpyridine) was found to induce a significant blue shift in absorption and enhancement of emission properties. While the complex **Ir7** was reported to be non-emissive in solution, **Ir10** displays broad and oxygen-sensitive phosphorescence in the range 600–800 nm. Importantly, the cyclometalation was evidenced to increase markedly photosensitization and photocatalytic efficiency of this type of Ir(III) complexes. In particular, the complex **Ir10** generates ^1^O_2_ much more effectively and shows significantly higher NADH photocatalytic activity than **Ir7** upon light irradiation. The in-cell photocatalytic efficiency is crucial for solving the drug resistance and hypoxia problem in anticancer therapy. The studies confirmed that **Ir7** is able to act as both I/II photosensitizer and photocatalyst. Located in mitochondria, it shows strong photocytotoxicity against the cisplatin-resistant A549/DDP cancer cells under both normoxia and hypoxia [127].

Comparative analysis of **Ir11** and [Ir(Ph-terpy-κ^3^N)(dppy)]^+^ revealed that the appended –NMe_2_ group did not change noticeably the absorption and emission energies, but evoked a dramatic increase in the molar excitation coefficients of ^1^LLCT/^1^MLCT absorption bands in the range 400–600 nm and resulted in a higher emission quantum yield and longer lifetime of **Ir11** relative to [Ir(Ph-terpy-κ^3^N)(dppy)]^+^. The emitting state of **Ir11** was assigned as ^3^ILCT with a minor contribution of ^3^π-π/^3^MLCT/^3^LLCT [131].

Our research group performed comprehensive studies for mono- and bis-cyclometalated Ir(III) complexes bearing bi- and tridentate coordinated morph-C_6_H_4_-terpy ligand (**Ir13**, **Ir15**). We evidenced that the role of the morpholinyl group in controlling emission behavior is totally different for [IrCl(Ph-py)(R-C_6_H_4_-terpy-κ^3^N)]PF_6_ and [Ir(Ph-py)_2_(R-C_6_H_4_-terpy-κ^2^N)]PF_6_. In the case of bis-cyclometalated Ir(III) complexes, the morpholinyl substituent was found to evoke rather marginal perturbations in the emission energy, but it had a positive impact on the emission lifetime, resulting in its prolongation [132]. The same trend was observed for **Ir12** in relation to [Ir(Ph-py)_2_(terpy-κ^2^N)]PF_6_ [133]. In contrast, the incorporation of the morpholinyl group in mono-cyclometalated Ir(III) systems induced dramatic variations in the emission characteristics. The complex **Ir15** is a rare example of dual-phosphorescence Ir(III) systems. The high- and low-energy emissions were evidenced to occur from ^3^MLLCT and ^3^ILCT excited states, respectively. Further structural modifications of terpy and cyclometalating ligands may be crucial in a better understanding of the dual-emission phenomena in [IrCl(Ph-py)(R-C_6_H_4_-terpy-κ^3^N)]PF_6_ systems.

For both **Ir15** and **Ir13**, the appended morpholinyl group was found to markedly enhance the absorptivity in the range 350–550 nm, consistent with the involvement of ^1^ILCT transitions. Owing to the larger terpy conjugation in **Ir15**, the lowest energy absorption band of the mono-cyclometalated Ir(III) system is noticeably red-shifted relative to that of **Ir13** [132]. The same trend in absorption properties was evidenced for the pair **Ir16** and **Ir14**. By analogy to their absorption characteristics, the structureless emission band of **Ir16** occurs at longer wavelengths compared to the bis-cyclometalated Ir(III) complex. Striking differences between **Ir16** and **Ir14** were also found in view of their nonlinear optical properties, generation of ROS species by type I and II pathways, and interactions with DNA and BSA. Two-photon absorption and excited fluorescence were demonstrated only for the bis-cyclometalated Ir(III) complex (215 GM at 890 nm), which was also evidenced to be more effective in producing O_2_**^·^**^−^. In contrast, the complex **Ir16** showed larger ^1^O_2_ generation. Due to the planarity of the terpy framework, the mono-cyclometalated Ir(III) system showed greater DNA and BSA binding affinity [134].

The photo-chemotherapeutic activity was examined for **Ir17** [135], which was evidenced to generate singlet oxygen via both I and II pathways and induce photo-catalytic oxidation of cellular coenzymes NAD(P)H (NADH and NADPH). The photo-oxidation of NAD(P)H occurred with H_2_O_2_ generation. As the authors highlighted, it was the first report of green light-induced NAD(P)H photo-oxidation (525 nm) by a metal complex in an aqueous environment. The complex **Ir17**, preferably localized in the mitochondria with high intracellular accumulation and retention efficiency, was confirmed to be an effective photo-cytotoxic agent towards different cancer cells (human cervical (HeLa), epidermoid carcinoma (A431), human nasopharyngeal epithelial (NP69), and mouse melanoma (B16)) under light irradiation. Its increased lipophilicity was assigned to the coumarin-based cyclometalated ligand. Despite very promising indexes, however, **Ir17** was found to have lower medicinal potential than its analogue bearing two appended trifluoromethyl groups, showing the photoindex against HeLa cancer much higher than the clinically used 5-aminolevulinic acid photosensitizer. Regarding photophysical properties, **Ir17** was found to show viscosity-dependent phosphorescence, with higher emission in a more viscous environment. It was correlated with the rotation on N,N-diethyl in the coumarin moiety [135].

## 7. Mn(I) and Re(I) Carbonyl Complexes

**Scheme 8:**
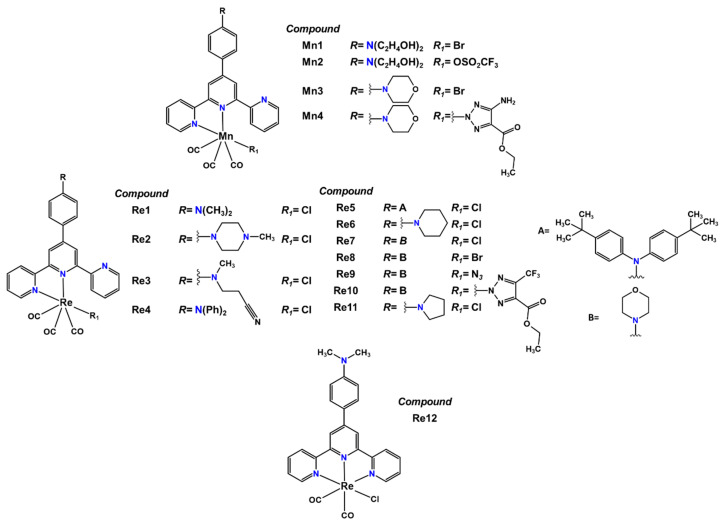
Manganese(I) and rhenium(I) carbonyl complexes with amine-modified R-C_6_H_4_-terpy ligands.

Terpyridyl Mn(I) and Re(I) carbonyl complexes with the remote amine group, reported so far, are demonstrated in Scheme 8 and Tables S18–S21.

All manganese(I) carbonyl complexes with amine-substituted R-C_6_H_4_-terpy ligands, coordinated to the central metal ion in a bidentate coordination mode (κ^2^N), were designed and evaluated as carbon monoxide releasing molecules (CORMs) [136,137]. Such compounds are regarded as very promising candidates for delivering low amounts of CO for therapeutic purposes. In low concentrations, CO was found to play significant physiological roles as a small molecule messenger and has a beneficial effect on cardiovascular diseases, cytotoxicity towards cancer cells, bacterial infections, and inflammatory disorders [138,139]. The CO release may be triggered using different methods, including photoactivation, ultrasound (US) irradiation, thermal activation, pH change, or enzymatically [136,137]. The ability of Mn(I) complexes with 4′-p-N,N-bis(2-hydroxyethyl)aminobenzyl-2,2:6,2″-terpyridine to release CO was investigated under single-photon excitation with visible light (405 and 451 nm) and two-photon excitation with near-infrared (NIR) light (750 and 800 nm). NIR wavelengths fall in the phototherapeutic window of mammalian tissues and offer deeper tissue penetration. In the case of single-photon photolysis, a two-stage photochemical process was evidenced, comprising the release of one CO in the first stage and two more COs during the second stage. The ability of **Mn1** and **Mn2** to release CO was also confirmed upon NIR excitation, but the mechanism of this process was found to be ambiguous. It was also reported that chromophores with cross-sections stronger than those for the reported Mn(I) systems are required for practical applications under physiological conditions [136]. The capability of Mn(I) complexes with 4′-morpholinyl-C_6_H_4_-terpy ligand to act as CORMs was confirmed only under excitation with visible light (468 and 525 nm). It was demonstrated that Br^−^, providing larger electron density to the Mn(I) ion than the triazolate ligand, accelerates the CO release. In turn, the triazolate ligand makes terpyridyl Mn(I) complexes more soluble in organic solvents relative to the bromide analogues [137].

The structural modification of Ph-terpy with –NMe_2_ moiety was found to induce dramatic changes in absorption and emission properties of **Re1** in DMF in relation to the parent chromophore [ReCl(CO)_3_(Ph-terpy-κ^2^N)]. These distinct variations in optical features of **Re1** were assigned to a change in the excited-state character from MLCT in [ReCl(CO)_3_(Ph-terpy-κ^2^N)] to ILCT in the amine-modified Re(I) system. Owing to the contribution of ^1^ILCT transitions, the lowest absorption band of **Re1** is red-shifted (by ~100 nm) and experiences a significant enhancement in the molar visible absorptivity relative to the poor light absorber [ReCl(CO)_3_(Ph-terpy-κ^2^N]. The triplet excited-state lifetime of **Re1** (^3^ILCT) was found to be 260 times longer compared to [ReCl(CO)_3_(Ph-terpy-κ^2^N] (^3^MLCT). Consistent with the switching of the triplet excited state, a totally different temperature dependence was demonstrated for the emission of the model chromophore and amine-modified Re(I) complex. While the cooling results in a bathochromic emission shift and appearance of the vibronic progression in the spectral band profile of **Re1**, the broad and structureless ^3^MLCT band of the reference chromophore experiences a significant hypsochromic shift [140]. A definitive experimental evidence for a switch from ^3^MLCT excited state in [ReCl(CO)_3_(Ph-terpy-κ^2^N] to ^3^ILCT in **Re1** was provided by Fernández-Terán research group with the use of time-resolved infrared (TRIR) spectroscopy [140] and further confirmed by us on the basis of TA results [141]. Fernández-Terán research group also tested **Re1** as photosensitizer in the photocatalytic hydrogen production, demonstrating a high turnover number (2130 ± 120) and hydrogen production rate (up to 40 nmol s^−1^) upon excitation 443 nm, with the use of triflic acid (0.1 M) as the proton source, triethanolamine (1 M) as a sacrificial electron donor and [Co(dmgH)_2_]_2,_ prepared in situ from Co(BF_4_)_2_·6H_2_O and dmgH_2_ (3.5 mM), as a catalyst. With the use of TRIR spectroscopy, the authors postulated a direct electron transfer from the exciplex between excited Re1* and triethanolamine to the proton reduction catalyst [140]. Contrary to one-photon (1PA) absorption properties, **Re1** and [ReCl(CO)_3_(Ph-terpy-κ^2^N], which were strongly affected by the excited-state character, the measurements of the two-photon (2PA) cross-section of these compounds revealed a minor role of the excited-state nature in controlling their nonlinear optical properties. In this case, an essential effect was attributed to the planarization and conjugation size of the aromatic system. The magnitude of TPA cross-sections of **Re1** and [ReCl(CO)_3_(Ph-terpy-κ^2^N] was 13 and 39 GM, respectively. Variations in structural modifications of the axial ligand of **Re1** may provide additional possibilities to control its linear and nonlinear properties and, thus, their applications [142].

In the very recent years, the complex **Re1** was evaluated for ultrasound-triggered singlet oxygen generation and CO release, which was found to be responsible for its therapeutic effect on normoxic and hypoxic 4T1 cancer cells, confirmed in in vitro and in vivo experiments. Re1 was evidenced to target mitochondria, damaging them by ^1^O_2_ and releasing CO, and thus, inducing cancer cell death. As the CO release is oxygen-independent, it plays a critical role in the treatment of hypoxic tumors, where oxygen-dependent methods (photodynamic, chemotherapy, and sonodynamic therapies) lack their efficacy. Most importantly, comparative analysis of **Re1** with its analogue bearing the electron-withdrawing –NO_2_ substituent confirmed that the appended electron-donating –NMe_2_ group is responsible for stronger luminescence intensity, more efficient singlet oxygen generation by **Re1**, and its better ultrasound-triggered antitumor effect [143].

**Figure 7:**
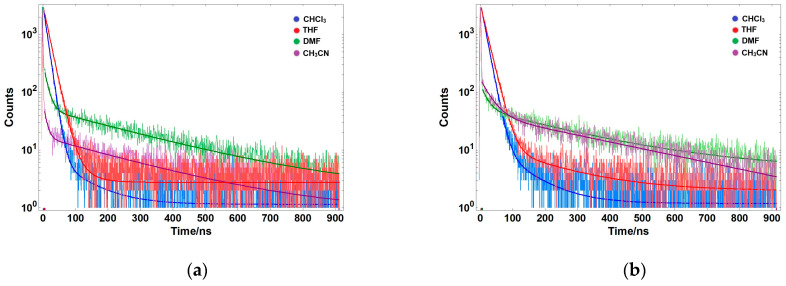
TCSPC decay curves for compounds **Re2** (**a**) and **Re3** (**b**) in solvents of different polarities. The figure was taken from the work [141].

The attachment of other amine groups to Ph-terpy was also found to be an effective way to enhance molar absorptivity and extend excited-state lifetimes of [ReCl(CO)_3_(R-C_6_H_4_-terpy-κ^2^N)] [65,141,144,145,146,147]. The lowest absorption band of these systems displays a bathochromic shift and experiences a large increase in molar extinction coefficients relative to the reference chromophore [ReCl(CO)_3_(Ph-terpy-κ^2^N)]. Their excited-state properties were found to be strongly dependent on the polarity environment, signaling that the triplet excited states ^3^MLCT and ^3^ILCT are close in energy and may vary depending on the solvent polarity. The blue shift of their emission bands in the non-polar CHCl_3_ was found to be indicative of the ^3^MLCT character of the excited state. Electron-rich groups are expected to destabilize the ^3^MLCT excited state, which is manifested in the hypsochromic emission shift. The opposite effect, that is, a bathochromic emission shift, was generally observed in polar acetonitrile, indicating a predominant contribution of the ^3^ILCT excited state in controlling their photobehavior. The ligand-centered character of the emitting triplet state was also supported by a significant increase in the lifetime with an increased environment polarity (Figure 7).

On the basis of fsTA studies for **Re2** and **Re3**, analyzed in comparison to the fsTa spectra of [ReCl(CO)_3_(Ph-terpy-κ^2^N)] and **Re1**, it was postulated that ^3^ILCT excited state is populated via conversion from ^3^MLCT, and this process is affected by the electron-donating ability of the remote amine group and polarity of the environment [141].

The vast majority of [ReCl(CO)_3_(R-C_6_H_4_-terpy-κ^2^N)] with amine-substituted R-C_6_H_4_-terpy ligands, coordinated to the central metal ion in a bidentate coordination mode, were demonstrated to possess the ability for light emission under applied external voltage. The preliminary electroluminescence investigations were performed with the use of laboratory diodes with guest–host configuration ITO/PEDOT:PSS/compound/Al and ITO/PEDOT:PSS/PVK:PBD:compound/Al. The fabricated diodes emitted orange or red light [65,144,145,147].

The conversion of the terpy coordination mode from κ^2^N to κ^3^N was found to lead to dramatic changes in the absorption and emission behavior of **Re12** compared to **Re1** [148]. Typically of Re(I) carbonyls with the *meridionally* coordinated terpy-based ligand, **Re12** shows absorption across the entire visible range of 400–800 nm, being an example of so-called panchromatic systems. Contrary to its analogue with Me_2_NC_6_H_4_-terpy coordinated in a bidentate mode, **Re12** does not show any new absorption in its UV-Vis spectrum, and the position of its lowest energy band experiences a hypsochromic shift relative to the reference chromophore [ReCl(CO)_2_(Ph-terpy-κ^3^N)]. Both in solution at room temperature and glassy matrix at 77 K, **Re12** is non-emissive, which is rationalized by fast non-radiative deactivation owing to significantly red-shifted absorption (in agreement with the energy gap law). TRIR results of **Re12** confirmed unambiguously that its excited-state properties are controlled by ^3^MLCT, formed from the optically populated ^1^MLCT state via intersystem crossing. In contrast to **Re1**, the structural modification of Ph-terpy with the –NMe_2_ moiety does not result in the switch to ^3^ILCT in Re(I) carbonyls with *meridionally* coordinated terpy-based ligand [148]. As demonstrated by theoretical calculations, the complex **Re12** is expected to possess a noticeably larger TPA cross-section relative to **Re1** due to a larger planarity and conjugation [142].

## 8. Square Planar Pt(II) Complexes

**Scheme 9:**
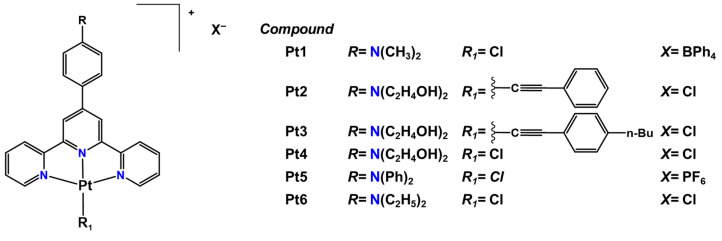
Platinum(II) complexes with amine-substituted R-C_6_H_4_-terpy ligands.

To date, only a few reports on photophysical and electrochemical properties of Pt(II) complexes that include amine-substituted R-C_6_H_4_-terpy ligands have appeared (Scheme 9 and Tables S22–S24).

A comparative analysis of photophysical properties of Pt(II) complexes with amine-substituted R-C_6_H_4_-terpy ligands and those bearing terpy or Ph-terpy supports an essential role of the amine remote group in controlling the photobehavior of [PtX(R-C_6_H_4_-terpy)]^+^. Relative to parent chromophores [PtX(terpy)]^+^ and [PtX(Ph-terpy)]^+^, the lowest energy absorption of [PtX(R-C_6_H_4_-terpy)]^+^, attributed to charge-transfer electronic transitions, displays a red shift and experiences a noticeable increase in the molar absorptivity, in agreement with a significant contribution of ^1^ILCT transitions (Table S22). Conjugating and electron-donating groups attached to the terpy core were evidenced to effectively reduce the rate of thermal decay via the ^3^MC state [149,150,151,152,153,154,155]. While [PtCl(terpy)]Cl is virtually non-luminescent in solution at RT due to the efficient radiationless decay via the higher energy ^3^MC excited state, complexes **Pt1** [156], **Pt2**, **Pt3**, and **Pt4** [155] show the emission originated from configurationally mixed ^3^MLCT/^3^ILCT and ^3^MLCT/^3^ILCT/^3^LLCT excited states, respectively. The involvement of ^3^ILCT in the excited-state photophysics of **Pt1** and **Pt2** is manifested by a bathochromic shift of the emission band and lack of the vibronic progression in room-temperature emission spectra profiles relative to the reference chromophore [PtCl(Ph-terpy)]^+^ (Tables S22 and S23). Worthy of note, the coordination of amine-substituted R-C_6_H_4_-terpys to the alkynylplatinum(II) core {Pt(C≡CR)}^+^ seems to be an effective way to obtain near-infrared emitters. As reported in [155], NIR abilities of **Pt2** may be further improved by attachment of electron-donating groups R to the alkynyl co-ligand, supported by a red shift of **Pt3** (689 nm) in relation to **Pt2** (677 nm).

Pt(II) complexes bearing amine-substituted R-C_6_H_4_-terpy ligands were also demonstrated to be useful for the generation of metallopolymers via the oxidative electropolymerization method [68]. The metallopolymer derived from **Pt5** shows a distinct color transformation from red to blue and possesses promising electrochromic parameters (optical contrast, response times for coloration and bleaching, coloration and bleaching efficiencies, and long-term stability) in view of its potential application in image display [68].

Concerning the shortage of the literature data on [PtX(R-C_6_H_4_-terpy)]^+^ with remote amine substituents, however, more comprehensive research of these systems are strongly needed to provide more precise relationships between photophysical properties of [PtX(R-C_6_H_4_-terpy)]^+^ and molecular modifications of the auxiliary ligand X and electron-donating abilities of the remote amine groups in R-C_6_H_4_-terpy.

## 9. Summary

In this review, we summarized the developments concerning the push–pull systems (D–π–A) based on 2,2′:6′,2″-terpyridine with the acyclic or cyclic amine group attached to the central pyridine ring of the terpy backbone via the phenylene linker, as well as we discussed a role of the intraligand charge transfer (ILCT) excited state in controlling of photophysical properties of Cr(III), Mn(I), Re(I), Fe(II), Ru(II), Os(II), Pt(II), and Zn(II) complexes bearing amine-functionalized R-C_6_H_4_-terpy ligands. The remote redox-active amine groups were evidenced to widely tune electrochemical and photophysical features of R-C_6_H_4_-terpy and their transition metal complexes. Among others, the coordination of amine-substituted R-C_6_H_4_-terpys to transition metal ions was found to lead to substantial enhancement of the visible absorptivity of resulting complexes and bathochromic shift of their emission compared to model chromophores. In many cases, the functionalization of the terpy with the remote amine group was also an efficient way to obtain emitters with prolonged excited-state lifetimes, dual-emissive systems, and materials emitting in the red or near-infrared region.

Functional parameters of these systems were discussed in view of the structure–property relationships and potential applications. Many of the reported compounds were found to be very appealing for optoelectronic applications, photocatalysis, and photochemotherapy. Attention was also devoted to compounds with nonlinear optical response, successfully utilized for cell bioimaging under two-photon confocal microscopy, as well as carbonyl Re(I) and Mn(I) complexes, which emerged as promising candidates for delivering low amounts of CO for therapeutic purposes. Furthermore, the utilization of transition metal complexes bearing amine-substituted R-C_6_H_4_-terpy ligands for the generation of metallopolymers with reversible color change was presented.

Although significant progress has been made in understanding photoinduced processes in amine-substituted R-C_6_H_4_-terpys and their transition metal complexes, a precise control of the functional parameters of these systems as efficient materials for innovative technologies still remains a major challenge, and further more advanced investigations in this field are strongly required. Further structural modifications may provide additional possibilities to control the linear and nonlinear properties of these systems in view of their potential applications. More specifically, based on the current review, it can be clearly stated that d-metal terpyridyl complexes with cyclic amine groups are strongly underexplored. Regarding promising results for Ir(III) and Re(I) systems with this type of ligands, high synthetic efforts towards novel d-metal complexes and their advanced explorations are strongly required. In particular, the introduction of terpy-based ligands with cyclic amine substituents (less electron-donating than –NMe_2_) into the coordination sphere of Cr(III) may result in the formation of Cr(III) complexes with enhanced emission and elongated excited-state lifetimes. In turn, concerning the photophysical results for [IrCl(Ph-py)(morp-C_6_H_4_-terpy-κ^3^N)]PF_6,_ it can be postulated that the functionalization of Ph-terpy with strong electron-donating groups may give rise to the development of novel non-Kasha Ir(III) systems and provide a deeper understanding of the dual-emission phenomena. Regarding the application potential of d-metal complexes with amine-functionalized R-C_6_H_4_-terpy ligands, further investigations should be focused on developing improved (i) NLO optical diagnostic agents for bioimaging at subcellular level, (ii) highly efficient photocatalysts for CO_2_ reduction, (iii) photocatalytic metallodrugs to overcome tumor cell resistance and hypoxia problems, (iv) photoinduced carbon monoxide releasing systems, (v) luminescent metallomesogens, and (vi) electrochromic materials.

## Supplementary Materials

The following supporting information can be downloaded at https://www.mdpi.com/article/10.3390/molecules30112386/s1, Table S1. Selected X-Ray structural parameters of R-C_6_H_4_-terpy1; Table S2. The absorption and emission properties of R-C_6_H_4_-terpys; Table S3. The relevant electrochemical data of R-C_6_H_4_-terpys; Table S4. TPA characteristics of amine-modified R-C_6_H_4_-terpys; Table S5. The absorption and the relevant electrochemical data properties of model Zn(II) complexes; Table S6. The absorption and emission properties of Zn(II) complexes; Table S7. The relevant electrochemical data of Zn(II) complexes; Table S8. The absorption and the relevant electrochemical data properties of model Fe(II) complexes; Table S9. The absorption and the relevant electrochemical data properties of Fe(II) complexes; Table S10. The absorption and the relevant electrochemical data properties of model Ru(II) complexes; Table S11. The absorption and the relevant electrochemical data properties of Ru(II) complexes; Table S12. The absorption and the relevant electrochemical data properties of model Os(II) complexes; Table S13. The absorption, emission and the relevant electrochemical data properties of Os(II) complexes; Table S14. The absorption, emission and the relevant electrochemical data properties of model Cr(III) complexes; Table S15. The absorption, emission and the relevant electrochemical data properties of Cr(III) complexes; Table S16. The absorption and the relevant electrochemical data properties of model Ir(III) complexes; Table S17. The absorption, emission and the relevant electrochemical data properties of Ir(III) complexes; Table S18. The absorption, emission and the relevant electrochemical data properties of Mn(I) complexes; Table S19. The absorption, emission and the relevant electrochemical data properties of model Re(I) complexes; Table S20. The absorption and emission data properties of Re(I) complexes; Table S21. The relevant electrochemical data properties of Re(I) complexes; Table S22. The absorption, emission and the relevant electrochemical data properties of model Pt(II) complexes; Table S23. The absorption, emission data properties of Pt(II) complexes; Table S24. The relevant electrochemical data properties of Pt(II) complexes.
