# Supplementary material for: Effect of Remote Amine Groups on Ground- and Excited-State Properties of Terpyridyl d-Metal Complexes
Source: Molecules. 2025 May 29;30(11):2386. doi: 10.3390/molecules30112386 (PMC12155694; doi:10.3390/molecules30112386)
Supplement: Supplementary file 1 [file molecules-30-02386-s001.zip › molecules-3604478-supplementary.pdf]

SUPPORTING INFORMATION

Effect of remote amine groups on ground- and excited-state properties of terpyridyl d-metal complexes

Anna Kryczka, Joanna Palion-Gazda*, Katarzyna Choroba, Barbara Machura*

Institute of Chemistry, University of Silesia, 9 Szkolna Str., 40-006 Katowice, Poland

Table of contents

Table S1. Selected X-Ray structural parameters of R-C_6H_4-terpy[1]. 2

Table S2. The absorption and emission properties of R-C_6H_4-terpys. 3

Table S3. The relevant electrochemical data of R-C_6H_4-terpys. 8

Table S4. TPA characteristics of amine-modified R-C_6H_4-terpys. 9

Table S5. The absorption and the relevant electrochemical data properties of model Zn(II) complexes. 10

Table S6. The absorption and emission properties of Zn(II) complexes. 10

Table S7. The relevant electrochemical data of Zn(II) complexes. 13

Table S8. The absorption and the relevant electrochemical data properties of model Fe(II) complexes. 13

Table S9. The absorption and the relevant electrochemical data properties of Fe(II) complexes. 14

Table S10. The absorption and the relevant electrochemical data properties of model Ru(II) complexes. 14

Table S11. The absorption and the relevant electrochemical data properties of Ru(II) complexes. 18

Table S12. The absorption and the relevant electrochemical data properties of model Os(II) complexes. 19

Table S13. The absorption, emission and the relevant electrochemical data properties of Os(II) complexes. 20

Table S14. The absorption, emission and the relevant electrochemical data properties of model Cr(III) complexes. 21

Table S15. The absorption, emission and the relevant electrochemical data properties of Cr(III) complexes. 22

Table S16. The absorption and the relevant electrochemical data properties of model Ir(III) complexes. 22

Table S17. The absorption, emission and the relevant electrochemical data properties of Ir(III) complexes. 24

Table S18. The absorption, emission and the relevant electrochemical data properties of Mn(I) complexes. 26

Table S19. The absorption, emission and the relevant electrochemical data properties of model Re(I) complexes. 26

Table S20. The absorption and emission data properties of Re(I) complexes. 28

Table S21. The relevant electrochemical data properties of Re(I) complexes. 29

Table S22. The absorption, emission and the relevant electrochemical data properties of model Pt(II) complexes. 30

Table S23. The absorption, emission data properties of Pt(II) complexes. 31

Table S24. The relevant electrochemical data properties of Pt(II) complexes. 31

References 31

Table S1. Selected X-Ray structural parameters of R-C_6H_4-terpy[1].

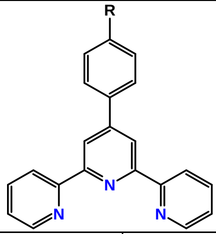

R	Refcode*	C–C$_{Py-Ph}$ bond length [Å]	C–N$_{Ph-N}$ bond length [Å]	Dihedral angle terpy–Ph [°]
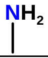	NIQSUB	1.480	1.383	25.91
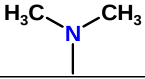	HIVNEG	1.479	1.364	30.09
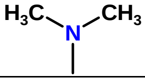	HIVNEG02	1.477	1.375	30.25
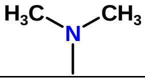	YORSOQ	1.482	1.378	6.93
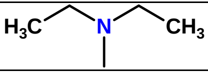	KUFFEY	1.476	1.374	9.34
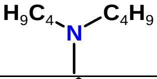	PEHNUP	1.482	1.378	34.46
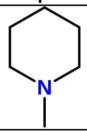	KIXZAW	1.490	1.396	9.08
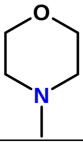	KIXZEA	1.476	1.389	13.30
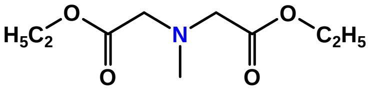	SAWCEB	1.482	1.388	28.06

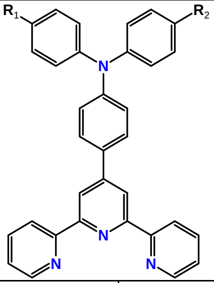

R	Refcode*	C–C$_{Py-Ph}$ bond length [Å]	C–N$_{Ph-N}$ bond length [Å]	Dihedral angle terpy–Ph [°]
**R_1,R_2= H**	EDAKAX	1.483	1.405	35.50
**R_1,R_2= H**	EDAKAX01	1.476	1.426	4.20
**R_1= O–C_2H_5 R_2= NO_2**	ATUSEP	1.486	1.417	44.78
**R_1,R_2= CH_3**	BEPPOE	1.481	1.408	30.43
**R_1,R_2= COO–CH_3**	EDAJUQ	1.497	1.437	38.53
**R_1,R_2= C_2H_4–O–C(O)CH_3**	FOBWIF	1.474	1.381	15.98
**R_1=H R_2=CH-N-OH**	UPURAA	1.475	1.414	32.40
**R_1=H R_2=CHO**	XAJFOH	1.483	1.425	38.60
**R_1=H R_2=CH-C(CN)COOC_2H_5**	XAJFUN	1.482	1.431	38.68
**R_1,R_2= CHO**	ZAPLIN	1.486	1.432	40.00

*Refcode in the Cambridge Structural Database[1]

Table S2. The absorption and emission properties of R-C_6H_4-terpys.

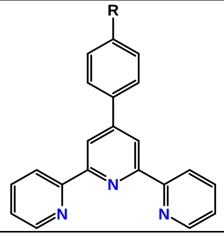

R= H

medium	λ_{abs} [nm]	λ_{exc} [nm]	λ_{PL} [nm]	τ [ns]	φ_{PL}	Reference
DCM	278	278	340	–	0.33	2
Cyclohexane	276	276	338	–	–	
Dipropyl ether	278	278	339	–	–	
EtOH	277	277	340	–	–	
EtOH	282	350	339, 351	1.2	–	3
MeCN	311	311	342, 352sh	–	0.14	4
n-hexane	252, 274, 310	–	337, 349sh	–	–	5
DCM	~255, 250, 325	–	~340, 350	–	–	6
DMF	309	–	–	–	–	7
MeCN	252,275,329	–	–	–	–	
MeOH	–	–	–	0.32	0.004	8
DCM	~250,275,315	–	–	–	–	9
DCM	~325, 281, 250	276	340	–	0.18	10
solid	–	340	393	–	–	
solid	245, 289	–	395	–	–	11
DCM	277, 309sh	–	340, 352	–	0.16	12
EtOH	277, 310sh	–	338, 351	–	0.10	
MeCN	223, 251, 275, 314	–	–	–	–	13
Solid	–	316	374	2.73	0.18	14
Solid	–	280	365	–	–	15
EtOH	–	289	338, 344	–	–	16
Solid		290	376	3.05	0.182	17
Solid 77 K	–	290/350	362/525	3.38/0.83	–	
2-MeTHF	–	290	354	1.82	0.104	
2-MeTHF 77 K	–	290/331	350/471	3.17/1.70	–	
DMSO	280	–	–	–	–	18
$CHCl_3$	~282, 305	–	–	–	–	19
MeOH	250, 275, 315	–	–	–	–	20
MeCN	277.5, 311.5	–	–	–	–	21

R= 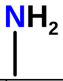

medium	λ_{abs} [nm]	λ_{exc} [nm]	λ_{PL} [nm]	τ [ns]	φ_{PL}	Reference
DMSO	291, 337	366	531	–	–	22
DCM	288, 325	–	450	–	0.13	2,23
MeOH	204, 234, 286, 314, 326, 408	–	–	–	–	24
Cyclohexane	286	–	367	–	–	2
Dipropyl ether	290, 328	–	420	–	–	
EtOH	288, 326	–	506	–	–	

R= 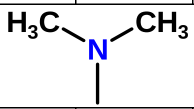

medium	λ_{abs} [nm]	λ_{exc} [nm]	λ_{PL} [nm]	τ [ns]	φ_{PL}	Reference
Hexane	254, 280sh, 290, 332	–	–	–	–	25
$CHCl_3$	253sh, 280sh, 291, 347	248, 299, 349	442	3.16	0.19	
MeCN	252sh, 278sh, 289, 346	346	545	4.35	0.09	
MeOH	249sh, 280sh, 289, 349	–	–	–	–	
Solid	–	499	551	3.47	0.36	

Film	312, 390	364	457	–	–	
Blend[a]	–	310	417	–	–	
Blend[b]	–	310	436	–	–	
Hexane	~290, 330	380	~400	–	–	26
Toluene	~295, 345		~425	–	–	
THF	~290, 345		~470	–	–	
MeCN	~280, 340		~525	–	–	
MeCN	342	342	515	–	0.15	4
EtOH	291, 348	350	531	2.60	0.01	3
Cyclohexane	337	350	369, 385sh	1.70	0.31	
THF	346	340	469	5.20	0.22	
DCM	348	340	470	5.20	0.27	
DCM	231, 293, 349	397	470	5.24	0.28	27
MeCN	223, 292, 352, 432	346	545	4.35	0.09	
Solid	–	499	551	3.47	0.36	
EtOH	347	379	531		0.06	28
Toluene	~330, 400	–	~530	–	–	
H_2O	~310, 325, 400	–	~510	–	–	
DMSO	~315, 335, 425	–	~605	–	–	
Film	360	–	510	–	–	
Cyclohexane	337	350	369	1.7	0.31	29
THF	346		469	5.2	0.22	
DCM	348		470	5.2	0.27	
EtOH	348		531	2.6	0.01	
DCM	291, 348	–	469	–	0.23	2
Cyclohexane	290, 334	–	383	–	–	
Dipropyl ether	290, 337	–	417	–	–	
EtOH	289, 348	–	511	–	–	
$MeCN:H_2O$ (4:1 v:v)	234, 289, 345	–	–	–	–	30

R= 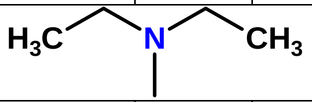

medium	λ_{abs} [nm]	λ_{exc} [nm]	λ_{PL} [nm]	τ [ns]	φ_{PL}	Reference
Benzene	~295, 359	359	441	–	–	30
THF	~290, 356	356	471	–	–	
Ethyl acetate	~290, 356	356	464	–	–	
EtOH	~290, 358	358	513	–	–	
MeCN	~290, 357	357	515	–	–	
$DMSO:H_2O$ (1:1 v:v)	~290, 371	371	524	–	–	
MeCN	278, 290, 356	–	–	–	–	31

R= 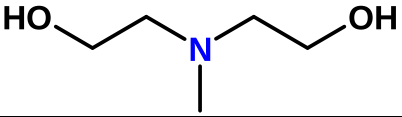

medium	λ_{abs} [nm]	λ_{exc} [nm]	λ_{PL} [nm]	τ [ns]	φ_{PL}	Reference
H_2O	234, 290, 350	–	525	–	–	32
Benzene	–	–	415	–	–	
Ethyl acetate	–	–	458	–	–	
Acetone	–	–	486	–	–	
DMF	–	–	500	–	–	
EtOH	–	–	518	–	–	
$EtOH:H_2O$ (2:1 v:v)	205, 234, 289, 351	–	–	–	–	33
PBS:DMSO (95:5 v:v)	234, 290, 352	–	–	–	–	34
DMF	–	361	506	–	–	
H_2O	~290, 365	–	526	–	–	35

R= 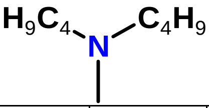

medium	λ_{abs} [nm]	λ_{exc} [nm]	λ_{PL} [nm]	τ [ns]	φ_{PL}	Reference
MeOH	~290, 360	–	–	–	–	36
DCM	~300, 360	–	–	–	–	
DMSO	~310, 370	–	–	–	–	
H_2O	~290, 355	–	–	–	–	
Ethyl acetate	~290, 380	–	–	–	–	

Toluene	~280, 365	–	~430	–	–	37
Ethyl acetate	~280, 355	–	~460	–	–	
$CHCl_3$	~280, 365	–	~450	–	–	
THF	~280, 360	–	~450	–	–	
Acetone	~360	–	~490	–	–	
MeOH	~310, 365	–	–	–	–	
H_2O:DMSO (9:1 v:v)	~305, 365	~365	–	–	–	
$CHCl_3$	360	–	–	–	–	38
THF	358	–	453	–	0.39	39
$CHCl_3$	360	–	449	–	–	40
$CHCl_3$	360	–	449	–	–	41

R=
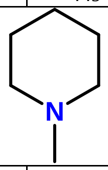

medium	λ_{abs} [nm]	λ_{exc} [nm]	λ_{PL} [nm]	τ [ns]	φ_{PL}	Reference
Hexane	233, 252, 326	–	–	–	–	25
$CHCl_3$	248sh, 290, 335	252, 308, 336	432	3.35	0.30	
MeCN	233, 288, 335	252, 275, 295, 352	516	5.53	0.16	
MeOH	232, 286, 333	–	–	–	–	
Solid	–	266, 305, 350, 409, 490	563	2.98	0.16	
Film	347	347	442	–	–	
Film	–	310	418	–	–	
blend[a]	–	310	431	–	–	

R=
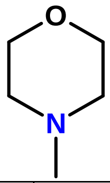

medium	λ_{abs} [nm]	λ_{exc} [nm]	λ_{PL} [nm]	τ [ns]	φ_{PL}	Reference
Hexane	230, 291, 318	–	–	–	–	25
$CHCl_3$	288, 298sh, 323	254, 298, 328	432	2.69	0.33	
MeCN	230, 288, 324	253, 296, 327	516	5.86	0.28	
MeOH	232, 289, 324	–	–	–	–	
Solid	–	268, 309, 248, 369, 452	499	4.68	0.28	
Film	332	308	425	–	–	
Film	–	363, 466	410	–	–	
blend[a]	–	310	420	–	–	

R=
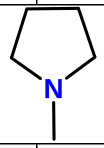

medium	λ_{abs} [nm]	λ_{exc} [nm]	λ_{PL} [nm]	τ [ns]	φ_{PL}	Reference
MeCN	195, 233, 287, 335	250, 277, 295, 336	536	5.77	0.24	42
$CHCl_3$	290, 332	260, 280, 299, 336	462	3.53	0.44	
Solid	–	266, 310, 347, 395, 467	536	2.35	0.14	
Film	365	330	536	–	0.02	
Blend	–	330	414	–	0.06	

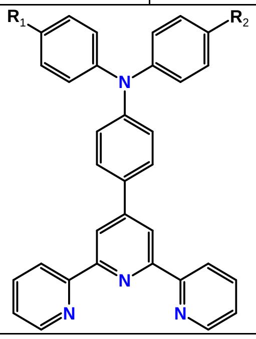

R_1, R_2= H

medium	λ_{abs} [nm]	λ_{exc} [nm]	λ_{PL} [nm]	τ [ns]	φ_{PL}	Reference
MeCN	~295, 360	–	–	–	–	43
Solid	–	350	517	–	0.18	44
Solid (Crystalline)	–		520	–	0.28	
MeCN	356		489	–	0.08	
THF	358		451	–	0.16	
Toluene	363		426	–	0.30	
$CHCl_3$	364		451	–	0.22	
EtOH	361		491	–	0.05	
Ethyl acetate	356		452	–	0.30	
DMF	359		484	–	0.18	
Hexane	241, 288, 355	–	–	–	–	25
$CHCl_3$	244, 291, 362	254, 281, 299, 361	454	3.31	0.86	
MeCN	241, 283, 356	249, 280, 294, 355	499	4.95	0.80	
MeOH	241, 288, 360	–	–	–	–	
Solid	–	265, 304, 350, 410, 474	533	3.81	0.02	
Film	308, 367	360	426, 480	–	–	
Film	–	363, 466	420	–	–	
Blend[a]	–	310	436	–	–	
DCM	–	~290, 365	473	4.17	–	45
$CHCl_3$	–	~295, 360	454	3.31	–	
MeCN	–	~290, 355	498	4.78	–	
DMSO	–	~295, 365	504	5.90	–	
DMF	–	~295, 360	492	5.09	–	46
Hexane	360	–	395	1.1	0.51	
Cyclohexane	360	–	395	1.2	0.53	
1,4-Dioxane	360	–	435	3.6	0.77	
Toluene	365	–	420	2.1	0.47	
Diethyl ether	360	–	425	2.7	0.88	
Anisole	365	–	450	3.1	0.61	
$CHCl_3$	360	–	455	3.6	0.84	
Chlorobenzene	360	–	445	3.4	0.67	
Ethyl acetate	360	–	455	3.2	0.75	
THF	360	–	455	3.2	0.78	
DCM	360	–	470	4.7	0.82	
1,2-Dichlorobenzene	365	–	455	3.8	0.50	
1,2-Dichloroethane	360	–	470	4.4	0.89	
t-Butanol	360	–	470	4.9	0.87	
Benzyl alcohol	370	–	505	2.5	0.11	
i-Butanol	360	–	490	5.2	0.81	
3-Pentanone	360	–	470	4.5	0.21	
n-Butanol	360	–	490	4.6	0.70	
2-Butanol	365	–	480	5.3	0.97	
i-Propanol	360	–	490	5.8	0.90	
n-Propanol	360	–	495	4.2	0.67	
Acetone	360	–	480	4.2	0.68	
Benzonitrile	365	–	480	4.1	0.48	
MeOH	360	–	535	0.57	0.40	
MeCN	360	–	500	4.9	0.65	
DMF	360	–	495	5.1	0.78	
DMSO	365	–	500	6.4	0.81	
EtOH	431	336	509	–	0.14	28
MeCN	~295, 360	–	–	–	–	47
DCM	361	–	–	–	–	48
Benzene	288, 360	360	430	1.98	0.65	49
DCM	287, 358	358	~460	2.82	0.48	
Ethyl acetate	286, 356	356	~450	3.23	0.60	
THF	288, 357	357	~455	2.70	0.70	
MeCN	286, 351	351	~495	4.83	0.29	
EtOH	285, 358	358	~500	2.59	0.17	
DMF	287, 359	359	485	5.19	0.46	
Cyclohexane	356	350	392, 414sh	1.2	0.36	29
THF	360	–	453	3.4	0.51	
DCM	361	–	472	4.0	0.58	
EtOH	362	–	513	2.5	0.25	
EtOH	290, 362	350	513	2.5	0.25	3

Cyclohexane	356	–	392, 414sh	1.2	0.36	
THF	360	–	453	3.4	0.51	
DCM	361	–	472	4.0	0.58	
DCM	–	–	472	–	0.58	50
DCM	288, 356	–	–	–	–	51
MeOH	361	–	–	–	–	52
MeCN	–	–	498	6.5	0.15	
MeCN	~250, 290, 360	–	–	–	–	53
Benzene	289, 359	–	430	2.37	0.56	54
DCM	287, 357	–	465	3.97	0.52	
THF	286, 355	–	449	3.43	0.63	
Ethyl acetate	286, 354	–	454	3.27	0.54	
MeCN	283, 353	–	492	4.89	0.22	
DMF	286, 357	–	487	5.25	0.40	
Benzene	285, ~360	–	~430	–	–	55
n-Hexane	354	–	393	–	–	56
DCM	360	–	476	–	–	
MeCN	357	–	489	–	–	
EtOH	362	–	505	–	–	
MeOH	359	–	524	–	–	

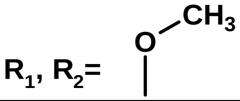

medium	λ_{abs} [nm]	λ_{exc} [nm]	λ_{PL} [nm]	τ [ns]	φ_{PL}	Reference
MeCN	288, 365	–	–	–	–	57
MeCN	~240, 290, 360	–	–	–	–	53

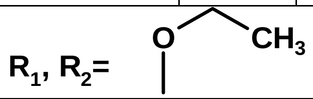

medium	λ_{abs} [nm]	λ_{exc} [nm]	λ_{PL} [nm]	τ [ns]	φ_{PL}	Reference
Ethyl acetate	~285, 365	–	–	–	–	58
EtOH	~285, 365	–	–	–	–	
DMSO	~285, 360	–	~485	–	–	
DMF	~285, 355	–	–	–	–	
Benzene	~270, 321	–	–	–	–	59
Solid	~475	–	~490	–	–	
THF	~265, 335	–	–	–	–	60
EtOH	~265, 340	–	–	–	–	
MeCN	~260, 315	–	–	–	–	
DMF	~265, 325	–	–	–	–	
DMSO	~265, 345	–	–	–	–	
Benzene	278, 372	–	481	4.11	0.69	55
DCM	290, 368	–	525	5.49	0.23	
Ethyl acetate	281, 366	–	504	4.58	0.37	
EtOH	286, 670	–	523	0.74	0.006	
N,N-dimethylformamide	286, 369	–	526	1.74	0.048	

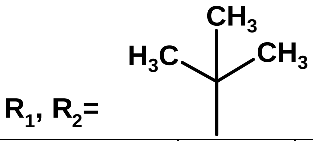

medium	λ_{abs} [nm]	λ_{exc} [nm]	λ_{PL} [nm]	τ [ns]	φ_{PL}	Reference
DCM	369	390	495	–	–	61
film	–	390	449	5.00	0.67	
$CHCl_3$	292, 371	254, 297, 372	489	4.30	0.84	62
MeCN	287, 362	295, 363	525	5.40	0.63	
Solid	–	266, 331, 420	445	1.33	0.21	
Film	303, 372	278, 370	448	–	–	
n-Hexane	291, 364	365	407	1.53	0.48	5
n-Hexane 77 K	–	403	438	1.48	–	
Cyclohexane	293, 366	365	408	1.55	0.54	
Cyclohexane 77 K	–	385	418	1.94	–	
Toluene	293, 369	368	435	2.55	0.64	
Toluene 77 K	–	396	422	2.00	–	
$CHCl_3$	294, 371	371	487	4.30	0.84	
$CHCl_3$ 77 K	–	394	442	3.05	–	
Ethyl acetate	291, 363	364	468	3.57	0.70	
Ethyl acetate 77 K	–	387	424	2.44	–	

medium	λ_{abs} [nm]	λ_{exc} [nm]	λ_{PL} [nm]	τ [ns]	φ_{PL}	Reference
THF	291, 364	365	467	3.81	0.75	
THF 77 K	–	397	428	2.12	–	
DCM	292, 369	367	497	4.83	0.79	
DCM 77 K	–	395	439	2.75	–	
DMF	291, 367	365	518	5.87	0.77	
DMF 77 K	–	380	439	2.90	–	
DMSO	292, 372	372	528	6.56	0.83	
DMSO 77 K	–	386	441, 516sh	2.52	–	
MeCN	289, 364	363	527	5.53	0.63	
MeCN 77 K	–	383	463	3.52	–	
MeOH	290, 369	371	557	–	0.02	
EtOH:MeOH (4:1 v:v) 77 K	–	394	438	2.95 0.75	–	
BuCN	–	367	508	4.89	–	
BuCN 77 K	–	387	429	2.33	–	
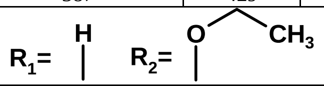						
**medium**	**λ_{abs} [nm]**	**λ_{exc} [nm]**	**λ_{PL} [nm]**	**τ [ns]**	**φ_{PL}**	**Reference**
DMF	–	–	~470	–	–	63

Blend[a] consists of 2 wt. % of compound in a mixture of PVK:PBD. Blend[b] consists of 15 wt % of compound in a mixture of PVK:PBD.

Table S3. The relevant electrochemical data of R-C_6H_4-terpys.

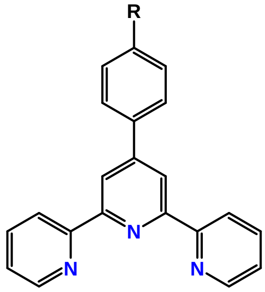

**R= H**						
**medium**	**$E_{red,peak}$ [V] (ΔE [mV])/ $E_{red,onset}$ [V] or $E_{1/2red}$[V]**	**$E_{ox, peak}$ [V]/$E_{ox, onset}$ [V] /$E_{1/2ox}$ [V]**	**IP[a] [eV]**	**EA[b] [eV]**	**E_g[c] [eV]**	**Reference**
DMSO 0.1 M $Bu_4NCF_3SO_3$	–2.36 (95)	–	–	–	–	64
DMF 0.1 M Bu_4NClO_4	-1.48_{irr}, –1.93	–	–	–	–	20
MeCN 0.1 M Bu_4NPF_6	–2.51(350)/–2.37	1.66/1.45	–6.55	–2.75	3.80	42
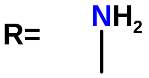						
MeCN 0.1 M Bu_4NPF_6	–	0.88_{irr}	–	–	–	24
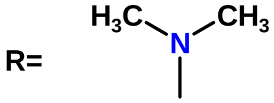						
DCM 0.1 M Bu_4NPF_6	–2.26	0.36, 0.60	–5.46	–2.84	2.62	25
DCM 0.2 M Bu_4NPF_6	–2.26	0.36, 0.60	–5.46	–2.84	2.61	27
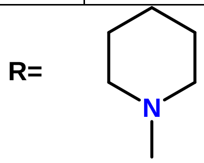						
DCM 0.1 M Bu_4NPF_6	–2.26	0.40, 0.58	–5.50	–2.84	2.66	25
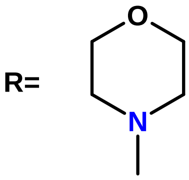						

DCM 0.1 M Bu_4NPF_6	–2.35	0.48, 0.68	–5.58	–2.75	2.83	25
**medium**	**$E_{red,peak}$ [V] (ΔE [mV])/ $E_{red,onset}$ [V] or $E_{1/2red}$[V]**	**$E_{ox, peak}$ [V]/$E_{ox, onset}$ [V] /$E_{1/2ox}$ [V]**	**IP[a] [eV]**	**EA[b] [eV]**	**E_g[c] [eV]**	**Reference**
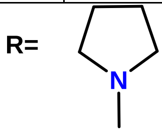						
MeCN 0.1 M Bu_4NPF_6	–2.53 (112) /–2.34	0.45 (92), 0.55, 0.69/0.32	–5.42	–2.76	2.66	42
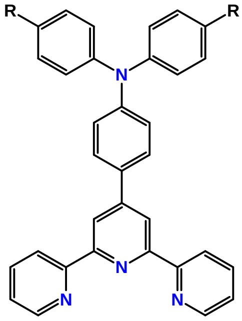						
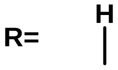						
DCM 0.1 M Bu_4NPF_6	–2.36	0.49	–5.59	–2.74	2.85	25
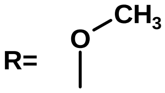						
MeCN 0.1 M Bu_4NClO_4	–	0.81, 1.25_{irr}	–	–	–	57
MeCN 0.1 M Bu_4NClO_4	–	0.81 (80)	–	–	–	65
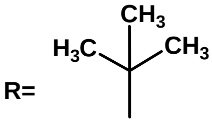						
DCM 0.1 M Bu_4NBF_4	–	0.56, 0.75, 1.07	–5.66	–2.74	–	62

[a] IP = −5.1–$E_{ox(onset)}$, [b] EA = −5.1-$E_{red(onset)}$, [c] Eg = $E_{ox(onset)}$ − $E_{red(onset)}$.

Table S4. TPA characteristics of amine-modified R-C_6H_4-terpys.

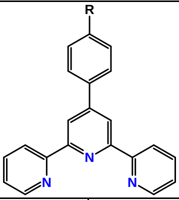				
**R**	**Solvent**	**TPA cross-section δ_{TPA} [GM]**	**TPA emission λ_{max} [nm]**	**Reference**
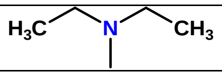	DMSO	261*	700	66
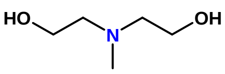	DMSO	–	528**	35
			506	34
	H2O:MeCN (4:1 v:v)	515*	507	32
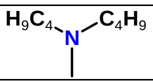	DMSO	508*	780	37
	DMF	87***	720	39

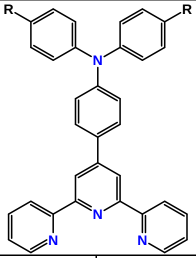

R	Solvent	TPA cross-section δ_{TPA} [GM]	TPA emission λ_{max} [nm]	Reference
H	DMF	434**	~720	54
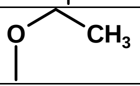	DMSO	389*	760	58
	Benzene	1060*	750	55
–COOCH3	DMF	179**	~840	54

*Open-aperture Z-scan technique
**Two-photon excited fluorescence (TPEF) method
***TPA induced photoluminescence (TPA-PL) method

Table S5. The absorption and the relevant electrochemical data properties of model Zn(II) complexes.

medium		λ_{abs} [nm]	λ_{exc} [nm]	λ_{PL} [nm]	τ [ns]	φ_{PL}	$E_{red,peak}$ [V] (ΔE [mV])/ $E_{red,onset}$ [V]/ $E_{1/2red}$[V]	$E_{ox,peak}$ [V]/$E_{ox,onset}$ [V] /$E_{1/2ox}$ [V]	Reference
**Zn(Ph-terpy)(OAc)$_2$**									
H_2O			–	~475	–	–	–	–	67
**Zn(Ph-terpy)Cl$_2$**									
DMF:H_2O (5:95 v:v)		~225, 280, 335	–	–		–	–	–	68
**[Zn(Ph-terpy)$_2$]X$_2$**									
PF_6	MeCN	285	300	350	–	–	–	–	10
			350	–	–	0.6046	–	–	
	Solid	–	352	468	–	–	–	–	
p-OSO_2PhCH_3	DMF	311	310	370, 510	–	–	–	–	69
	Solid	–	300	384 ,522	–	–	–	–	
NO_3	DMF	315	310	374	–	–	–	–	
	Solid	–	300	381, 544	–	–	–	–	
SO_3CF_3	DMF	306,330	310	390, 517	–	–	–	–	
	Solid	–	300	383, 502	–	–	–	–	
SbF_6	DMF	330	310	370, 510	–	–	–	–	
	Solid	–	300	375	–	–	–	–	
ClO_4	DMF 0.1 M TBAP	–	–	–	–	–	$E_{1/2}$ = –1.20 (80), –1.32 (95)	–	70
	DMF:H_2O (1:1 v:v)	269sh, 280sh, 286, 323, 336sh, 406, 531	–	–	–	–	–	–	
–	MeCN	337	337	362	2.6	0.52	–	–	71
**[Zn(terpy)$_2$]X$_2$**									
–	Phosphate buffer	265, 275, 282, 320, 333	–	–	–	–	–	–	72
NO_3	DMF	–	–	–	–	–	–1.55	–	73
	MeOH	~235, 270, 280, 285, 320, 335	–	~370	–	–	–	–	
OAc	MeCN	~275, 285, 315, 335	–	353	–	0.65	–	–	74
PF_6	BuCN 77 K	–	–	~440, 455, 470, 485	–	–	–	–	75

Table S6. The absorption and emission properties of Zn(II) complexes.

medium	λ_{abs} [nm]	λ_{exc} [nm]	λ_{PL} [nm]	τ [ns]	φ_{PL}	Reference
**Zn1**						
Ethyl acetate	408	408	586	2.53	0.22	76
H_2O:MeCN (1:9 v:v)	~425	–	–	–	–	
**Zn2**						
Ethyl acetate	422	422	589	1.61	0.19	76
H_2O:MeCN (1:9 v:v)	~425					
**Zn3**						

Ethyl acetate	431	431	590	1.38	0.10	76
H_2O:MeCN (1:9 v:v)	~425	–	–	–	–	
**medium**	**λ_{abs} [nm]**	**λ_{exc} [nm]**	**λ_{PL} [nm]**	**τ [ns]**	**φ_{PL}**	**Reference**
**Zn4**						
EtOH	281, 323, 410	–	–	–	–	58
Ethyl acetate	~280, 325, 415	–	–	–	–	
DMSO	~285, 330, 425	–	~545	–	–	
DMF	~285, 325, 415	–	–	–	–	
**Zn5**						
EtOH	281, 322, 410	–	–	–	–	58
Ethyl acetate	~280, 325, 410	–	–	–	–	
DMSO	~280, 330, 410	–	~570	–	–	
DMF	~280, 330, 420	–	–	–	–	
**Zn6**						
EtOH	281, 322, 415	–	–	–	–	58
Ethyl acetate	~280, 325, 425	–	–	–	–	
DMSO	~280, 335, 375	–	~570	–	–	
DMF	~280, 330, 375	–	–	–	–	
**Zn7**						
Solid	–	500	555	2.2	0.13	77
1,4-dioxane	395	–	500	5.0	0.86	46
Toluene	395	–	485	4.0	0.57	
Anisole	395	–	530	5.2	0.82	
$CHCl_3$	410	–	525	5.5	0.88	
Chlorobenzene	415	–	450	5.3	0.79	
Ethyl acetate	410	–	545	5.4	0.92	
DCM	405	–	570	6.6	0.71	
1,2-dichlorobenzene	415	–	550	6.2	0.81	
1,2-dichloroethane	420	–	570	5.7	0.58	
t-Butanol	400	–	550	5.5	0.50	
Benzyl alcohol	420	–	590	4.7	0.41	
i-Butnol	410	–	565	3.8	0.28	
3-pentanone	400	–	575	3.6	0.26	
n-Butanol	415	–	570	3.2	0.21	
2-Butanol	405	–	560	4.6	0.44	
i-Propanol	410	–	570	3.2	0.25	
n-Propanol	410	–	575	1.8	0.15	
Acetone	405	–	590	1.9	0.00	
Benzonitrile	420	–	605	3.7	0.35	
MeOH	420	–	660	0.33	0.01	
MeCN	410	–	635	1.0	0.02	
DMF	410	–	605	0.94	0.00	
DMSO	415	–	635	0.94	0.00	
**Zn8**						
Solid	–	445	520	3.3	0.43	77
**Zn9**						
H_2O	~285, 385	–	–	–	–	78
solid	–	365	~575	–	–	
**Zn10**						
H_2O	~290, 390	–	–	–	–	78
solid	–	365	~625	–	–	
**Zn11**						
H_2O	240, 288, 316, 330, 441	–	588	–	–	32
Acetone	–	–	502	–	–	
MeCN	–	–	512	–	–	
DMF	–	–	518	–	–	
EtOH	–	–	574	–	–	
**Zn12**						
DCM	284, 302, 407	–	549	1.85	0.26	45
Solid	–	–	560	–	0.16	
$CHCl_3$	–	–	522	–	–	
MeCN	–	–	495	–	–	
DMSO	–	–	497	–	–	

DMF	–	–	491	–	–	
**Zn13**						
DCM	284, 321, 334, 457	–	576	1.67	0.19	45
Solid	–	–	604	–	0.19	
$CHCl_3$	–	–	521	–	–	
MeCN	–	–	497	–	–	
DMSO	–	–	497	–	–	
DMF	–	–	491	–	–	
DMF	325, 450	450	590	2.4	0.06	79
Acetone	~430	–	–	–	–	46
**medium**	**λ_{abs} [nm]**	**λ_{exc} [nm]**	**λ_{PL} [nm]**	**τ [ns]**	**φ_{PL}**	**Reference**
**Zn14**						
DCM	284, 321, 333, 456	–	594	1.31	0.16	45
Solid	–	–	601	–	0.21	
$CHCl_3$	–	–	528	–	–	
MeCN	–	–	498	–	–	
DMSO	–	–	496	–	–	
DMF	–	–	491	–	–	
**Zn15**						
DCM	284, 319, 332, 458	–	622	1.19	0.13	45
Solid	–	–	606	–	0.08	
$CHCl_3$	286, 320, 335, 453	–	540	3.82	0.34	
MeCN	285, 316, 329, 424	–	498	5.24	0.06	
DMSO	285, 329, 364, 424	–	497	6.25	0.43	
DMF	285, 334, 364, 422	–	489	5.45	0.48	
MeCN	~240, 275, 320, 407	–	–	–	–	53
**Zn16**						
MeCN	424	–	625	0.1	0.0007	71
**Zn17**						
H_2O	~280, 380, 450	–	–	–	–	78
**Zn18**						
DCM	~280, 340, 440	–	~435	–	–	63
THF	~280, 340, 420	–	~435	–	–	
MeCN	~280, 335, 415	–	–	–	–	
DMF	~280, 335, 370	–	~460	–	–	
Ethyl acetate	–	–	~435	–	–	
**Zn19**						
EtOH	281, 315, 445	–	–	–	–	58
Ethyl acetate	~280, 320, 425	–	–	–	–	
DMSO	~280, 345, 425	–	~540	–	–	
DMF	~280, 345, 425	–	–	–	–	
**Zn20**						
Benzene	415	415	453	–	–	66
THF	416	416	475	–	–	
Acetic ether	410	410	465	–	–	
EtOH	449	449	572	–	–	
MeCN	439	439	587	–	–	
DMSO:H_2O (1:1 v:v)	442	442	576	–	–	
**Zn21**						
$CHCl_3$	325	438	488	–	–	80
EtOH:$CHCl_3$ (1:1 v:v)	415	–	551	–	–	
**Zn22**						
DCM	255, 284, 309, 322, 406	410	488	4.2	0.9500	81
Cyclohexane	–	430	542	–	0.2020	
**Zn23**						
H_2O	285, 325, 405	365	~540	–	0.0001	78
Solid	–	365	558	–	0.0145	
**Zn24**						
DCM	~285, 315, 330, 415	–	–	–	–	81
Cyclohexane	–	430	542	–	0.2020	

Table S7. The relevant electrochemical data of Zn(II) complexes.

medium	$E_{red, onset}$	$E_{ox, onset}$	IP[a] [eV]	EA[b] [eV]	E_g[c] [eV]	Reference
**Zn12**						45
DCM 0.1 M Bu_4NPF_6	-0.96_{irr}	0.72	–5.8	–4.04	1.68	
**Zn13**						
DCM 0.1 M Bu_4NPF_6	-0.87_{irr}	0.72	–5.82	–4.23	1.59	
**Zn14**						
DCM 0.1 M Bu_4NPF_6	-0.75_{irr}	0.72	–5.82	–4.35	1.47	
**Zn15**						
DCM 0.1 M Bu_4NPF_6	-0.61_{irr}	0.72	–5.82	–4.49	1.33	

[a] $IP = -5.1 - E_{ox(onset)}$, [b] $EA = -5.1 - E_{red(onset)}$, [c] $Eg = E_{ox(onset)} - E_{red(onset)}$.

Table S8. The absorption and the relevant electrochemical data properties of model Fe(II) complexes.

X^-	medium	λ_{abs} [nm]	$E_{red,peak}$ [V] (ΔE [mV])/ $E_{red,onset}$ [V]/ $E_{1/2red}$[V]	$E_{ox,peak}$ [V]/$E_{ox,onset}$ [V] /$E_{1/2ox}$ [V]	Reference
**$[Fe(Ph\text{-}terpy)_2]X_2$**					
BF_4	MeOH	572	–	–	82
	DMF 0.1 M Bu_4NClO_4	–	$E_{1/2}$ = –1.64 (80), –1.73 (80)	$E_{1/2}$ = 0.60	
ClO_4	6% DMF:tris-HCl buffer (pH=7.2)	370,566	–	–	83
	MeCN 0.1 M Bu_4NClO_4	–	-1.20^{qr}, -1.25^{qr}	1.0	
	MeCN	288, 325, 520	–	–	21
PF_6	MeCN 0.1 M Bu_4NPF_6	–	–1.62, –1.73, –2.34	0.69	84
	MeCN	284, 322, 565	–	–	
NO_3	DMF:H_2O (5:95 v:v)	~215, 285, 330	–	–	68
OAc	DMF	284, 318sh, 568	–	–	85
	DMF 0.1 M $TBAPF_6$	–	–	1.05	
Cl	DMF 0.1 M Bu_4NPF_6	–	–1.24 (60), –1,35 (60), –2.01 (110)	–	86
	DMF	~330, 380, 575	–	–	
**$[Fe(terpy)_2]X_2$**					
Cl	MeOH	320, 363, 484, 551, 607	–	–	87
	MeCN	~280, 290sh, 320, 370, 570	–	–	88
PF_6	MeCN	552	–	–	89
	DCM 0.1 M Bu_4NPF_6	–	–	1.36	
	MeCN	~280, 290sh, 325, 360, 551	–	–	90
	MeCN 0.1 M Bu_4NPF_6	–	–1.64, –1.80	0.72	
	MeCN	~360, 560	–	–	91
	MeCN	365, 504, 552, 609	–	–	92
	DCM	~3275, 290sh, 325, 590	–	–	
	MeCN 292 K	~360, 500, 560, 640	–	–	93
	MeCN 0.1 M Bu_4NPF_6	–	$E_{1/2}$= –1.64 (63)	$E_{1/2}$= 0.72	
	MeCN	552	–	–	90
BPh_4		~280, 325, 375, 550	–	–	
PF_6	MeCN 0.1 M Bu_4NPF_6	–	$E_{1/2}$ = –1.64, –1.80	$E_{1/2}$ = 0.72	
	MeCN 0.1 M Bu_4NPF_6	–	$E_{1/2}$ = –1.26	$E_{1/2}$ = 1.10	94
BF_4	MeCN 0.1 M Bu_4NPF_6	–	$E_{1/2}$= –1.66, –1.81, –1.99	$E_{1/2}$ = 0.71	95
	MeCN	220sh, 273, 280, 319, 504sh, 552, 590sh	–	–	
	MeCN 0.1 M Bu_4NPF_6	–	$E_{1/2}$ = –1.26	$E_{1/2}$ = – 1.10	96
PF_6	MeCN	552	–	–	97
	MeCN 0.1 M Bu_4NPF_6	–	–1.57, –1.72, -2.39^{irr}	0.69	
	MeCN	~350, 500, 550, 620	–	–	98
	MeCN 0.1 M Bu_4NPF_6	–	$E_{1/2}$=-1.68	$E_{1/2}$=0.71	99
	MeCN	~370, 480, 550, 610	–	–	
	MeCN 0.1 M Bu_4NBF_4	–	–1.64, –1.82	0.74	100
	MeOH	~270, 280sh, 320, 370, 480, 560	–	–	
	MeCN 0.1 M TBAP	–	–1.13 (70), –1.27 (60), –1.94 (70)	1.10	101
	MeCN 0.1 M Bu_4NPF_6	–	$E_{1/2}$ = –1.27, 1.43	$E_{1/2}$ = 1.09	102
	MeCN	319, 552	–	–	
	DMSO	~280, 325, 380, 400, 550	–	–	103
		–	–1.47, –1.62	0.805	
	MeCN 0.1 M Bu_4NPF_6	–	–1.65, –1.85	0.77	104
	MeCN	551	–	–	
	MeCN 0.1 M Bu_4NPF_6	–	–	$E_{1/2}$ = 1.13	105

	MeCN	~280, 325, 370, 560	–	–	106
	MeCN 0.1 M Et_4NClO_4	–	–1.13 (70), –1.27 (60), –1.94 (70)	1.10	107
	MeCN 0.1 M Bu_4NPF_6	–	–1.64, –1.81	0.72	108
ClO_4	MeCN 0.1 M Et_4NBF_4	–	$E_{1/2}$ = –1.21 (60), –1.36 (67)	$E_{1/2}$ = 1.13	109
	MeCN 0.25 M Et_4NClO_4	–	–1.652, –1.790	0.702	110
Cl	MeCN 0.1 M $LiClO_4$	–	–	1.0±0.02[r]	111
	MeCN	~280sh, 290, 330, 556	–	–	
–	H_2O	~280, 290sh, 325, 375, 495, 550	–	–	112
ClO_4	MeOH:H2O	~325, 370, 490, 550	–	–	113
OTf	MeCN	~280, 290, 315, 490, 555	–	–	114
BF_4	MeCN 0.1 M Bu_4NBF_4	–	-1.64, -1.82	0.74	115
	MeCN	272, 280, 318, 551	–	–	
	DCM/MeCN (1:1 v:v) 0.01 M Bu_4NClO_4	–	–1.60, –1.77	0.75	116
–	DMF 0.1 M Bu_4NBF_4	~280, 320, 375, 600	$E_{1/2}$= –1.477, –1.661	$E_{1/2}$ = 0.895	117
–	MeCN	~500, 550	–	–	118
–	H_2O	–	–	1.062	119
I	acetate buffer	~360, 500, 550	–	–	120

Table S9. The absorption and the relevant electrochemical data properties of Fe(II) complexes.

medium	λ_{abs} [nm]	$E_{red,peak}$ [V] (ΔE [mV])/ $E_{red,onset}$ [V]/ $E_{1/2red}$[V]	$E_{ox,peak}$ [V]/$E_{ox,onset}$ [V] /$E_{1/2ox}$ [V]	Reference
**Fe1**				
DMSO	~340, 425, 590	–	–	36
MeCN	~330, 420, 580	–	–	
EtOH	~330, 420, 580	–	–	
H_2O	~330, 420, 580	–	–	
**Fe2**				
DCM	440, 576	–	–	48
**Fe3**				
DCM	321, ~430, 580	–	–	121
MeCN 0.1 M Bu_4NPF_6	–	–	0.69	
**Fe4**				
DMF	~300, 415, 580	–	–	86
DMF 0.1 M Bu_4NPF_6	–	–1.34 (60), –1.43 (60), $–2.15_{irr}$	–	
**Fe5**				
MeOH	233sh, 277, 285, 320, 378, 577	–	–	24
MeCN 0.1 M Bu_4NPF_6	–	–1.70, –1.78	0.62_{irr}	
**Fe6**				
MeCN	283, 320, 375, 576	–	–	122
MeCN 0.1 M Bu_4NPF_6	–	+1.12	1.03, 1.21	

Table S10. The absorption and the relevant electrochemical data properties of model Ru(II) complexes.

X^-	medium	λ_{abs} [nm]	λ_{exc} [nm]	λ_{PL} [nm]	τ [ns]	φ_{PL}	$E_{red,peak}$ [V] (ΔE [mV])/ $E_{red,onset}$ [V]/ $E_{1/2red}$[V]	$E_{ox,peak}$ [V]/$E_{ox,onset}$ [V] /$E_{1/2ox}$ [V]	Reference
**$[Ru(C_6H_4\text{-terpy})_2]X_2$**									
PF_6	DMF	282, 312, 328sh, 487	–	–	–	–	–	–	85
	DMF 0.1 M Bu_4NPF_6	–	–	–	–	–	–	1.27	
	DMF	318, 494	–	–	–	–	–	–	123
	DMAc	–	500	629	–	–	–	–	
	MeCN 0.1 M Bu_4NPF_6	–	–	–	–	–	$E_{1/2}$= –1.638	$E_{1/2}$=0.802	
	MeCN	487	–	715	0.001	0.00004	–	–	124
	BuCN 77 K	–	–	629	11.9	–	–	–	
	MeCN 0.1 M Bu_4NBF_4	–	–	–	–	–	–1.66	0.90	
	DMSO 0.1 M Bu_4NClO_4	–	–	–	–	–	$–1.15(75)_{qr}$, $–1.37(80)_{qr}$	1.26_{qr}	125
	EtOH:MeOH (4:1 v:v) 77 K	–	–	~625, 715	–	–	–	–	126
	EtOH:MeOH (4:1 v:v) 2 K	–	–	~667, 741	–	–	–	–	
	MeOH	284, 309, 488, 562	–	–	–	–	–	–	127
	EtOH:MeOH (4:1 v:v) 77 K	–	–	629, 680	12.3	0.45	–	–	
	EtOH:MeOH (4:1 v:v) 42 K	–	–	–	179	–	–	–	

	EtOH:MeOH (4:1 v:v) 18 K	–	–	–	321	–	–	–	
TFSI	DMF	288, 315, 494	–	–	–	–	–	–	128
	DMF 77 K	–	350	636	<0.01	–	–	–	
	DMF 0.1 M LiTFSI	–	–	–	–	–	–1.64, –1.8$_r$	0.79	
Cl	MeOH	~290, 320, 495	–	–	–	–	–	–	129
**X^-**	**medium**	**λ_{abs} [nm]**	**λ_{exc} [nm]**	**λ_{PL} [nm]**	**τ [ns]**	**φ_{PL}**	**$E_{red,peak}$ [V] (ΔE [mV])/ $E_{red,onset}$ [V]/ $E_{1/2red}$[V]**	**$E_{ox,peak}$ [V]/$E_{ox,onset}$ [V] /$E_{1/2ox}$ [V]**	**Reference**
**$[Ru(terpy)_2]X_2$**									
PF_6	MeCN	204, 231, 271, 308, 330sh, 475	–	–	–	–	–	–	130
	MeCN 0.1 M Me_4NBF_4	–	–	–	–	–	$E_{1/2}$= –1.418^r, –1.672^r	$E_{1/2}$=1.025^r	
	MeCN	306, 490	–	–	0.25	–	–	–	131
	MeCN 0.01 M Bu_4NClO_4	–	–	–	–	–	$E_{1/2}$= –1.24	$E_{1/2}$=1.30	
	EtOH:MeOH (4:1 v:v) 70 K	–	–	~600, 650	–	–	–	–	126
	EtOH:MeOH (4:1 v:v) 4 K	–	–	~615, 650	–	–	–	–	
	EtOH:MeOH (4:1 v:v) 2 K	–	–	~590, 615, 650	–	–	–	–	
	EtOH	270, 306, 473	–	–	–	–	–	–	
	MeCN	–	532	650	0.6	<0.0001	–	–	132
	MeCN	–	–	650	0.56	<0.0001	–1.27	1.28	133
	MeCN 0.1 M Bu_4NPF_6	–	–	–	–	–	–	$E_{1/2}$=1.28^r	134
	Acetone 0.1 M Bu_4NPF_6	–	–	–	–	–	–	$E_{1/2}$=1.32^r	
	MeCN	475	–	–	–	–	–	–	
	Acetone	476	–	–	–	–	–	–	
	H_2O	474	–	–	–	–	–	–	
	MeCN 0.1 M Bu_4NPF_6	–	–	–	–	–	–1.57^r (60), –1.83^r (70)	0.97^r	135
	MeCN	474	–	–	–	–	–	–	
PF_6	MeCN	~310, 355	–	–	–	–	–	–	136
	–	–	–	–	–	–	–1.27(80), –1.52	1.28	
	MeCN 0.1 M Bu_4NBF_4	–	–	–	–	–	–	0.91	137
	MeCN	475	–	630	0.3	<0.0001	$E_{1/2}$ = –1.23	–	138
	50% glicerol 77 K	~260, 310, 475	440	600	–	–	–	–	139
	MeCN	476	–	–	–	–	–	–	140
	MeCN 0.1 M Bu_4NBF_4	476	–	–	–	–	$E_{1/2}$ = –1.02, –1.27	$E_{1/2}$ = 1.52	
	MeCN 0.1 M Bu_4NClO_4	–	–	–	–	–	$E_{1/2}$ = –1.22, –1.46	$E_{1/2}$ = 1.32	141
	DCM	307, 475	–	–	–	–	–	–	
**$[Ru(terpy)_2]X_2$**									
–	MeCN 0.1 M Bu_4NPF_6	–	–	–	–	–	$E_{1/2}$ = –1.01^r (80), –1.26^r (103)	$E_{1/2}$ = –1.50^r	142
	H_2O	474	–	–	–	–	–	–	
	MeCN	475	–	–	–	–	–	–	
PF_6		270, 307, 475	–	–	–	–	–	–	143
		~310, 475	–	–	–	–	–	–	98
	–	–	–	–	–	–	–1.67	0.092	144
	MeCN	–	–	640	0.56	–	–1.25, –1.52	1.30	145
	MeCN 0.1 M Bu_4NClO_4	–	–	–	–	–	–	$E_{1/2}$=1.37	146
	DMF 0.1 M Bu_4NClO_4	–	–	–	–	–	$E_{1/2}$ = –1.30, –1.27	–	
	MeCN	475	476	625	–	–	–	–	
	BuCN 77 K	–	476	600	10900	–	–	–	
	MeCN	474	–	–	–	–	–	–	147
	MeCN 0.1 M Bu_4NPF_6	–	–	–	–	–	–	$E_{1/2}$=0.98	
	MeCN 0.1 M Bu_4NBF_4	–	–	–	–	–	–1.67, –1.92	0.92	115
	MeCN	270, 307, 475	–	–	–	–	–	–	
	MeOH	482	–	–	–	–	–	–	148
	MeCN 0.1 M Bu_4NBF_4	–	–	–	–	–	–1.58, –1.82	0.90	
	MeCN 0.2 M Bu_4NBF_4	–	–	–	–	–	$E_{1/2}$ = –1.25, –1.52^r	$E_{1/2}$ = 1.30	149
	MeCN	–	440	640	0.62	<0.0002	–	–	
	MeCN	269, 306, 478	–	–	–	–	–	–	150
	DCM Bu_4NOH	–	–	–	–	–	$E_{1/2}$ = –1.19, –1.52	$E_{1/2}$ = 1.19	
	EtOH:MeOH (4:1 v:v)	470	–	–	–	–	–	–	151
	MeCN 0.1 M Bu_4NBF_4	–	–	–	–	–	$E_{1/2}$ = –1.65, –1.90	$E_{1/2}$ = 0.89	152
	DMF 0.1 M Bu_4NBF_4	–	–	–	–	–	$E_{1/2}$ = –1.266, –1.510, –1.948, –2.35	$E_{1/2}$ = 1.264	153
	MeCN	–	–	650	0.56	<0.0001	–	–	154
	MeCN 0.1 M Bu_4NPF_6	–	–	–	–	–	$E_{1/2}$ = –1.66 (63), –1.90 (63)	$E_{1/2}$ = 0.89	155
	MeCN	225sh, 270, 308, 475	–	–	–	–	–	–	
	MeCN	–	–	615	0.125	$1\cdot10^{-6}$	–	–	156
	DMA (dimethylacetamide)	–	–	620	0.235	–	–	–	

	BuCN:EtOH (9:1 v:v) 77 K	–	–	604	11100	–	–	–	
	MeCN	232, 243, 272, 308, 335, 481	–	–	–	–	–	–	157
	MeCN 0.1 M Bu_4NPF_6	–	–	–	–	–	–1.21^r, –1.42^r	1.33^r	
	EtOH:MeOH (4:1 v:v)	309, 476	–	–	–	0.38	–	–	158
	EtOH:MeOH (4:1 v:v) 100 K	–	–	602	8850	–	–	–	
	EtOH:MeOH (4:1 v:v) 140 K	–	–	–	360	–	–	–	
	MeCN 0.1 M Bu_4NBF_4	–	–	–	–	–	$E_{1/2}$ = – 1.24,–1.49	$E_{1/2}$=1.30	
	DMSO 0.1 M Me_4NBF_4	–	–	–	–	–	$E_{1/2}$ = –1.48, –1.73	$E_{1/2}$=0.965	159
–	MeCN	476	–	–	–	–	–	–	160
	MeCN 0.1 M Bu_4NPF_6	–	–	–	–	–	–1.25	–	
PF_6	H_2O	269, 308, 476	–	–	–	–	–	–	161
	MeCN	–	–	629	–	<$5\cdot10^{-6}$	–	–	
	EtOH:MeOH (4:1 v:v) 77 K	–	–	598	10800	–	–	–	
	MeCN 0.1 M Bu_4NPF_6	–	–	–	–	–	$E_{1/2}$ = –1.21	$E_{1/2}$ = 1.38	
	MeOH	270, 280, 310, 330, 470	470	–	–	–	–	–	127
	EtOH:MeOH (4:1 v:v) 77 K	–		599, 649	11000	0.48	–	–	
	EtOH:MeOH (4:1 v:v) 42 K	–		–	282000	–	–	–	
	EtOH:MeOH (4:1 v:v) 18 K	–		–	342000	–	–	–	
	MeCN 0.1 M Bu_4NClO_4	–	–	–	–	–	$E_{1/2}$ = –1.19, –1.52	$E_{1/2}$ = 1.19	162
	MeCN	269, 306, 478	–	–	–	–	–	–	
	EtOH:MeOH (4:1) v:v) 77 K	–	–	599, 648	11000	–	–	–	
	MeCN	476	–	–	–	–	–	–	163
	MeCN 0.1 M Bu_4NPF_6	–	–	–	–	–	$E_{1/2}$ = –1.02, –1.27	$E_{1/2}$ = 1.52	
	MeOH	475	–	–	–	–	–	–	52
	MeCN	–	449	619	–	–	–	–	
	MeOH	308, 475	–	619	–	–	–	–	164
	MeCN 0.1 M Bu_4NBF_4	–	–	–	–	–	–	$E_{1/2}$=1.52	
	MeCN	476	–	–	–	–	–	–	140
	MeCN 0.1 M Bu_4NPF_6	–	–	–	–	–	$E_{1/2}$= –1.02, –1.27	$E_{1/2}$ =1.52	
**X^-**	**medium**	**λ_{abs} [nm]**	**λ_{exc} [nm]**	**λ_{PL} [nm]**	**τ [ns]**	**φ_{PL}**	**$E_{red,peak}$ [V] (ΔE [mV])/ $E_{red,onset}$ [V]/ $E_{1/2red}$[V]**	**$E_{ox,peak}$ [V]/$E_{ox,onset}$ [V] /$E_{1/2ox}$ [V]**	**Reference**
				**[Ru(terpy)$_2$]X$_2$**					
PF_6	DCM	476	–	~600	–	–	–	–	165
	Acetone	476	–	~615	–	–	–	–	
	H_2O	474	–	~615	–	–	–	–	
	MeCN	478	–	–	–	–	–	–	166
	PMMA	478	–	–	–	–	–	–	
	MeCN	474	–	629	–	<0.000005	–	–	124
	BuCN 77 K	–	–	598	10600	–	–	–	
	MeCN 0.1 M Bu_4NBF_4	–	–	–	–	–	-1.67^r	0.92^r	
	MeCN 0.1 M Bu_4NPF_6	–	–	–	–	–	$E_{1/2}$ = –1.27 (70), –1.51 (90)	$E_{1/2}$ = 1.27	167
	MeCN 0.1 M Bu_4NPF_6	–	–	–	–	–	$E_{1/2}$ = –1.21	–	168
	DCM 0.1 M Bu_4NPF_6	–	–	–	–	–	$E_{1/2}$ = –1.27 (110), –1.60 (147)	$E_{1/2}$ = 1.30	169
	MeCN 0.1 M Bu_4NPF_6	–	–	–	–	–	$E_{1/2}$= –1.27 (60), –1.51 (67)	$E_{1/2}$=1.27	170
	MeCN	310, 330, 475	–	–	–	–	–	–	
	EtOH:MeOH (4:1 v:v) 77 K	–	475	598	–	–	–	–	
	DCM	271, 309, 476	–	–	–	–	–	–	171
	MeCN 0.1 M Bu_4NPF_6	–	–	–	–	–	$E_{1/2}$ = –1.33 (90), –1.55 (78)	$E_{1/2}$ = 1.22	
	MeCN 0.1 M Bu_4NClO_4	–	–	–	–	–	$E_{1/2}$ = –1.22, –1.46	$E_{1/2}$ = 1.32	172
	MeCN	307, 475	–	–	–	–	–	–	
	MeCN 0.1 M Bu_4NClO_4	–	–	–	–	–	$E_{1/2}$ = –1.22, –1.46	$E_{1/2}$ = 1.32	173
	DCM	~270, 310, 475	–	–	–	–	–	–	
	MeCN 0.1 M Bu_4NClO_4	–	–	–	–	–	$E_{1/2}$= –1.22^r, –1.46	$E_{1/2}$=1.32	174
	BuCN	–	–	629	0.25	–	–	–	
	MeCN 0.1 M Bu_4NPF_6	–	–	–	–	–	–1.27 (60), –1.51 (70)	1.27	175
	MeCN	269, 307, 475	–	–	–	–	–	–	176
	solid	–	–	598	–	–	–	–	
	MeOH 77 K	–	–	–	–	0.48	–	–	
	MeCN 0.1 M Bu_4NPF_6	–	–	–	–	–	$E_{1/2}$ = –1.58, –1.82	$E_{1/2}$=0.92	
	EtOH:MeOH (3:1 v:v)	–	–		~0.01	–	–	–	177

	EtOH:MeOH (3:1 v:v) 77 K	–	–	621	6700	–	–	–	
ClO_4	EtOH:MeOH (3:1 v:v)	–	–	–	<0.01	–	–	–	
	EtOH:MeOH (3:1 v:v) 77 K	–	–	606	5000	–	–	–	
$Cl·6H_2O$	EtOH:MeOH (3:1 v:v)	~475	–	–	0.01	–	–	–	
	EtOH:MeOH (3:1 v:v) 77 K	~475	–	610	5800	–	–	–	
ClO_4	MeCN 0.1 M Bu_4NPF_6	–	–	–	–	–	–1.30 (65), –1.54(69), –2.00 (76), –2.34	1.27	153
I	EtOH:MeOH (4:1 v:v) 77 K	–	–	592<br>644	10820<br>10590	–	–	–	178
Cl	MeCN 0.1 M Bu_4NBF_4	–	–	–	<0.1	–	$E_{1/2}$ = –1.24, –1.49	$E_{1/2}$ = 1.30	179
	EtOH:MeOH (4:1 v:v)	271, 309, 476	–	–	–	–	–	–	
ClO_4	MeCN	470	–	–	–	–	–	–	180
**X⁻**	**medium**	**$λ_{abs}$ [nm]**	**$λ_{exc}$ [nm]**	**$λ_{PL}$ [nm]**	**τ [µs]**	**$φ_{PL}$**	**$E_{red,peak}$ [V] (ΔE [mV])/ $E_{red,onset}$ [V]/ $E_{1/2red}$[V]**	**$E_{ox,peak}$ [V]/$E_{ox,onset}$ [V] /$E_{1/2ox}$ [V]**	**Reference**
**[Ru(C_6H_4-terpy)(pyr-terpy)]X_2**									
PF_6	MeCN 0.1 M Bu_4NBF_4	–	–	–	–	–	–1.58, –1.89	0.89	181
	MeCN	238, 275, 311, 488	–	–	–	–	–	–	
**[Ru(terpy)(pyr-terpy)]X_2**									
PF_6	MeCN 0.1 M Bu_4NPF_6	–	–	–	–	–	–1.10, –1.40, –1.78^{irr}	1.37^{r}	182
	MeCN	229sh, 238, 273, 309, 324sh, 485	–	–	–	–	–	–	
	MeCN 0.1 M Bu_4NPF_6	–	–	–	–	–	–1.56, –1.86, –2.25, –2.61	0.97	183
	MeCN 0.1 M Bu_4NPF_6	–	–	–	–	–	–	1.32	184
	DMSO	278, 315, 490	469	654	–	–	–	–	185
	H_2O	–	467	654	0.018	0.0008	–	–	
**[Ru(terpy)(Me-C_6H_4-terpy)]X_2**									
PF_6	MeCN	~290, 320, 485	–	–	–	–	–	–	186
	MeCN 165 K	–	360	~650	–	–	–	–	
	DCM 0.1 M Bu_4NPF_6	–	–	–	–	–	$E_{1/2}$= –1.63	$E_{1/2}$=0.91	187
	DCM	274, 309, 327sh, 484	483	627	–	–	–	–	
**[Ru(C_6H_4-terpy)(MeOPh)$_2$Nterpy)]X_2**									
PF_6	–	–	–	–	–	–	$E_{1/2}$ = –1.28, –1.52	$E_{1/2}$ = 0.98,1.35, 1.44	173
	DCM	~280, 310, 360, 490	–	–	–	–	–	–	
	MeCN 0.1 M Bu_4NClO_4	–	–	–	–	–	$E_{1/2}$ = –1.30, –1.53	$E_{1/2}$ = 0.98, 1.35^{irr}, 1.44	188
	MeCN	274, 304, 496	–	–	–	–	–	–	
	MeCN 0.1 M Bu_4NClO_4	–	–	–	–	–	$E_{1/2}$ = –1.28, –1.52	$E_{1/2}$ = 0.98, 1.35^{irr}, 1.44	65
	MeCN	274, 303, 495	–	–	–	–	–	–	
	MeCN 0.1 M Bu_4NClO_4	–	–	–	–	–	$E_{1/2}$= –1.27^{r}, –1.50^{r}	$E_{1/2}$=0.98^{r}, 1.35^{irr}, 1.44^{r}	141
	DCM	497	–	–	–	–	–	–	
	MeCN	–	450	~670	–	–	–	–	
**[(H_2pbbzim)Ru(tpy-ph)]X_2**									
ClO_4	DCM	275sh, 286, 316, 337, 354, 402, 495	–	671	1.8, 5.5	0.00195	–	–	189
	EtOH:MeOH (4:1 v:v) 77 K	–	–	663	–	0.0019	–	–	
	MeCN 0.1 M Et_4NClO_4	–	–	–	–	–	$E_{1/2}$= –1.45, –1.84, –2.08	$E_{1/2}$ = 1.08	
**[(H_2pbbzim)Ru(tpy)]X**									
PF_6	MeCN	242, 270, 280, 313, 331, 348, 407, 477	–	–	–	–	–	–	190
	MeCN BR buffer	–	–	–	–	–	–	0.71	
	MeCN 0.1 M Et_4NPF_6	–	–	–	–	–	$E_{1/2}$ = –1.49,–1.90, –2.07	$E_{1/2}$ = 0.94	191
ClO_4	MeCN:H_2O (1:4 v:v)	~410, 490	–	–	–	–	–	0.99	192
	MeCN	235, 269, 276sh, 318, 329, 347, 402sh, 475	460	812	13.9	0.00078	–	–	193
	MeCN 77 K	–		713	1.2	–	–	–	
	MeCN Et_4NClO_4	–	–	–	–	–	–	$E_{1/2}$=1.11	
	DCM	272, 282sh, 315, 337, 354, 406sh, 484	484	660	–	0.00074	–	–	189
			450	–	1.2, 7.0	–	–	–	
	Solid	–	–	~680	–	–	–	–	
	DMSO	–	450	–	8.0, 31.0	–	–	–	
	EtOH:MeOH (4:1 v:v) 77 K	–	484	654	–	0.17	–	–	
	MeCN 0.1 M Et_4NClO_4	–	–	–	–	–	$E_{1/2}$ = –1.48, –1.90, –2.07	$E_{1/2}$ = 1.09	
	MeCN	~240, 280, 320, 360, 478	–	660	–	–	–	–	194
**[Ru(tpy)(tpy-COOH)]X_2**									

PF_6	MeCN	~350, ~380, ~425, 487	–	–	–	–	–	–	155
	MeCN	479	–	–	–	–	–	–	163
	MeCN 0.1 M Bu_4BF_4	–	–	–	–	–	$E_{1/2}$ = –1.05, –1.33	$E_{1/2}$ = 1.55	
	MeCN	272, 309, 484	450	666	–	–	–	–	195
	PBS	272, 309, 480	–	–	–	–	–	–	
**X^-**	**medium**	**λ_{abs} [nm]**	**λ_{exc} [nm]**	**λ_{PL} [nm]**	**τ [µs]**	**φ_{PL}**	**$E_{red,peak}$ [V] (ΔE [mV])/ $E_{red,onset}$ [V]/ $E_{1/2red}$[V]**	**$E_{ox,peak}$ [V]/$E_{ox,onset}$ [V] /$E_{1/2ox}$ [V]**	**Reference**
***cis*-(Cl,Cl)-[Ru(Phtpy)Cl_2(NO)]X**									
PF_6	MeCN	284, 330	–	–	–	–	–	–	196
***trans*-(Cl,Cl)-[Ru(Phtpy)Cl_2(NO)]X**									
PF_6	MeCN	270, 289, 298, 350	–	–	–	–	–	–	196
**[Ru(tpy)Cl_2(NO)]X**									
PF_6	MeCN 0.1 M Et_4NClO_4	–	–	–	–	–	$E_{1/2}$ = –0.51, –1.12^{irr}, –1.73^{irr}	–	197
	MeCN	296sh, 352, 381sh, 477	–	–	–	–	–	–	198
	MeCN 0.1 M Et_4NClO_4	–	–	–	–	–	$E_{1/2}$ = –0.5, –1.01^{irr}, –0.78^{irr}	–	
Cl	MeCN	274, 330, 349	–	–	–	–	–	–	199

Table S11. The absorption and the relevant electrochemical data properties of Ru(II) complexes.

medium	λ_{abs} [nm]	λ_{exc} [nm]	λ_{PL} [nm]	τ [µs]	φ_{PL}	$E_{red,peak}$ [V] (ΔE [mV])/ $E_{red,onset}$ [V]/ $E_{1/2red}$[V]	$E_{ox,peak}$ [V]/$E_{ox,onset}$ [V] /$E_{1/2ox}$ [V]	Reference
**Ru1**								
DCM	417, 490	–	–	–	–	–	–	200
DCM 0.1 M Bu_4NPF_4	–	–	–	–	–	–	$E_{1/2}$ = 0.77, 1.00	
**Ru2**								
MeCN	300, 320, 350, 516	–	–	–	–	–	–	31
MeCN 0.1 M Bu_4NPF_6	–	–	–	–	–	– 0.15, –0.47^{irr}, –1.27^{irr}	–	
**Ru3**								
MeCN	301, 322, 347, 550	–	–	–	–	–	–	31
MeCN 0.1 M Bu_4NPF_6						–0.24, –0.57^{irr}, –1.31^{irr}	–	
**Ru4**								
MeCN	370, 420	–	–	–	–	–	–	201
MeCN 0.1 M Bu_4NPF_6	–	–	–	–	–	$E_{1/2}$ = –0.082, –0.029, –0.550	$E_{1/2}$ = 0.11	
DCM 0.1 M Bu_4NPF_6	–	–	–	–	–	$E_{1/2}$ = –0.008, 0.120, 0.637	$E_{1/2}$ = 0.128	
Acetone0.1 M Bu_4NPF_6	–	–	–	–	–	$E_{1/2}$ = –0.052, 0.060, 0.613	$E_{1/2}$ = 0.112	
DMF 0.1 M Bu_4NPF_6	–	–	–	–	–	$E_{1/2}$= –0.084, 0.031, 0.559	$E_{1/2}$ = 0.115	
**Ru5**								
MeCN	277, 301, 515	–	–	–	–	–	–	57, 65
MeCN 0.1 M Bu_4NClO_4	–	–	–	–	–	$E_{1/2}$ = –1.30, –1.54	$E_{1/2}$ = 0.82, 1.02, 1.36^{irr}, 1.48	57
	–	–	–	–	–	$E_{1/2}$ = –1.30, –1.4^{r}	$E_{1/2}$= 0.82, 1.02, 1.31^{irr} 1.48	65
**Ru6**								
MeCN	272, 308, 496	–	–	–	–	–	–	65
MeCN 0.1 M Bu_4NClO_4	–	–	–	–	–	$E_{1/2}$ = –1.24, –1.49	$E_{1/2}$ = 0.82, 1.30^{irr} 1.36	
**Ru7**								
MeCN	275, 310, 516	–	–	–	–	–	–	65
MeCN 0.1 M Bu_4NClO_4	–	–	–	–	–	$E_{1/2}$ = – 1.30, –1.54	$E_{1/2}$ = 0.82, 1.02, 1.31^{irr}, 1.36^{irr}, 1.48,	
MeCN	275, 310, 404, 516	–	–	–	–	–	–	57
MeCN 0.1 M Bu_4NClO_4	–	–	–	–	–	$E_{1/2}$ = –1.26, –1.46	$E_{1/2}$ = 0.81, 1.30^{irr}, 1.37	
MeCN	284, 310, 502	–	–	–	–	–	–	202
MeCN 90 K	–	–	656	13.9	–	–	–	
MeCN 150 K	–	–	678	0.4	–	–	–	
MeCN 200 K	–	–	676	0.04	–	–	–	
MeCN	277, 310, 516	–	–	–	–	–	–	203
MeCN 0.1 M $Bu_4NCF_3SO_3$	–	–	–	–	–	–1.04, –1.23	1.57	
**Ru8**								
MeCN	547	–	–	–	–	–	–	204
**Ru9**								
MeCN	525	–	–	–	–	–	–	204
**Ru10**								
MeCN	529	–	–	–	–	–	–	204
**Ru11**								

MeCN	283, 308, 500	469	612	–	0.031	–	–	205
DCM 0.1 M Bu_4NBF_4						– 0.91, –1.26	1.26	
**Ru12**								
MeCN	287, 310, 497	–	–	–	–	–	–	206
MeCN	~240, 290, 310, 495	–	–	–	–	–	–	207
**Ru13**								
MeOH	414, 493	–	–	–	–	–	–	164
MeCN	–	493	660	1.1	0.00018	–	–	
DCM 0.1 M Bu_4NBF_4						– 0.91, –1.26	1.26	
**Ru14**								
MeCN	274, 311, 500	–	–	–	–	–	–	203
EtOH	–	–	686	–	–	–	–	
Propylene carbonate 0.1 M $Bu_4NCF_3SO_3$	–	–	–	–	–	–1.09	1.50	
EtOH	274, 311, 504	–	–	–	–	–	–	208
**Ru15**								
MeCN 0.1 M Bu_4NPF_4						– 1.60	0.49, 0.86	51
DCM	~ 290, ~315, 405, 421, 532	375	~467	–	–	–	–	
		400		–	–	–	–	
		432	~633	–	–	–	–	
	519	–	–	–	–	–	–	48
MeCN	310, 399, 507	–	–	–	–	–	–	209
MeCN 0.1 M Bu_4NClO_4							$E_{1/2}$ = 0.98, 1.29	
**Ru17**								
MeCN	283, 310, 504	–	–	–	–	–	–	202
MeCN 90 K	–	–	656	13.4	–	–	–	
MeCN 150 K	–	–	675	0.1	–	–	–	
MeCN 0.1 M Bu_4NClO_4	–	–	–	–	–	$E_{1/2}$ = –0.40, –1.26	$E_{1/2}$ = 0.75, 1.26	
**Ru18**								
DMF	~280, 325, 400, 500	–	–	–	–	–	–	52
DMF 0.1 M Bu_4NClO_4	–	–	–	–	–	–1.12, –1.39, –1.85	1.17, 1.45	210
**Ru19**								
MeOH	235, 276, 285, 308, 507	–	–	–	–	–	–	24
MeCN 0.1 M Bu_4NPF_6	–	–	–	–	–	–1.70, –1.94	0.74^{irr}	
MeCN	~300, 360, 500	–	–	–	–	–	–	211
**Ru20**								
MeCN	288, 310, 501	–	–	–	–	–	–	122
**Ru24**								
DMF	291, 318, 410, 508	–	–	–	–			210
DMF 0.1 M Bu_4NClO_4	–	–	–	–	–	–1.08, –1.32, –1.60	1.16, 1.38	
**Ru25**								
DMF	~290, 325, 410, 510	–	–	–	–	–	–	210
DMF 0.1 M Bu_4NClO_4	–	–	–	–	–	–1,10, –1.35, –1.73	1.18, 1.43	

Table S12. The absorption and the relevant electrochemical data properties of model Os(II) complexes.

**[Os(Ph-terpy)$_2$]X$_2$**									
**X^-**	**medium**	**λ_{abs} [nm]**	**λ_{exc} [nm]**	**λ_{PL} [nm]**	**τ [μs]**	**φ_{PL}**	**$E_{red,peak}$ [V] (ΔE [mV])/ $E_{red,onset}$ [V]/ $E_{1/2red}$[V]**	**$E_{ox,peak}$ [V]/$E_{ox,onset}$ [V] /$E_{1/2ox}$ [V]**	**Reference**
PF_6	DMSO	~505, 595, 680	~500, 675	~760	–	–	–	–	212
	MeCN 0.1 M Bu_4NPF_6	–	–	–	–	–	$E_{1/2}$ = –1.15, –1.42	$E_{1/2}$ = 0.94	213
		–	–	–	–	–	$E_{1/2}$ = – 1.58, –1.863	$E_{1/2}$ = 0.52, 1.67	123
	MeCN	–	500	740	0.23 2	0.021	–	–	
	DMF	319, 494, 671	–	–	–	–	–	–	
	DCM	–	670	~720	–	–	–	–	214
TFSI	DMF	289, 318, 493, 646, 672	494	757	0.20	0.054	–	–	128
	DMF 77 K	–	493	730	–	–	–	–	
	DMF 0.1 M $LiC_2F_6NO_4S_2$	–	–	–	–	–	–1.60, –1.88	0.45	
**[Os(terpy)$_2$]X$_2$**									
PF_6	MeCN 0.1 M Bu_4NClO_4	–	–	–	–	–	$E_{1/2}$= –1.20, –1.48	$E_{1/2}$ = 1.01	57

	MeCN	270, 310, 476, 662	480, 650	~710	–	–	–	–	
	DMSO	~480, 550, 640, 675	~490, 675	~745	–	–	–	–	212
	MeCN	310, 475, 656	–	–	–	–	–	–	176
	Solid	–	–	689	–	–	–	–	
	MeCN 0.1 M Bu_4NPF_6	–	–	–	–	–	$E_{1/2}$ = –1.63, –1.95	$E_{1/2}$ = 0.58	
	MeCN 0.1 M Bu_4NBF_4	–	–	–	–	–	–1.89, –1.60	0.54, 1.60^{irr}	152
	MeCN:H_2O (1:1 v:v)	476, 656	480	715	–	–	–	–	215
	MeCN	689	–	729	0.269	–	–	–	216
	MeCN 0.1 Me_4NClO_4	–	–	–	–	–	$E_{1/2}$ = –1.25	$E_{1/2}$ = 0.97	
	BuCN	~230, 280, 320, 475, 540, 660	–		–	–	–	–	138
	MeCN			710	0.27	0.014			
		–	630	720	0.270	0.014	–	–	149
	MeCN 0.2 M Bu_4NBF_4	–	–	–	–	–	$E_{1/2}$ = –1.27, –1.55	$E_{1/2}$ = 0.96	
		–	–	–	–	–	–1.63, –1.95	0.58	115
	MeCN	229, 270, 310, 475, 656	–	–	–	–	–	–	
TFSI	DMF	~280, 315, 475, 550, 660	480	732	–	–	–	–	217
I	EtOH:MeOH (4:1 v:v) 77 K	–	–	–	3.9	0.112	–	–	218
ClO_4	MeCN	310, 474, 656	–	713	–	–	–	–	219
	H_2SO_4	–	–	–	0.14	–	–	–	
	MeCN 0.1 M Et_4NClO_4	–	–	–	–	–	– 0.86, –1.28	0.49, 0.91	
Cl	MeCN 0.1 M Bu_4NBF_4						–1.23, –1.52	0.97	179
	EtOH:MeOH (4:1 v:v) 77 K	227, 271, 312, 477, 657	–	718	–	0.014	–	–	
	MeCN	–	–	–	0.269	–	–	–	

Table S13. The absorption, emission and the relevant electrochemical data properties of Os(II) complexes.

medium	λ_{abs} [nm]	λ_{exc} [nm]	λ_{PL} [nm]	τ [μs]	φ_{PL}	$E_{red,peak}$ [V] (ΔE [mV])/ $E_{red,onset}$ [V]/ $E_{1/2red}$[V]	$E_{ox,peak}$ [V]/$E_{ox,onset}$ [V] /$E_{1/2ox}$ [V]	Reference
**Os1**								
MeCN	284, 314, 396, 502, 672	650	720	–	~0.003	–	–	57
		480	720	–	–	–	–	
MeCN 0.1 M Bu_4NClO_4						$E_{1/2}$ = –1.20, –146	$E_{1/2}$ = 0.82, 1.00, 1.40^{irr}	
**Os2**								
MeCN	278, 314, 352, 515, 676	680	720	–	–	–	–	57
		480	720	–	–	–	–	
MeCN 0.1 M Bu_4NClO_4	–	–	–	–	–	$E_{1/2}$= –1.22, –1.50	$E_{1/2}$ = 0.79, 1.02, 1.31^{irr}	
**Os3**								
MeCN	313, 503, 644, 674	–	–	–	–	–	–	206
MeCN	~310, 400, 500, 640, 680	532	–	–	–	–	–	207
MeCN	–	308	764	0.057	0.0024	–	–	220
MeCN 0.1 M Bu_4NPF_6	–	–	–	–	–	$E_{1/2}$ = –0.93, –1.01, –1.24	$E_{1/2}$ = 0.77, 1.03	
**Os4**								
MeCN	–	308	734	0.247	0.02	–	–	220
MeCN	238, 276, 313, 349, 380sh, 512, 653, 676	–	–	–	–	–	–	206
**Os5**								
MeCN	278, 310, 354, 516, 584	–	–	–	–	–	–	57
MeCN 0.1 M Bu_4NClO_4						$E_{1/2}$ = –1.28, –1.53	$E_{1/2}$ = 0.70, 0.85, 1.30^{irr}	
**Os6**								
MeCN	231, 288, 313, 386sh, 501, 648, 672	–	–	–	–	–	–	206

Table S14. The absorption, emission and the relevant electrochemical data properties of model Cr(III) complexes.

X^-	medium	λ_{abs} [nm]	λ_{exc} [nm]	λ_{PL} [nm]	τ [µs]	φ_{PL}	$E_{red,peak}$ [V] (ΔE [mV])/ $E_{red,onset}$ [V]/ $E_{1/2red}$[V]	$E_{ox,peak}$ [V]/$E_{ox,onset}$ [V] /$E_{1/2ox}$ [V]	Reference
**[Cr(C_6H_4-terpy)$_2$]X_3**									
PF_6	MeCN	365, 353, 443	353	785	0.3	–	–	–	221
	MeCN 0.1 M Bu_4NPF_6	–	–	–	–	–	$E_{1/2}$ = – 0.54, –0.89, –1.42	$E_{1/2}$ = 1.04	
**[Cr(terpy)$_2$]X_3**									
PF_6	1 M HCl	~ 350, 370	–	–	–	–	–	–	221
	MeCN	314, 326, 350, 364, 421	–	770	0.14	–	–	–	
	MeCN 0.1 M Bu_4NPF_6	–	–	–	–	–	$E_{1/2}$ = –0.53, –0.94, –1.44	1.08	
	MeCN 0.1 M Bu_4NPF_6	–	–	–	–	–	– 1.47	–	222
	H_2O	~ 265, 290, 340, 370	–	–	–	–	–	–	
	MeCN	265, 282sh, 290, 315, 325, 350, 365, 421, 442, 474, 525, 530	355	711, 771	<2	–	–	–	223
	MeCN 77 K	–		771, 782, 788, 796		–	–	–	
	Solid	715, 730, 773		771, 784, 798	<2	–	–	–	
	Solid 77 K	–		772, 784, 800	8.2	–	–	–	
	MeCN:C_2H_5CN (6:4 v:v) 77 K	–		–	670	–	–	–	
	H_2O	–	–	–	–	<0.001	–	–	
–	MeOH:EtOH (1:4 v:v) 77 K	422, 443, 473	–	–	–	–	–	–	224
ClO_4	H_2O	225, 239, 267, 286, 315, 327, 348, 364, 422, 443, 473	340	770	–	–	–	–	225
	MeCN 0.1 M Bu_4NClO_4	–	–	–	–	–	$E_{1/2}$ = –0.14, –0.55, –1.05, –1.99, –2.37	–	
	MeCN 0.1 M Bu_4NBF_4	–	–	–	–	–	$E_{1/2}$ = –0.11 (60), –0.52 (60), –1.03 (60), –1.95 (70)	–	226
		–	–	–	–	–	$E_{1/2}$ = –0.12 (60), 0.52 (60), 1.03 (60), 1.95 (70)	–	227
	MeCN	222, 233sh, 264, 266sh, 277sh, 285sh, 315sh, 325, 335sh, 347, 349sh, 364, 418, 442, 472	–	–	–	–	–	–	
**[Cr(Me-C_6H_4-terpy)$_2$]X_3**									
CF_3SO_3	MeCN 0.1 M Bu_4NPF_6	–	–	–	–	–	$E_{1/2}$ = –0.533, –0.953, –1.469	–	228
	MeCN	~290, 380, 480	–	–	–	–	–	–	
PF_6	MeCN	367, 357, 441, 469	357	788	0.4	–	–	–	221
	MeCN 0.1 M Bu_4NPF_6	–	–	–	–	–	$E_{1/2}$ = –0.60, –0.95, –1.44	0.97	
ClO_4	MeCN 0.1 M Bu_4NBF_4	–	–	–	–	–	$E_{1/2}$ = –0.47 (70), –0.85 (70), –1.35 (70), –2.36 (80)	$E_{1/2}$ = 1.01 (60)	229
	MeCN	366, 440, 475	400	770	0.27	0.0005	–	–	
	H_2O	225, 239, 267, 286, 315, 327, 348, 364, 422, 443, 473, 576	340	773	–	–	–	–	225

Table S15. The absorption, emission and the relevant electrochemical data properties of Cr(III) complexes.

medium	λ_{abs} [nm]	$E_{red,peak}$ [V] (ΔE [mV])/ $E_{red,onset}$ [V]/ $E_{1/2red}$[V]	$E_{ox,peak}$ [V]/$E_{ox,onset}$ [V] /$E_{1/2ox}$ [V]	Reference
**Cr1**				
MeCN	360, 532, 691	–	–	221
MeCN 0.1 M Bu_4NPF_6	–	$E_{1/2}$= –0.69, –1.03, –1.46	–	
H_2O	~532	–	–	
DMF	~525	–	–	
MeOH	~532	–	–	
Acetone	~532	–	–	
1 M HCl	~300, 360, 400	–	–	
**Cr2**				
MeCN 0.1 M Bu_4NPF_6	–	$E_{1/2}$= –0.59, –0.96, –1.28	$E_{1/2}$ = 0.66	228
MeCN	~290, 355, 365, 507, 665	–	–	
**Cr3**				
$CHCl_3$	~290, 355, 574, 750	–	–	228
DCM	~290, 355, 563, 730	–	–	
EtOH	~290, 355, 550, 730	–	–	
Acetone	~290, 355, 526,708	–	–	
MeCN	~290, 355, 365, 528, 708	–	–	

Table S16. The absorption and the relevant electrochemical data properties of model Ir(III) complexes.

X^-	medium	λ_{abs} [nm]	λ_{exc} [nm]	λ_{PL} [nm]	τ [µs]	φ_{PL}	$E_{red,peak}$ [V] (ΔE [mV])/ $E_{red,onset}$ [V]/ $E_{1/2red}$[V]	$E_{ox,peak}$ [V]/$E_{ox,onset}$ [V] /$E_{1/2ox}$ [V]	Reference
**[Ir(Ph-terpy-κ^{3}N)$_2$]X$_3$**									
CS[a]	solid	–	~400	559	3.75	0.18	–	–	230
	MeCN	~270, 310, 340, 350, 375	–	~500	–	–	–	–	
PF_6	MeOH:EtOH (1:4 v:v) 77 K	–	370	512	40.08	–	–	–	231
	DMSO	264, 300, 324, 348, 375, 457, 521	375, 520	567, 578	4.46	0.042	–	–	
	MeCN	298, 318, 340, 369, 450, 521	375, 520	522, 580	6.50	0.103	–	–	
	Solid	–	500	600	10.30	0.050	–	–	
**[Ir(terpy-κ^{3}N)$_2$]X$_3$**									
PF_6	DCM	~275, 340, 360	–	~785	–	–	–	–	232
	DMF	~275,340, 360	–	–	–	–	–	–	
	DMSO	~275, 340, 360	–	–	–	–	–	–	
	Ethyl acetate	~275, 340, 360	–	–	–	–	–	–	
	Ethylene glycol	~275, 340, 360	–	–	–	–	–	–	
	EtOH	~275, 340, 360	525	–	–	–	–	–	
	H_2O	~275, 340, 360	450	–	–	–	–	–	
	MeCN	~275, 340, 360	450	–	–	–	–	–	
	MeOH	~275, 340, 360	–	–	–	–	–	–	
	MeCN	~250, 280, 330, 340, 360	350	~455, 495, 540	1.4	0.03	–	–	233
	Solid	–	350	~500, 550	–	–	–	–	
	MeCN		–	491	0.71	–	–	–	234
NO_3	H_2O	–	–	491	1.19	–	–	–	
PF_6	MeCN	~250, 275, 315, 330, 355	–	–	–	–	–	–	
		251, 277, 313, 325,352	–	458	1.2	–	–	–	235
		–	313	–	–	–	–	–	236
	DMF 0.05 M TBAP	–	–	–	–	–	–0.67	–	
	MeCN 0.1 M Bu_4NPF_6	–	–	–	–	–	–0.77	–	75
	DMF 0.1 M Bu_4NPF_6	–	–	–	–	–	–0.76	–	
	MeCN	210, 221, 251, 277, 313, 325, 336, 352, 374	350	458	1.2	0.025	–	–	
	BuCN 77 K	–	–	458	26	–	–	–	
ClO_4	MeCN	280, 315, 328, 338	–	~460, 505, 535	0.07	0.000046	–	–	237
	DMF:DCM (9:1 v:v)	–	–		0.228	0.0002	–	–	
CF_3CO_2	MeCN:BuCN (1:5 v:v) 77 K	–	–	~460, 485, 490, 520, 535, 560	–	–	–	–	

I	Solid	~225, 280, 300, 315, 420	–	–	–	–	–	–	238
**X^-**	**medium**	**λ_{abs} [nm]**	**λ_{exc} [nm]**	**λ_{PL} [nm]**	**τ [µs]**	**φ_{PL}**	**$E_{red,peak}$ [V] (ΔE [mV])/ $E_{red,onset}$ [V]/ $E_{1/2red}$[V]**	**$E_{ox,peak}$ [V]/$E_{ox,onset}$ [V] /$E_{1/2ox}$ [V]**	**Reference**
**[$IrCl_3(C_6H_4$-terpy-κ^{3}N)]**									
–	EtOH:MeOH (4:1 v:v) 77 K	–	520	565	12.43	-	–	–	231
	DMSO	277, 290, 318, 333, 416, 459, 526	440, 520	600	1.75	0.378	–	–	
	MeCN	273, 285, 312, 328, 419, 489, 524	440, 520	604	1.98	0.0586	–	–	
	Solid	–	525	745	0.21	0.0248	–	–	
**[$IrCl_3$(terpy-κ^{3}N)]**									
–	MeCN	266, 282, 313, 327, 408, 523	327	584	0.29	0.075	–	–	239
	DMF 0.05 M TBAP	–	–	–	–	–	–1.1	1.69	
**[Ir(Ph-terpy-κ^{3}N)(dppy)]X**									
PF_6	DCM	280, 308, 445, 527	436	678	2.05	0.033	–	–	240
	MeCN	~325, 445, 520		671	1.89	0.028	–	–	
	THF	~325, 430, 525		675	1.97	0.033	–	–	
	Toluene:DCM (95:5 v:v)	~320, 440, 530		686	1.04	0.014	–	–	
**[IrCl(Ph-terpy-κ^{3}N)(Ph-py)]X**									
PF_6	MeCN 0.1 M Bu_4PF_6	–	–	–	–	–	–1.06 (78), –1.39, $–1.95^{irr}$	1.71	241
	MeCN	238, 275, 283, 300, 330sh, 369, 398, 505sh	380	547	1.433	0.132	–	–	
	EtOH:MeOH (4:1 v:v) 77 K	–	405	540,580	29.50	–	–	–	242
	$CHCl_3$	284, 309, 377, 398, 477, 524	510	571	3.83	0.312	–	–	
	MeCN	280, 298, 330, 370, 398, 479, 506	505	560	5.98	0.628	–	–	
	DMSO	282, 305, 332, 371, 399, 473, 512	505	563	5.77	0.697	–	–	
	Solid	–	550	650	8.25	0.046	–	–	
	MeCN	238, 275, 283sh, 300, 330sh, 369, 398sh, 505sh	380	547	3.99	0.48	–	–	243
	MeCN 0.1 M $TBABF_4$	–	–	–	–	–	$E_{1/2}$= –0.82(76), –1.04 (144)	–	
Cl	MeOH	370	–	560	–	0.475	–	–	244
	PBS:DMSO (99:1 v:v)	~285, 290, 310, 340, 360, 395	405	~570	–	–	–	–	
**[IrCl(terpy-κ^{3}N)(Ph-py)]X**									
PF_6	THF	237, 256, 272, 282, 305, 330, 390, 503	378	551	1.56	0.34	–	–	245
	MeCN	234, 270, 280, 300, 328, 363, 394, 501		554	3.06	0.77	–	–	
	H_2O	~275, 280, 330, 450, 525	405	~550	1.96	0.113	–	–	246
	MeOH	–		~550	–	–	–	–	
	Acetone	–		~560	–	–	–	–	
	DCM	–		~560	–	–	–	–	
	TCM	–		~550	–	–	–	–	
	DCM	273, 307, 329sh, 368, 400, 480, 512	400	550	2.9	0.81	–	–	247
	EtOH:MeOH (1:1 v:v) 77 K	–	300	531, 562sh	–	0.83	–	–	
	MeCN 0.1 M Bu_4NClO_4	–	–	–	–	–	$E_{1/2}$ = –2.39	$E_{1/2}$ = 1.29	
	MeCN	234, 270, 279sh, 301, 328, 389sh, 499sh	380	541	3.88	0.45	–	–	243
	MeCN 0.1 M $TBAPF_6$	–	–	–	–	–	$E_{1/2}$ = –0.84 (72), –1.04 (141)	–	
	MeCN 0.1 M Bu_4NPF_6	–	–	–	–	–	$E_{1/2}$ = –1.09 (61), –1.35, $–1.98^{irr}$	1.72, 209	241
	MeCN	234, 270, 279sh, 301, 328, 389sh, 499sh	380	541	1.674	0.15	–	–	
	MeCN	360,470,500		542	2.62		–	–	248
	MeCN 0.1 M NEt_4NBF_4	–	–	–	–	–	$E_{1/2}$ = –1.04	1.73	
Cl	PBS:DMSO (99:1 v:v)	~280, 285sh, 305, 330, 380	405	~550	–	–	–	–	244
	MeOH	365	–	550	–	0.185	–	–	
**[$Ir(Ph-py)_2$(Ph-terpy-κ^{3}N)]X**									
PF_6	MeOH:EtOH (1:4 v:v) 77 K	–	410	550	4.24	–	–	–	242
	$CHCl_3$	267, 289, 314, 378, 416, 477	470	628	0.21	0.23	–	–	
	MeCN	268, 336, 385, 472	470	631	0.16	0.403	–	–	
	DMSO	269, 288, 320, 386, 474	470	635	0.13	0.29	–	–	
	Solid	–	510	602	1.68	0.392	–	–	

X^-	medium	λ_{abs} [nm]	λ_{exc} [nm]	λ_{PL} [nm]	τ [µs]	φ_{PL}	$E_{red,peak}$ [V] (ΔE [mV])/ $E_{red,onset}$ [V]/ $E_{1/2red}$[V]	$E_{ox,peak}$ [V]/$E_{ox,onset}$ [V] /$E_{1/2ox}$ [V]	Reference
**[Ir(Ph-py)$_2$(terpy-κ^{3}N)]X**									
PF$_6$	Benzene	~325, 350	–	~580	–	–	–	–	249
	THF	~310, 350	–	~580	–	–	–	–	
	Ethyl acetate	~310, 375	–	~578	–	–	–	–	
	EtOH	~310, 360	–	~580	–	–	–	–	
	MeCN	~320, 360	–	~580	–	–	–	–	
	DMF	~305, 360	–	~580	–	–	–	–	
	DCM 0.1 M Bu$_4$NPF$_6$	–	–	–	–	–	$E_{1/2}$ = –1.82[qr]	$E_{1/2}$=0.90[ir]	181
	DCM	~260, 300, 370, 400	260	590	0.068	0.017	–	–	

[a] camphorsulfonate counter-anion

Table S17. The absorption, emission and the relevant electrochemical data properties of Ir(III) complexes.

medium	λ_{abs} [nm]	λ_{exc} [nm]	λ_{PL} [nm]	τ [µs]	φ_{PL}	$E_{red,peak}$ [V] (ΔE [mV])/ $E_{red,onset}$ [V]/ $E_{1/2red}$[V]	$E_{ox,peak}$ [V]/$E_{ox,onset}$ [V] /$E_{1/2ox}$ [V]	Reference
**Ir1**								
MeCN	255, 283, 321, 504	532	754	–	–	–	–	250
H_2O	510	–	–	–	–	–	–	
DCM	502	–	–	–	–	–	–	
**Ir2**								
MeCN	253, 288, 321, 493	532	784	–	–	–	–	250
H_2O	494	–	–	–	–	–	–	
DCM	492	–	–	–	–	–	–	
**Ir3**								
DMSO	263, 289, 312, 469	465, 555	720, 750	0.10	0.07	–	–	231
MeCN	282, 310, 322, 470		765, 780	0.07	0.0016	–	–	
Solid	–	500	735	0.58	<0.0005	–	–	
EtOH:MeOH (4:1 v:v) 77 K	–	465	632	90.45	–	–	–	
**Ir4**								
MeCN	215, 252, 281, 306, 459	380	506	7.3	0.7	–	–	251
H_2O	~215, 278, 305, 440		506	19	0.1	–	–	
MeOH	~215, 305, 340, 480	–	–	–	–	–	–	
MeCN 0.01 M Bu$_4$NPF$_6$	–	–	–	–	–	–1.16, –1.43	–	
**Ir5**								
MeCN	252, 280, 299, 340, 367, 448	380	495	4.7	2.6	–	–	251
H_2O	~252, 280, 425		501	14	0.3	–	–	
MeCN 0.01 M Bu$_4$NPF$_6$	–	–	–	–	–	–1.10, –1.29	–	
MeOH	~252, 280, 325, 470	–	–	–	–	–	–	
**Ir6**								
MeCN	252, 288, 295, 313, 359, 448	380	497	3.2	0.9	–	–	251
H_2O	–	–	497	15	1.0	–	–	
MeCN 0.01 M Bu$_4$NPF$_6$	–	–	–	–	–	–1.05, –1.38	–	
**Ir7**								
DCM	~305, 545	525	–	–	–	–	–	232
DMF	~305, 500	–	–	–	–	–	–	
DMSO	~305, 500	–	–	–	–	–	–	
Ethyl acetate	~305, 500	–	–	–	–	–	–	
Ethylene glycol	~305, 500	–	–	–	–	–	–	
EtOH	~305, 500	–	–	–	–	–	–	
H_2O	~305, 500	–	–	–	–	–	–	
MeCN	~305, 500	–	–	–	–	–	–	
MeOH	~305, 500	–	–	–	–	–	–	
**Ir8**								

DMSO	273, 289, 312, 325, 382, 422, 441, 493, 529	440, 520	650, 670	1.22	3.74	–	–	231
MeCN	289, 313, 324, 378, 428, 489, 521	–	611, 614	1.45	1.63	–	–	
Solid	–	530	710	0.21	4.38	–	–	
EtOH:MeOH (4:1 v:v) 77 K	–	520	639	10.90	–	–	–	
**medium**	**λ_{abs} [nm]**	**λ_{exc} [nm]**	**λ_{PL} [nm]**	**τ [μs]**	**φ_{PL}**	**$E_{red,peak}$ [V] (ΔE [mV])/ $E_{red,onset}$ [V]/ $E_{1/2red}$[V]**	**$E_{ox,peak}$ [V]/$E_{ox,onset}$ [V] /$E_{1/2ox}$ [V]**	**Reference**
				**Ir9**				
$CHCl_3$	465, 533	–	624	–	–	–	–	41
				**Ir10**				
H_2O	~290, 340, 460, 550	450	-	–	0.01	–	–	232
MeCN	~290, 330, 450, 525		~660	–	0.003	–	–	
EtOH	~290, 330, 450, 525	525	~660	–	0.009	–	–	
DCM	~290, 330, 450, 550	–	~670	–	–	–	–	
DMF	~290, 340, 450, 525	–	~660	–	–	–	–	
DMSO	~290, 330, 450, 525	–	~660	–	–	–	–	
Ethyl acetate	~290, 330, 450, 525	–	~660	–	–	–	–	
Ethylene glycol	~290, 330, 450, 525	–	~660	–	–	–	–	
MeOH	~290, 330, 450, 525	–	~660	–	–	–	–	
				**Ir11**				
DCM	280, 311, 461, 528	528	679	3.12	0.049	–	–	240
MeCN	~311, 450, 525	525	676	2.45	0.035	–	–	
THF	~311, 450, 520	520	677	3.04	0.05	–	–	
Toluene:DCM (95:5 v:v)	~325, 455, 520	520	678	1.89	0.036	–	–	
				**Ir12**				
DCM 0.1 M Bu_4NPF_6	–	–	–	–	–	$E_{1/2}$ = –1.74, –2.05	$E_{1/2}$ = 0.74	181
DCM	~270, 305, 345, 395	255	580	0.125	0.099	–	–	
				**Ir13**				
EtOH:MeOH (4:1 v:v) 77 K	–	405	545, 579	19.64	–	–	–	242
$CHCl_3$	265, 286, 308, 342, 393, 478	470	614	0.94	0.014	–	–	
MeCN	260, 306, 337, 392, 481	470	620	0.95	0.038	–	–	
DMSO	266, 295, 342, 394, 480	470	635	1.53	0.01	–	–	
Solid	–	490	636	2.09	0.011	–	–	
				**Ir14**				
H_2O	~285, 315, 425	–	591	0.003	0.64	–	–	35
DCM	–	–	572	–	–	–	–	
Ethyl acetate	–	–	572	–	–	–	–	
EtOH	–	–	599	–	–	–	–	
MeOH	–	–	600	–	–	–	–	
DMSO	–	–	605	–	–	–	–	
				**Ir15**				
EtOH:MeOH (4:1 v:v) 77 K	–	405	616	16.65	–	–	–	242
$CHCl_3$	278, 330, 443, 476, 513	515	645	4.20	0.037	–	–	
MeCN	275, 293, 328, 383, 427, 509	510	575, 720	4.78, 0.30	0.024	–	–	
DMSO	279, 296, 331, 437, 476, 509	515	575, 735	5.79, 0.30	0.088	–	–	
Solid	–	540	666	5.60	0.029	–	–	
				**Ir16**				
H_2O	~325, 460, 525	–	549	0.003	0.54	–	–	35
DCM	–	–	583	–	–	–	–	
MeOH	–	–	559	–	–	–	–	
DMSO	–	–	561	–	–	–	–	
				**Ir17**				
DMSO	~330, 495	488	~580	–	–	–	–	48
DMF	~280, 325, 475	–	~590	–	–	–	–	
H_2O	470	–	528	–	–	–	–	
MeOH	~470	–	~585	–	–	–	–	

MeCN	~470	–	~580	–	–	–	–	
Ethylene glycol	~325, 490	–	~575	–	–	–	–	
Ethyl acetate	~300, 475, 490	–	~580	–	–	–	–	
DCM	467	–	587	1.34	0.74	–	–	
PBS	–	–	–	–	0.03	–	–	
**medium**	**λ_{abs} [nm]**	**λ_{exc} [nm]**	**λ_{PL} [nm]**	**τ [µs]**	**φ_{PL}**	**$E_{red,peak}$ [V] (ΔE [mV])/ $E_{red,onset}$ [V]/ $E_{1/2red}$[V]**	**$E_{ox,peak}$ [V]/$E_{ox,onset}$ [V] /$E_{1/2ox}$ [V]**	**Reference**
**Ir18**								
MeCN	~300, 325, 340, 506	–	–	–	–	–	–	252
DCM	557	–	–	–	–	–	–	
DMSO	499	–	–	–	–	–	–	
MeCN 0.1 M Bu_4NPF_6	–	–	–	–	–	$E_{1/2}$ = –0.76, –0.90	$E_{1/2}$ = 0.79	
**Ir19**								
MeCN	253, 281, 302sh, 451	–	–	–	–	–	–	252

Table S18. The absorption, emission and the relevant electrochemical data properties of Mn(I) complexes.

**medium**	**λ_{abs} [nm]**	**λ_{exc} [nm]**	**λ_{PL} [nm]**	**$E_{red, peak}$ [V] (ΔE [mV]) / $E_{1/2red}$ [V]**	**Reference**
**[MnBr(CO)$_3$(Ph-terpy)]**					
DMSO:PBS (0.5:99.5 v:v)	~425	–	–	–	253
MeCN	–	410	non emissive behavior	–	
**[MnBr(CO)$_3$(terpy)]**					
MeCN	302, 373sh, 409	–	–	–	254
MeCN 0.1 M Bu_4NPF_6	–	–	–	– 0.99, –1.56, –1.77	255
**Mn1**					
EtOH:H_2O (2:1 v:v)	208, 267, 311, 428	–	–	–	33
**Mn2**					
EtOH:H_2O (2:1 v:v)	209, 268, 309, 428	–	–	–	33
EtOH	209, 268, 311, 435	–	–	–	
H_2O	200, 307, 415	–	–	–	

Table S19. The absorption, emission and the relevant electrochemical data properties of model Re(I) complexes.

**medium**	**λ_{abs} [nm]**	**λ_{exc} [nm]**	**λ_{PL} [nm]**	**τ [ns]**	**φ_{PL}**	**$E_{red,peak}$ [V] (ΔE [mV])/ $E_{red,onset}$ [V]/ $E_{1/2red}$[V]**	**$E_{ox,peak}$ [V]/$E_{ox,onset}$ [V] /$E_{1/2ox}$ [V]**	**Reference**
**[ReCl(CO)$_3$(C_6H_4-terpy-κ^{2}N)]**								
$CHCl_3$	398	400	667	10.57	–	–	–	256
THF	400	400	676	8.72	0.002	–	–	
MeCN	375	380	666	6.13	0.002	–	–	
DMF	385	385	665	7.18	0.002	–	–	
EtOH:MeOH (4:1 v:v) 77 K	–	384	543	5070	–	–	–	
Solid	–	442	592	52.23	0.012	–	–	
MeCN	218, 260, 293, 324, 374	251, 300, 321, 380	666	6.13	0.0021	–	–	42
$CHCl_3$	264, 297, 397	266, 300, 400	667	10.57	0.0046	–	–	
solid	–	261, 302, 369, 442	592	52.23	0.0128	–	–	
EtOH:MeOH (4:1 v:v) 77 K	–	305, 333, 366, 384	543	5072	–	–	–	

film	388	310	370, 572	–	–	–	–	
		370	416, 438	–	0.0181	–	–	
		390	438, 572	–	0.0121	–	–	
MeCN 0.1 M Bu_4NPF_6	–	–	–	–	–	–1.73, –2.04	0.78, 0.91, 1.03	
DMF	380	–	652	–	–	–	–	257
2-MeTHF 77 K	–	–	570	1.53	–	–	–	
DMF 0.1 M Bu_4NPF_6	–	–	–	–	–	–1.73	–	
DMSO:H_2O (2:988 v:v)	~310, 385	380	~415, 435	–	–	–	–	258
**[ReCl(CO)$_3$(terpy-κ^{2}N)]**								
DMF:DCM (9:1 v:v) 77 K	–	–	530	3400	–	–	–	259
DMF Bu_4NClO_4	–	–	–	–	–	–1.40	–	
MeCN KPF_6	–	–	–	–	–	–	1.19	
DMF 0.1 M Bu_4NClO_4	–	–	–	–	–	–1.75 (60), –2.24^{irr}		260
MeCN	250–300, 320–400	360	506	–	–	–	–	261
MeCN 0.1 M Bu_4NPF_6	–	–	–	–	–	–1.741	0.786^{irr}	
DCM	220,295,378	442	509	2020	0.003	–	–	19
Solid	–	365	562	1950	–	–	–	
DMSO	~310,380	–	–	–	–	–	–	262
$CHCl_3$	–	265, 301, 322sh, 393	638	4.59	0.0042	–	–	62
MeCN	306, 323, 375	303, 323, 380	656	3.59	<0.0001	–	–	
Solid	–	491	582	600	–	–	–	
DCM 0.1 M Bu_4NPF_6	–	–	–	–	–	–1.71, –2.15	0.60, 0.85, 1.03	
DMSO:H_2O (1:9 v:v)	360	–	–	–	–	–	–	263
**[ReBr(CO)$_3$(C$_6$H$_4$-terpy-κ^{2}N)]**								
MeCN	218sh, 266, 328sh, 386	340	527, 653	14.92	0.001	–	–	264
DMF 0.1 M Bu_4NPF_6	–	–	–	–	–	–1.25(63), –1.74^{irr}	1.23^{irr}	
DMF	384	–	–	–	–	–	–	7
MeCN	266, 380	–	–	–	–	–	–	
MeCN 0.1 M Bu_4NBF_4	–	–	–	–	–	–1.70^{qr}, –2.04^{irr}	–	

**medium**	**λ$_{abs}$ [nm]**	**λ$_{exc}$ [nm]**	**λ$_{PL}$ [nm]**	**τ [ns]**	**φ$_{PL}$**	**E$_{red,peak}$ [V] (ΔE [mV])/ E$_{red,onset}$ [V]/ E$_{1/2red}$[V]**	**E$_{ox,peak}$ [V]/E$_{ox,onset}$ [V] /E$_{1/2ox}$ [V]**	**Reference**
**[ReBr(CO)$_3$(terpy-κ^{2}N)]**								
DCM 0.1 M Bu_4NPF_6	–	–	–	–	–	–1.35 (180)	1.41	263
MeCN	210sh, 247, 310, 322sh, 367	340	640	4.41	0.0003	–	–	
DMF 0.1 M Bu_4NClO_4	–	–	–	–	–	–1.30 (77)	1.31^{irr}	
DMF	309, 375	–	–	–	–	–	–	7
MeCN	247, 310, 372	–	–	–	–	–	–	
MeCN 0.1 M Bu_4NBF_4	–	–	–	–	–	–1.75^{qr}, –2.13^{irr}	–	
**[ReCl(CO)$_2$(C$_6$H$_4$-terpy-κ^{3}N)]**								
DMF 0.1 M Bu_4NPF_6	–	–	–	–	–	–1.65	0.05	265
DMF	407, 480, 718	–	–	–	–	–	–	
**[ReCl(CO)$_2$(terpy-κ^{3}N)]**								
DMF:DCM (9:1 v:v) 77 K	–	–	530	3400	–	–	–	259
DMF	375	–	–	–	–	–	–	
DMF Bu_4NClO_4	–	–	–	–	–	–1.40	–	
MeCN KPF_6	–	–	–	–	–	–	1.19	
MeCN	280, 320, 397, 460, 671	–	–	–	–	–	–	266
DCM	~280, 325, 405, 475, 750	–	–	–	–	–	–	
H_2O	~290, 320, 440, 600	–	–	–	–	–	–	
MeCN	239sh, 271, 280, 321, 398, 466, 567, 689	–	–	–	–	–	–	267
MeCN 0.1 M Bu_4NPF_6	–	–	–	–	–	–1.17^{irr}, –1.34^{irr}	0.48, 1.22^{irr}	
77 K	–	385	520	–	–	–	–	
77 K	–	325	522	–	–	–	–	
100 K	–		522	–	–	–	–	
125 K	–		548	–	–	–	–	
150 K	–		548	–	–	–	–	
DMSO	~280, 325, 395, 475, 675	–	–	–	–	–	–	262

Table S20. The absorption and emission data properties of Re(I) complexes.

medium	λ_{abs} [nm]	λ_{exc} [nm]	λ_{PL} [nm]	τ [ns]	φ_{PL}	Reference
**Re1**						
$CHCl_3$	247, 313, 380sh, 430	421	636	20.16	0.0113	268
MeCN	192, 246, 309, 354, 419	452	687	0.26	0.0191	
Solid	–	494	636	1349.75	0.0067	
EtOH:MeOH (4:1 v:v) 77 K	–	312, 325, 368, 444	580	227984.7	–	
DMF	425	–	532	380	–	257
2-MeTHF 77 K	–	–	560	–	–	
$CHCl_3$	430	425	637	20	0.013	256
THF	415	415	650	46.64	0.045	
MeCN	419	456	687	177.6	0.020	
DMF	425	425	680	1670	<0.001	
EtOH:MeOH (4:1 v:v) 77 K	–	444	580	227640	–	
Solid	–	494	636	1370	0.007	
**Re2**						
$CHCl_3$	405	405	645	8	0.009	256
THF	405	405	665	6	0.01	
MeCN	402	405	665	146	0.001	
DMF	409	409	650	248	<0.001	
EtOH:MeOH (4:1 v:v) 77 K	–	420	560	178000	–	
Solid	–	475	613	553	0.071	
**Re3**						
$5CHCl_3$	405	430	643	8	0.026	256
THF	406	445	665	6	0.027	
MeCN	405	415	640	79	0.003	
DMF	417	420	640	579	0.003	
EtOH:MeOH (4:1 v:v) 77 K	–	410	564	173000	–	
Solid	–	450	560, 589, 649	828	0.003	
**Re4**						
MeCN	420	456	695	14.6	0.004	269
$CHCl_3$	438	447	628	122.6	0.008	
film	463	320	369, 529	–	–	
Solid	–	515	604	2981.4	0.013	
EtOH:MeOH (4:1 v:v) 77 K	–	453	569, 601sh	240390	–	
**Re5**						
$CHCl_3$	300, 326, 371, 449	266, 301, 373, 452	613	44.85	0.0126	62
MeCN	295, 319, 359, 429	296, 323, 437	743	147.47	0.0892	
Film	310, 372, 448	375	418, 597	–	–	
EtOH:MeOH (4:1 v:v) 77 K	–	260, 296, 328, 372, 465	581, 626sh	293984	–	
Solid	–	447	586	4174.4	0.1008	
**Re6**						
MeCN	404	445	698	23	0.008	269
$CHCl_3$	418	358	510	4.1	0.01	
		417	634	16.60	0.008	
film	401	372	420, 442	–	–	
Solid	–	536	653	105.43	0.04	
EtOH:MeOH (4:1 v:v) 77 K	–	324	378, 531	–	–	
		435	585	228430	–	
**Re7**						
MeCN	398	420	661	37.72	0.01	269
$CHCl_3$	403	404	650	9.42	0.006	
film	418	323	374, 527	–	–	
Solid	–	470	571	5350	0.087	

EtOH:MeOH (4:1 v:v) 77 K	–	419	570	190230	–	
**medium**	**λ_{abs} [nm]**	**λ_{exc} [nm]**	**λ_{PL} [nm]**	**τ [ns]**	**φ_{PL}**	**Reference**
**Re8**						
DMSO	~385	–	–	–	–	270
DMF	~390	–	–	–	–	
MeCN	~385	–	–	–	–	
DCM	~400	–	–	–	–	
THF	~400	–	–	–	–	
1,4-dioxane	~400	–	–	–	–	
**Re9**						
DMSO	314, 413	–	–	–	–	270
DMF	~305, 409	–	–	–	–	
MeCN	~300, 400	–	–	–	–	
DCM	~300, 405	–	–	–	–	
THF	~300, 390	–	–	–	–	
1,4-dioxane	~300, 389	–	–	–	–	
Toluene	~300, 393	–	–	–	–	
**Re10**						
DMSO	~370	–	–	–	–	270
DMF	~370	–	–	–	–	
MeOH	~370	–	–	–	–	
DCM	~380	–	–	–	–	
THF	~380	–	–	–	–	
1,4-dioxane	~380	–	–	–	–	
**Re11**						
MeCN	191, 248, 308, 414	257, 315, 365, 458	702	0.079	0.0036	42
$CHCl_3$	247, 307, 421	289, 329, 404	507	3.74	0.0014	
		265, 303, 369, 432	617	40.15		
film	312, 401	330	384,575	–	0.0165	
		370	415, 438			
EtOH:MeOH (4:1 v:v) 77 K	–	319, 429	588	240930	–	
solid	–	302, 351, 522	625	2280	0.0129	
**Re12**						
DMF	406, 487, 694	–	–	–	–	265

Table S21. The relevant electrochemical data properties of Re(I) complexes.

**medium**	**$E_{red,peak}$ [V] (ΔE [mV])/ $E_{red,onset}$ [V]/ $E_{1/2red}$[V]**	**$E_{ox,peak}$ [V]/$E_{ox,onset}$ [V] /$E_{1/2ox}$ [V]**	**IP [eV]**	**EA [eV]**	**E_g [eV]**	**Reference**
**Re1**						
MeCN 0.1 M Bu_4NBF_4	–1.76	0.54	–5.64	–3.34	2.30	269
DMF 0.1 M Bu_4NPF_6	–1.81	–	–	–	–	257
**Re4**						
MeCN 0.1 M Bu_4NBF_4	–1.72	0.66	–5.76	–3.38	2.38	269
**Re5**						
DCM 0.1 M Bu_4NPF_6	$–1.70^{qr}$, –2,03	0.47^{qr}, 0.74, 0.95	–5.57	–3.40	2.17	62
**Re6**						
MeCN 0.1 M Bu_4NBF_4	–1.74	0.52, 0.63, 0.99	–5.62	–3.36	2.26	269
**Re7**						
MeCN 0.1 M Bu_4NBF_4	–1.75	0.64, 0.87	–5.74	–3.35	2.39	269
**Re11**						
MeCN 0.1 M Bu_4NPF_6	–1.84, –2.07, –2.23	0.58, 0.81	–5.56	–3.43	2.13	42
**Re12**						
DMF 0.1 M Bu_4NPF_6	–1.71	0.03	–	–	–	265

Table S22. The absorption, emission and the relevant electrochemical data properties of model Pt(II) complexes.

X^-	medium	λ_{abs} [nm]	λ_{exc} [nm]	λ_{PL} [nm]	τ [ns]	φ_{PL}	$E_{red,peak}$ [V] (ΔE [mV])/ $E_{red,onset}$ [V]/ $E_{1/2red}$[V]	$E_{ox,peak}$ [V]/$E_{ox,onset}$ [V] /$E_{1/2ox}$ [V]	Reference
**[PtCl(C_6H_4-terpy)]X**									
–	DCM	310, 323, 336, 390sh, 414	480	538, 572sh, 610sh	8.50	0.002	–	–	271
Cl	MeOH:EtOH (4:1) 77 K	284, 334, 410	337	515<br>650	15000<br>3700	–	–	–	101
			540	690	2600	–	–	–	
	DMF	405	–	–	–	–	–	–	272
	DMSO:H_2O (3:1 v:v)	–	367	415, 435, 505	6.58	0.009	–	–	
CF_3SO_3	DMSO	~340, 380, 410	–	–	–	–	–	–	64
**[PtCl(terpy)]X**									
–	DCM	305, 320, 340, 388, 405	–	–	–	–	–	–	271
Cl	MeOH:EtOH (4:1 v:v)	284, 334, 404sh	–	–	–	–	–	–	101
	MeOH:EtOH (4:1v:v) 77 K	–	337	470<br>670	15000<br>3100	–	–	–	
			540	730	2000	–	–	–	
**[Pt(C≡CC_6H_5){C_6H_4-terpy}]Cl**									
Cl	10% DMSO-DMEM	~300, 350, 450	–	–	–	–	–	–	273
**[Pt(C≡CC_6H_5){terpy}]X**									
CF_3SO_3	DMSO	263, 290, 313, 333, 348, 436	–	–	–	–	–	–	274
PF_6	EtOH:MeOH:DMF (10:10:1 v:v:v) 77 K	–	–	534, 570	–	–	–	–	275
	MeCN	244, 262, 269sh, 284sh, 312, 328, 343, 432	–	–	–	–	–	–	
	DMF 0.1 M Bu_4NOH	–	–	–	–	–	$E_{1/2}$ = –0.77 (88), –1.30 (67)	–	
	H_2O	~340	–	–	–	–	–	–	276
	DMF	~260, 290, 345, 440	–	566	–	–	–	–	277
	MeCN	261, 285, 303, 326, 343, 361, 384	–	–	–	–	–	–	278
	solid	–	–	–	–	–	–	–	
	DMF:MeOH:EtOH (1:1:1 v:v:v) 77 K	–	–	–	–	–	–	–	
	DCM	251, 262, 287, 306, 330, 344, 361, 421	–	–	–	–	–	–	
	Acetone	344, 362, 397	–	–	–	–	–	–	
	THF	287, 303, 330, 345, 363, 401	–	–	–	–	–	–	
	DMF	287, 302, 332, 347, 365, 395	–	–	–	–	–	–	
	solid	–	500	765	162	–	–	–	279
	Solid 77 K	–	580	805	–	–	–	–	
	DMF	314, 333, 347, 440	–	622	–	–	–	–	280
	2-MTHF	455	–	620	–	–	–	–	
	2-MTHF 77 K	–	–	593	–	–	–	–	
	MeCN	272, 284, 312, 328, 342, 434		630	–	–	–	–	281
	DMF:MeOH:EtOH (1:1:4 v:v:v) 77 K	–	–	530, 571, 618	–	–	–	–	
	Solid	–	–	800	<100	–	–	–	
	Solid 77 K	–	–	830	700	–	–	–	
	MeCN	272, 286, 312, 328, 342, 432	–	630	500	0.0124	–	–	282
	Solid	–	–	800	<100	–	–	–	
	Solid 77 K	–	–	830	700	–	–	–	

	BuCN 77 K	–	–	530	13300	–	–	–	
	MeCN 0.1 M Bu_4NPF_6	–	–	–	–	–	–0.97	1.22	
OTf	tris/HCl buffer (pH 7.6)/MeOH (v/v ¼1/14)	~390, 440	–	~600	–	–	–	–	283
	MeOH:MeCN (2:1 v:v)	~310, 330, 350, 440	–	–	–	–	–	–	284
TFA	DCM	~250, 325, 350, 480	480	~615	–	–	–	–	285
ClO_4	MeCN:H_2O (2:3 v:v)	430	–	597	1900	0.01	–	–	286
	DCM	287, 318, 329, 345, 422, 477	–	618	1900	0.036	–	–	287
	MeCN	432	–	–	–	–	–	–	288
SbF_6	MeCN	244, 259, 300, 350, 430	–	608	–	–	–	–	289
	DCM	–	–	619	–	–	–	–	

Table S23. The absorption, emission data properties of Pt(II) complexes.

medium	λ_{abs} [nm]	λ_{exc} [nm]	λ_{PL} [nm]	τ [µs]	φ_{PL}	Reference
**Pt1**						
DCM	255, 284, 330, 345sh, 514	409	536	4.48	0.063	290
MeCN	260, 281, 324, 338sh, 484	384	588	3.18	0.014	
MeOH:EtOH 77 K	–	531	660sh, 734	19680	–	
solid	325, 524	–	–	–	–	
**Pt2**						
MeCN:H_2O (1:1 v:v)	288, 312, 326, 340, 504	475	677	–	–	291
**Pt3**						
MeCN:H_2O (1:1 v:v)	311, 326, 340, 509	475	689	–	–	291
**Pt4**						
MeCN:H_2O (1:1 v:v)	–	–	518	–	–	291
**Pt5**						
MeCN	282, 328, 449	–	–	–	–	292
**Pt6**						
TBS buffer	~260, 290, 320, 340, 520	–	–	–	–	293

Table S24. The relevant electrochemical data properties of Pt(II) complexes.

medium	$E_{red,peak}$ [V] (ΔE [mV])/ $E_{red,onset}$ [V]/ $E_{1/2red}$[V]	$E_{ox,peak}$ [V]/$E_{ox,onset}$ [V] /$E_{1/2ox}$ [V]	IP [eV]	EA [eV]	E_g [eV]	Reference
**Pt1**						
MeCN 0.01 M Bu_4NPF_6	–1.22, –1.84/–1.11, –1.75	0.45, 0.73/0.32, 0.60	–5.42	–3.99	1.43	290
**Pt5**						
MeCN 0.1 M Bu_4NClO_4	–	1.08	–	–	–	292

References

(1) Groom, C. R.; Bruno, I. J.; Lightfoot, M. P.; Ward, S. C. The Cambridge Structural Database. *Acta Cryst B* **2016**, *72* (2), 171–179. https://doi.org/10.1107/S2052520616003954.

(2) Mutai, T.; Cheon, J.-D.; Arita, S.; Araki, K. Phenyl-Substituted 2,2′:6′,2″-Terpyridine as a New Series of Fluorescent Compounds—Their Photophysical Properties and Fluorescence Tuning. *J. Chem. Soc., Perkin Trans. 2* **2001**, No. 7, 1045–1050. https://doi.org/10.1039/B102685M.

(3) Goodall, W.; Wild, K.; Arm, K. J.; Williams, J. A. G. The Synthesis of 4′-Aryl Substituted Terpyridines by Suzuki Cross-Coupling Reactions: Substituent Effects on Ligand Fluorescence. *J. Chem. Soc., Perkin Trans. 2* **2002**, No. 10, 1669–1681. https://doi.org/10.1039/B205330F.

(4) Emmerling, F.; Bricks, J. L.; Resch-Genger, U.; Kraus, W.; Schulz, B.; Li, Y. Q.; Reck, G. Influence of the Donor Substituent and Acceptor Alkylation on the Structure–Analytical Properties of Mono- and Bifunctional Biphenyl-Type Fluorescent Reporters. *Journal of Molecular Structure* **2008**, *874* (1), 14–27. https://doi.org/10.1016/j.molstruc.2007.03.025.

(5) Maroń, A. M.; Cannelli, O.; Socie, E. C.; Lodowski, P.; Machura, B. Push-Pull Effect of Terpyridine Substituted by Triphenylamine Motive—Impact of Viscosity, Polarity and Protonation on Molecular Optical Properties. *Molecules* **2022**, *27* (20), 7071. https://doi.org/10.3390/molecules27207071.

(6) Li, L.; Bi, D.; Du, X.; Xing, X.; Cheng, X.; Feng, Y.; Wang, H.; Zhao, Q.; Qiu, D. Ternary Electrofluorochromic Behavior of the Terpyridine Eu(III) Complex with a Bis-Arylamine Redox Group. *Inorganic Chemistry Communications* **2022**, *146*, 110052. https://doi.org/10.1016/j.inoche.2022.110052.
(7) Saha, S.; Doughty, T.; Banerjee, D.; Patel, S. K.; Mallick, D.; Iyer, E. S. S.; Roy, S.; Mitra, R. Electrocatalytic Reduction of CO2 to CO by a Series of Organometallic Re(I)-Tpy Complexes. *Dalton Trans.* **2023**, *52* (42), 15394–15411. https://doi.org/10.1039/D3DT00441D.
(8) Liu, J.-H.; Tu, T.; Shen, Y.-L.; Tu, B.; Qian, D.-J. Interfacial Self-Assembly of Organized Ultrathin Films of Tripodal Metal-Terpyridyl Coordination Polymers as Luminophores and Heterogeneous Catalysts for Photocatalytic CO2 Reduction. *Langmuir* **2023**, *39* (13), 4777–4788. https://doi.org/10.1021/acs.langmuir.3c00166.
(9) Ilmi, R.; Wang, J.; Dutra, J. D. L.; Zhou, L.; Wong, W.-Y.; Raithby, P. R.; Khan, M. S. Efficient Red Organic Light Emitting Diodes of Nona Coordinate Europium Tris(β-Diketonato) Complexes Bearing 4′-Phenyl-2,2′:6′,2″-Terpyridine. *Chemistry – A European Journal* **2023**, *29* (37), e202300376. https://doi.org/10.1002/chem.202300376.
(10) Fu, F.; Liu, D.; Zhao, L.; Li, H.; Bai, X.; Chen, M.; Jiang, Z.; Su, P.; Zhong, W.; Li, Y.; Liao, W.; He, J.; Wang, P. Substituents Make a Difference: 6,6″-Modified Terpyridine Complexes with Helix Configuration and Enhanced Emission. *Dalton Trans.* **2023**, *52* (10), 3033–3039. https://doi.org/10.1039/D2DT04006A.
(11) Najafi, E.; Janghouri, M.; Hashemzadeh, A.; Ng, S. W. Mixed Ligand Cd(II) Coordination Architectures Based on Bulky Anthracene-9-Carboxylate Ligand: Crystal Structures and Optical Properties. *Inorganica Chimica Acta* **2023**, *552*, 121482. https://doi.org/10.1016/j.ica.2023.121482.
(12) Zhao, Q.; Li, L.; Bi, D.; Wang, H.; Liu, D.; Wei, Y.; Xing, X.; Yang, C.; Qiu, D.; Zhou, G. Manipulating Intramolecular Charge Transfer in Terpyridine Derivatives Towards "Turn-On" Fluorescence Chemosensors for Zn2+. *Chemistry – An Asian Journal* **2025**, *20* (2), e202401247. https://doi.org/10.1002/asia.202401247.
(13) Alencar, F. M. S. de; Gouveia, F. S.; Oliveira, G. de F. S. de; Andrade, A. L.; Vasconcelos, M. A. de; Ayala, A. P.; Gondim, A. C. S.; Carvalho, I. M. M. de; Moraes, C. A. F.; Palmeira-Mello, M. V.; Batista, A. A.; Lopes, L. G. de F.; Sousa, E. H. S. Terpyridine-Based Ruthenium Complexes Containing a 4,5-Diazafluoren-9-One Ligand with Light-Driven Enhancement of Biological Activity. *Dalton Trans.* **2025**, *54* (5), 1850–1870. https://doi.org/10.1039/D4DT02562H.
(14) Sedykh, A. E.; Kurth, D. G.; Müller-Buschbaum, K. Two Series of Lanthanide Coordination Polymers and Complexes with 4′-Phenylterpyridine and Their Luminescence Properties. *European Journal of Inorganic Chemistry* **2019**, *2019* (42), 4564–4571. https://doi.org/10.1002/ejic.201900872.
(15) Cai, L. L.; Hu, Y. T.; Li, Y.; Wang, K.; Zhang, X. Q.; Muller, G.; Li, X. M.; Wang, G. X. Solid-State Luminescence Properties, Hirshfeld Surface Analysis and DFT Calculations of Mononuclear Lanthanide Complexes (Ln = EuIII, GdIII, TbIII, DyIII) Containing 4′-Phenyl-2,2′:6′,2″-Terpyridine. *Inorganica Chimica Acta* **2019**, *489*, 85–92. https://doi.org/10.1016/j.ica.2019.02.001.
(16) Munzert, S. M.; Schwarz, G.; Kurth, D. G. Kinetic Studies of the Coordination of Mono- and Ditopic Ligands with First Row Transition Metal Ions. *Inorg. Chem.* **2016**, *55* (5), 2565–2573. https://doi.org/10.1021/acs.inorgchem.5b02931.
(17) Sedykh, A. E.; Kurth, D. G.; Müller-Buschbaum, K. Phosphorescence Afterglow and Thermal Properties of [ScCl3(Ptpy)] (Ptpy: 4'-Phenyl-2,2',6',2"-Terpyridine). *Zeitschrift für anorganische und allgemeine Chemie* **2021**, *647* (4), 359–364. https://doi.org/10.1002/zaac.202000347.
(18) Patel, M. N.; Gandhi, D. S.; Parmar, P. A. Effect of Substituent of Terpyridines on the DNA-Interaction of Polypyridyl Ruthenium(II) Complexes. *Spectrochimica Acta Part A: Molecular and Biomolecular Spectroscopy* **2011**, *84* (1), 243–248. https://doi.org/10.1016/j.saa.2011.09.037.
(19) Wang, D.; Xu, Q.-L.; Zhang, S.; Li, H.-Y.; Wang, C.-C.; Li, T.-Y.; Jing, Y.-M.; Huang, W.; Zheng, Y.-X.; Accorsi, G. Synthesis and Photoluminescence Properties of Rhenium(I) Complexes Based on 2,2′:6′,2″-Terpyridine Derivatives with Hole-Transporting Units. *Dalton Trans.* **2013**, *42* (8), 2716–2723. https://doi.org/10.1039/C2DT32154H.
(20) Rai, S.; Padhi, S. K. Effectual Electrocatalytic Proton and Water Reduction by CuII Terpyridine Scaffolds. *Electrochimica Acta* **2020**, *364*, 137277. https://doi.org/10.1016/j.electacta.2020.137277.
(21) Fajardo, D. A.; Arteaga, D.; Ellena, J.; Santiago, P. H. O.; D'Vries, R. F.; Lenis, L. A. Synthesis, Characterization and Structural Analysis of Com-plexes from 2,2′:6′,2″-Terpyridine Derivatives with Transition Metals. *Acta Cryst C* **2024**, *80* (6), 200–211. https://doi.org/10.1107/S2053229624004224.
(22) Wang, X.; Li, D.; Dai, J.; Xue, Q.; Yang, C.; Xia, L.; Qi, X.; Bao, B.; Yang, S.; Xu, Y.; Yuan, C.; Luo, W.; Cabot, A.; Dai, L. Blocking Metal Nanocluster Growth through Ligand Coordination and Subsequent Polymerization: The Case of Ruthenium Nanoclusters as Robust Hydrogen Evolution Electrocatalysts. *Small* **2024**, *20* (22), 2309176. https://doi.org/10.1002/smll.202309176.
(23) Naik, S.; Kumar, S.; Mague, J. T.; Balakrishna, M. S. A Hybrid Terpyridine-Based Bis(Diphenylphosphino)Amine Ligand, Terpy-C6H4N(PPh2)2: Synthesis, Coordination Chemistry and

Photoluminescence Studies. *Dalton Trans.* **2016**, *45* (46), 18434–18437. https://doi.org/10.1039/C6DT03440C.
(24) Storrier, G. D.; Colbran, S. B.; Craig, D. C. Bis[4′-(4-Anilino)-2,2′:6′,2″-Terpyridine]Transition-Metal Complexes: Electrochemically Activemonomers with a Range of Magnetic and Optical Properties for Assembly Ofmetallo Oligomers and Macromolecules. *J. Chem. Soc., Dalton Trans.* **1997**, No. 17, 3011–3028. https://doi.org/10.1039/A702778H.
(25) Palion-Gazda, J.; Machura, B.; Klemens, T.; Szlapa-Kula, A.; Krompiec, S.; Siwy, M.; Janeczek, H.; Schab-Balcerzak, E.; Grzelak, J.; Maćkowski, S. Structure-Dependent and Environment-Responsive Optical Properties of the Trisheterocyclic Systems with Electron Donating Amino Groups. *Dyes and Pigments* **2019**, *166*, 283–300. https://doi.org/10.1016/j.dyepig.2019.03.035.
(26) CHAO, D. Highly Selective Detection of Zn2+ and Cd2+ with a Simple Amino-Terpyridine Compound in Solution and Solid State. *J Chem Sci* **2016**, *128* (1), 133–139. https://doi.org/10.1007/s12039-015-1011-y.
(27) Maroń, A.; Szlapa, A.; Klemens, T.; Kula, S.; Machura, B.; Krompiec, S.; Małecki, J. G.; Świtlicka-Olszewska, A.; Erfurt, K.; Chrobok, A. Tuning the Photophysical Properties of 4′-Substituted Terpyridines – an Experimental and Theoretical Study. *Org. Biomol. Chem.* **2016**, *14* (15), 3793–3808. https://doi.org/10.1039/C6OB00038J.
(28) Bi, X.; Pang, Y. Optical Response of Terpyridine Ligands to Zinc Binding: A Close Look at the Substitution Effect by Spectroscopic Studies at Low Temperature. *J. Phys. Chem. B* **2016**, *120* (13), 3311–3317. https://doi.org/10.1021/acs.jpcb.6b00515.
(29) Goodall, W.; Williams, J. A. G. A New, Highly Fluorescent Terpyridine Which Responds to Zinc Ions with a Large Red-Shift in Emission. *Chem. Commun.* **2001**, No. 23, 2514–2515. https://doi.org/10.1039/B108408A.
(30) Sarkar, P.; Das, D.; Sutradhar, S.; Ghosh, B. N. Selective Sensing of Sulphide Ion by a Simple Mercury (II) Complex of an Amino-Substituted Terpyridine in Aqueous Solution. *Journal of Molecular Structure* **2024**, *1301*, 137392. https://doi.org/10.1016/j.molstruc.2023.137392.
(31) Sasaki, I.; Amabilino, S.; Mallet-Ladeira, S.; Tassé, M.; Sournia-Saquet, A.; Lacroix, P. G.; Malfant, I. Further Studies on the Photoreactivities of Ruthenium–Nitrosyl Complexes with Terpyridyl Ligands. *New J. Chem.* **2019**, *43* (28), 11241–11250. https://doi.org/10.1039/C9NJ02398D.
(32) Shi, P.; Jiang, Q.; Zhao, X.; Zhang, Q.; Tian, Y. Study of the One-Photon and Two-Photon Properties of Two Water-Soluble Terpyridines and Their Zinc Complexes. *Dalton Trans.* **2015**, *44* (17), 8041–8048. https://doi.org/10.1039/C5DT00449G.
(33) Jiang, Q.; Xia, Y.; Barrett, J.; Mikhailovsky, A.; Wu, G.; Wang, D.; Shi, P.; Ford, P. C. Near-Infrared and Visible Photoactivation to Uncage Carbon Monoxide from an Aqueous-Soluble PhotoCORM. *Inorg. Chem.* **2019**, *58* (16), 11066–11075. https://doi.org/10.1021/acs.inorgchem.9b01581.
(34) Yang, X.; Zhang, D.; Li, J.; Ji, W.; Yang, N.; Gu, S.; Wu, Q.; Jiang, Q.; Shi, P.; Li, L. A Mitochondrion-Targeting Mn(II)-Terpyridine Complex for Two-Photon Photodynamic Therapy. *Chem. Commun.* **2020**, *56* (63), 9032–9035. https://doi.org/10.1039/D0CC02051F.
(35) Yu, Q.; Gu, S.; Yang, X.; Jiang, Q.; Shi, P. Four Cyclometalated Ir(III) Complexes and Insights into Their Luminescence, Cytotoxicity and DNA/BSA Binding Performance. *RSC Adv.* **2024**, *14* (41), 29934–29941. https://doi.org/10.1039/D4RA04408H.
(36) Zhang, L.; Ma, S.; Wang, T.; Li, S.; Wang, L.; Li, D.; Tian, Y.; Zhang, Q. Four-Photon Absorption Iron Complex for Magnetic Resonance/Photoacoustic Dual-Model Imaging and an Enhanced Ferroptosis Process. *Anal. Chem.* **2023**, *95* (2), 1635–1642. https://doi.org/10.1021/acs.analchem.2c04763.
(37) Feng, Z.; Zhu, T.; Wang, L.; Yuan, T.; Jiang, Y.; Tian, X.; Tian, Y.; Zhang, Q. Coordination-Regulated Terpyridine–Mn(II) Complexes for Photodynamic Therapy Guided by Multiphoton Fluorescence/Magnetic Resonance Imaging. *Inorg. Chem.* **2022**, *61* (32), 12652–12661. https://doi.org/10.1021/acs.inorgchem.2c01603.
(38) Tessore, F.; Roberto, D.; Ugo, R.; Pizzotti, M.; Quici, S.; Cavazzini, M.; Bruni, S.; De Angelis, F. Terpyridine Zn(II), Ru(III), and Ir(III) Complexes: The Relevant Role of the Nature of the Metal Ion and of the Ancillary Ligands on the Second-Order Nonlinear Response of Terpyridines Carrying Electron Donor or Electron Acceptor Groups. *Inorg. Chem.* **2005**, *44* (24), 8967–8978. https://doi.org/10.1021/ic050975q.
(39) Righetto, S.; Rondena, S.; Locatelli, D.; Roberto, D.; Tessore, F.; Ugo, R.; Quici, S.; Roma, S.; Korystov, D.; Srdanov, V. I. An Investigation on the Two-Photon Absorption Activity of Various Terpyridines and Related Homoleptic and Heteroleptic Cationic Zn(II) Complexes. *J. Mater. Chem.* **2006**, *16* (15), 1439–1444. https://doi.org/10.1039/B515129E.
(40) Angelis, F. D.; Fantacci, S.; Sgamelotti, A.; Cariati, F.; Roberto, D.; Tessore, F.; Ugo, R. A Time-Dependent Density Functional Theory Investigation on the Nature of the Electronic Transitions Involved in the Nonlinear Optical Response of [Ru(CF3CO2)3T] (T = 4′-(C6H4-p-NBu2)-2,2′:6′,2″-Terpyridine). *Dalton Trans.* **2006**, No. 6, 852–859. https://doi.org/10.1039/B509123C.

(41) Roberto, D.; Tessore, F.; Ugo, R.; Bruni, S.; Manfredi, A.; Quici, S. Terpyridine Zn(II), Ru(III) and Ir(III) Complexes as New Asymmetric Chromophores for Nonlinear Optics: First Evidence for a Shift from Positive to Negative Value of the Quadratic Hyperpolarizability of a Ligand Carrying an Electron Donor Substituent upon Coordination to Different Metal Centres. *Chem. Commun.* **2002**, No. 8, 846–847. https://doi.org/10.1039/B201221A.
(42) Klemens, T.; Świtlicka, A.; Szlapa-Kula, A.; Krompiec, S.; Lodowski, P.; Chrobok, A.; Godlewska, M.; Kotowicz, S.; Siwy, M.; Bednarczyk, K.; Libera, M.; Maćkowski, S.; Pędziński, T.; Schab-Balcerzak, E.; Machura, B. Experimental and Computational Exploration of Photophysical and Electroluminescent Properties of Modified 2,2′:6′,2″-Terpyridine, 2,6-Di(Thiazol-2-Yl)Pyridine and 2,6-Di(Pyrazin-2-Yl)Pyridine Ligands and Their Re(I) Complexes. *Applied Organometallic Chemistry* **2018**, *32* (12), e4611. https://doi.org/10.1002/aoc.4611.
(43) *Near-IR Electrochromic Film with High Optical Contrast and Stability Prepared by Oxidative Electropolymerization of Triphenylamine Modified Terpyridine Platinum(II) Chloride*. https://www.mdpi.com/1420-3049/28/24/8027 (accessed 2025-03-12).
(44) Sivadas, D. K.; Gayathri, P.; Ravi, S.; Karthikeyan, S.; Pannipara, M.; Al-Sehemi, A. G.; Moon, D.; Anthony, S. P.; Madhu, V. Distinct Fluorescence State, Mechanofluorochromism of Terpyridine Conjugated Fluorophores and the Reusable Sensing of Nitroaromatics in Aqueous Medium. *New J. Chem.* **2023**, *47* (27), 12770–12778. https://doi.org/10.1039/D3NJ01235B.
(45) Ma, Y.; Liu, S.; Yang, H.; Zeng, Y.; She, P.; Zhu, N.; Ho, C.-L.; Zhao, Q.; Huang, W.; Wong, W.-Y. Luminescence Color Tuning by Regulating Electrostatic Interaction in Light-Emitting Devices and Two-Photon Excited Information Decryption. *Inorg. Chem.* **2017**, *56* (5), 2409–2416. https://doi.org/10.1021/acs.inorgchem.6b02319.
(46) Tsukamoto, T.; Aoki, R.; Sakamoto, R.; Toyoda, R.; Shimada, M.; Hattori, Y.; Asaoka, M.; Kitagawa, Y.; Nishibori, E.; Nakano, M.; Nishihara, H. A Simple Zinc(II) Complex That Features Multi-Functional Luminochromism Induced by Reversible Ligand Dissociation. *Chem. Commun.* **2017**, *53* (26), 3657–3660. https://doi.org/10.1039/C6CC10190A.
(47) Tang, K.-C.; Liu, K. L.; Chen, I.-C. Rapid Intersystem Crossing in Highly Phosphorescent Iridium Complexes. *Chemical Physics Letters* **2004**, *386* (4), 437–441. https://doi.org/10.1016/j.cplett.2004.01.098.
(48) Fan, C.; Ye, C.; Wang, X.; Chen, Z.; Zhou, Y.; Liang, Z.; Tao, X. Synthesis and Electrochromic Properties of New Terpyridine–Triphenylamine Hybrid Polymers. *Macromolecules* **2015**, *48* (18), 6465–6473. https://doi.org/10.1021/acs.macromol.5b00493.
(49) Liu, J.; Zhang, Q.; Ding, H.; Zhang, J.; Tan, J.; Wang, C.; Wu, J.; Li, S.; Zhou, H.; Yang, J.; Tian, Y. Two Novel Terpyridine-Based Chromophores with Donor-Acceptor Structural Model Containing Modified Triphenylamine Moiety: Synthesis, Crystal Structures and Two-Photon Absorption Properties. *Sci. China Chem.* **2013**, *56* (9), 1315–1324. https://doi.org/10.1007/s11426-013-4940-7.
(50) Kröhnke Pyridine Synthesis. In *Name Reactions: A Collection of Detailed Reaction Mechanisms*; Li, J. J., Ed.; Springer: Berlin, Heidelberg, 2006; pp 343–344. https://doi.org/10.1007/3-540-30031-7_152.
(51) Xiao, L.; Xu, Y.; Yan, M.; Galipeau, D.; Peng, X.; Yan, X. Excitation-Dependent Fluorescence of Triphenylamine-Substituted Tridentate Pyridyl Ruthenium Complexes. *J. Phys. Chem. A* **2010**, *114* (34), 9090–9097. https://doi.org/10.1021/jp1040234.
(52) Robson, K. C. D.; Koivisto, B. D.; Gordon, T. J.; Baumgartner, T.; Berlinguette, C. P. Triphenylamine-Modified Ruthenium(II) Terpyridine Complexes: Enhancement of Light Absorption by Conjugated Bridging Motifs. *Inorg. Chem.* **2010**, *49* (12), 5335–5337. https://doi.org/10.1021/ic9025427.
(53) Bozic-Weber, B.; Constable, E. C.; Hostettler, N.; Housecroft, C. E.; Schmitt, R.; Schönhofer, E. The D10 Route to Dye-Sensitized Solar Cells: Step-Wise Assembly of Zinc(II) Photosensitizers on TiO2 Surfaces. *Chem. Commun.* **2012**, *48* (46), 5727–5729. https://doi.org/10.1039/C2CC31729J.
(54) Liu, B.; Zhang, Q.; Ding, H.; Hu, G.; Du, Y.; Wang, C.; Wu, J.; Li, S.; Zhou, H.; Yang, J.; Tian, Y. Synthesis, Crystal Structures and Two-Photon Absorption Properties of a Series of Terpyridine-Based Chromophores. *Dyes and Pigments* **2012**, *95* (1), 149–160. https://doi.org/10.1016/j.dyepig.2012.03.020.
(55) Liu, J.; Zhu, Y.; Tian, X.; Li, F.; Xu, W.; Zhang, Y.; Wang, C.; Zhang, J.; Zhou, H.; Wu, J.; Tian, Y. Synthesis, Crystal Structures of a Series of Novel 2, 2′:6′, 2″-Terpyridine Derivatives: The Influences of Substituents on Their Photophysical Properties and Intracellular Acid Organelle Targeting. *Dyes and Pigments* **2016**, *128*, 149–157. https://doi.org/10.1016/j.dyepig.2015.10.040.
(56) Song, P.; Sun, S.-G.; Liu, J.-Y.; Xu, Y.-Q.; Han, K.-L.; Peng, X.-J. Theoretical and Experimental Study on the Intramolecular Charge Transfer Excited State of the New Highly Fluorescent Terpyridine Compound. *Spectrochimica Acta Part A: Molecular and Biomolecular Spectroscopy* **2009**, *74* (3), 753–757. https://doi.org/10.1016/j.saa.2009.08.010.
(57) Sun, M.-J.; Shao, J.-Y.; Yao, C.-J.; Zhong, Y.-W.; Yao, J. Osmium Bisterpyridine Complexes with Redox-Active Amine Substituents: A Comparison Study with Ruthenium Analogues. *Inorg. Chem.* **2015**, *54* (16), 8136–8147. https://doi.org/10.1021/acs.inorgchem.5b01420.

(58) Tang, Y.; Kong, M.; Tian, X.; Wang, J.; Xie, Q.; Wang, A.; Zhang, Q.; Zhou, H.; Wu, J.; Tian, Y. A Series of Terpyridine-Based Zinc(II) Complexes Assembled for Third-Order Nonlinear Optical Responses in the near-Infrared Region and Recognizing Lipid Membranes. *J. Mater. Chem. B* **2017**, *5* (31), 6348–6355. https://doi.org/10.1039/C7TB01063J.
(59) Liu, H.; Tang, Y.; Li, F.; Kong, M.; Zhao, M.; Yang, J.; Li, S.; Wu, J.; Tian, Y. A Series of CdIIX2 (X = Cl, Br, I) Complexes with D-A Model and Their Third-Order Nonlinear Optical Properties with a Femtosecond Laser in the near IR Region. *Journal of Coordination Chemistry* **2017**, *70* (6), 960–972. https://doi.org/10.1080/00958972.2017.1280157.
(60) Kong, M.; Zhu, Y.; Du, W.; Zhang, Q.; Tian, X.; Li, S.; Zhou, H.; Wu, J.; Tian, Y. A Series of Terpyridine Derivatives for Aggregation-Induced Emission, Two-Photon Absorption and Mitochondrial Targeting. *Dyes and Pigments* **2018**, *158*, 225–232. https://doi.org/10.1016/j.dyepig.2018.05.051.
(61) Deng, H.; Wang, T.; Chen, Y.; Dou, K.; Liu, X.; Zhao, C.; Zhan, H.; Yang, C.; Qin, C.; Cheng, Y. Enhanced Thermally Activated Delayed Fluorescence by Sole Coordination: From an Organic Molecule to Its Zinc Complex. *J. Phys. Chem. Lett.* **2024**, *15* (27), 7003–7010. https://doi.org/10.1021/acs.jpclett.4c01472.
(62) Maroń, A. M.; Szlapa-Kula, A.; Matussek, M.; Kruszynski, R.; Siwy, M.; Janeczek, H.; Grzelak, J.; Maćkowski, S.; Schab-Balcerzak, E.; Machura, B. Photoluminescence Enhancement of Re(I) Carbonyl Complexes Bearing D–A and D–π–A Ligands. *Dalton Trans.* **2020**, *49* (14), 4441–4453. https://doi.org/10.1039/C9DT04871E.
(63) Yang, J.; Liu, B.; Wu, J.; Zhang, S.; Jin, B.; Tian, Y. Sensitive ctDNA Detection by a Novel Zinc(II) Complex with Two-Photon Absorption Based on Electrochemiluminescence. *New J. Chem.* **2015**, *39* (2), 1404–1409. https://doi.org/10.1039/C4NJ01727G.
(64) Canil, G.; Braccini, S.; Marzo, T.; Marchetti, L.; Pratesi, A.; Biver, T.; Funaioli, T.; Chiellini, F.; Hoeschele, J. D.; Gabbiani, C. Photocytotoxic Pt(IV) Complexes as Prospective Anticancer Agents. *Dalton Trans.* **2019**, *48* (29), 10933–10944. https://doi.org/10.1039/C9DT01645G.
(65) Nie, H.-J.; Yao, C.-J.; Sun, M.-J.; Zhong, Y.-W.; Yao, J. Ruthenium-Bis-Terpyridine Complex with Two Redox-Asymmetric Amine Substituents: Potential-Controlled Reversal of the Direction of Charge-Transfer. *Organometallics* **2014**, *33* (21), 6223–6231. https://doi.org/10.1021/om500904k.
(66) Liu, D.; Zhang, M.; Du, W.; Hu, L.; Li, F.; Tian, X.; Wang, A.; Zhang, Q.; Zhang, Z.; Wu, J.; Tian, Y. A Series of Zn(II) Terpyridine-Based Nitrate Complexes as Two-Photon Fluorescent Probe for Identifying Apoptotic and Living Cells via Subcellular Immigration. *Inorg. Chem.* **2018**, *57* (13), 7676–7683. https://doi.org/10.1021/acs.inorgchem.8b00620.
(67) Duan, L.; Zheng, Q.; Liang, Y.; Tu, T. From Simple Probe to Smart Composites: Water-Soluble Pincer Complex With Multi-Stimuli-Responsive Luminescent Behaviors. *Advanced Materials* **2024**, *36* (46), 2409620. https://doi.org/10.1002/adma.202409620.
(68) Chen, G.-J.; Wang, Z.-G.; Kou, Y.-Y.; Tian, J.-L.; Yan, S.-P. Impact of Metal on the DNA Photo-Induced Cleavage Activity of a Family of Phterpy Complexes. *Journal of Inorganic Biochemistry* **2013**, *122*, 49–56. https://doi.org/10.1016/j.jinorgbio.2013.01.010.
(69) Zhang, Y.; Zhou, P.; Liang, B.; Huang, L.; Zhou, Y.; Ma, Z. Effects of Counterions of Colorful Sandwich-Type Zinc(II) 4′-Phenyl-Terpyridine Compounds on Photoluminescent and Thermal Properties. *Journal of Molecular Structure* **2017**, *1146*, 504–511. https://doi.org/10.1016/j.molstruc.2017.05.129.
(70) Maity, B.; Gadadhar, S.; Goswami, T. K.; Karande, A. A.; Chakravarty, A. R. Impact of Metal on the DNA Photocleavage Activity and Cytotoxicity of Ferrocenyl Terpyridine 3d Metal Complexes. *Dalton Trans.* **2011**, *40* (44), 11904–11913. https://doi.org/10.1039/C1DT11102G.
(71) Li, Y. Q.; Bricks, J. L.; Resch-Genger, U.; Spieles, M.; Rettig, W. Bifunctional Charge Transfer Operated Fluorescent Probes with Acceptor and Donor Receptors. 2. Bifunctional Cation Coordination Behavior of Biphenyl-Type Sensor Molecules Incorporating 2,2‘:6‘,2‘ ‘-Terpyridine Acceptors. *J. Phys. Chem. A* **2006**, *110* (38), 10972–10984. https://doi.org/10.1021/jp062013q.
(72) Jacques, A.; Lebrun, C.; Casini, A.; Kieffer, I.; Proux, O.; Latour, J.-M.; Sénèque, O. Reactivity of Cys4 Zinc Finger Domains with Gold(III) Complexes: Insights into the Formation of “Gold Fingers.” *Inorg. Chem.* **2015**, *54* (8), 4104–4113. https://doi.org/10.1021/acs.inorgchem.5b00360.
(73) Zhang, K.; Meng, X.; He, L. Cationic Zinc(II) Complexes with Carbazole-Type Counter-Anions: Intracomplex Donor/Acceptor Pairs Affording Exciplexes with Thermally Activated Delayed Fluorescence. *Inorg. Chem.* **2023**, *62* (5), 2135–2145. https://doi.org/10.1021/acs.inorgchem.2c03804.
(74) Albano, G.; Balzani, V.; Constable, E. C.; Maestri, M.; Smith, D. R. Photoinduced Processes in 4′-(9-Anthryl)-2,2′:6′,2″-Terpyridine, Its Protonated Forms and Zn(II), Ru(II) and Os(II) Complexes. *Inorganica Chimica Acta* **1998**, *277* (2), 225–231. https://doi.org/10.1016/S0020-1693(97)06159-8.
(75) Collin, J.-P.; Dixon, I. M.; Sauvage, J.-P.; Williams, J. A. G.; Barigelletti, F.; Flamigni, L. Synthesis and Photophysical Properties of Iridium(III) Bisterpyridine and Its Homologues: A Family of Complexes with a Long-Lived Excited State. *J. Am. Chem. Soc.* **1999**, *121* (21), 5009–5016. https://doi.org/10.1021/ja9833669.

(76) Wang, H.; Cai, F.; Feng, D.; Zhou, L.; Li, D.; Wei, Y.; Feng, Z.; Zhang, J.; He, J.; Wu, Y. Synthesis, Crystal Structure, Photophysical Property and Bioimaging Application of a Series of Zn(II) Terpyridine Complexes. *Journal of Molecular Structure* **2019**, *1194*, 157–162. https://doi.org/10.1016/j.molstruc.2019.05.090.

(77) Tsukamoto, T.; Aoki, R.; Sakamoto, R.; Toyoda, R.; Shimada, M.; Hattori, Y.; Kitagawa, Y.; Nishibori, E.; Nakano, M.; Nishihara, H. Mechano-, Thermo-, Solvato-, and Vapochromism in Bis(Acetato-κ1O)[4′-(4-(Diphenylamino)Phenyl)](2,2′:6′,2″-Terpyridine-κ3N,N′,N″)Zinc(II) and Its Polymer. *Chem. Commun.* **2017**, *53* (70), 9805–9808. https://doi.org/10.1039/C7CC05022D.

(78) Duan, L.; Zheng, Q.; Tu, T. Instantaneous High-Resolution Visual Imaging of Latent Fingerprints in Water Using Color-Tunable AIE Pincer Complexes. *Advanced Materials* **2022**, *34* (35), 2202540. https://doi.org/10.1002/adma.202202540.

(79) Tsukamoto, T.; Takada, K.; Sakamoto, R.; Matsuoka, R.; Toyoda, R.; Maeda, H.; Yagi, T.; Nishikawa, M.; Shinjo, N.; Amano, S.; Iokawa, T.; Ishibashi, N.; Oi, T.; Kanayama, K.; Kinugawa, R.; Koda, Y.; Komura, T.; Nakajima, S.; Fukuyama, R.; Fuse, N.; Mizui, M.; Miyasaki, M.; Yamashita, Y.; Yamada, K.; Zhang, W.; Han, R.; Liu, W.; Tsubomura, T.; Nishihara, H. Coordination Nanosheets Based on Terpyridine–Zinc(II) Complexes: As Photoactive Host Materials. *J. Am. Chem. Soc.* **2017**, *139* (15), 5359–5366. https://doi.org/10.1021/jacs.6b12810.

(80) Hsu, T.-W.; Hsu, H.-C.; Chan, H.-Y.; Fang, J.-M. A Terpyridine Zinc Complex for Selective Detection of Lipid Pyrophosphates: A Model System for Monitoring Bacterial O- and N-Transglycosylations. *J. Org. Chem.* **2020**, *85* (19), 12747–12753. https://doi.org/10.1021/acs.joc.0c01252.

(81) Deda, M. L.; Maio, G. D.; Candreva, A.; Heinrich, B.; Andelescu, A.-A.; Popa, E.; Voirin, E.; Badea, V.; Amati, M.; Costişor, O.; Donnio, B.; Szerb, E. I. Very Intense Polarized Emission in Self-Assembled Room Temperature Metallomesogens Based on Zn(II) Coordination Complexes: An Experimental and Computational Study. *J. Mater. Chem. C* **2021**, *10* (1), 115–125. https://doi.org/10.1039/D1TC05059A.

(82) Gasnier, A.; Barbe, J.-M.; Bucher, C.; Duboc, C.; Moutet, J.-C.; Saint-Aman, E.; Terech, P.; Royal, G. Soluble Heterometallic Coordination Polymers Based on a Bis-Terpyridine-Functionalized Dioxocyclam Ligand. *Inorg. Chem.* **2010**, *49* (6), 2592–2599. https://doi.org/10.1021/ic901832q.

(83) Basu, U.; Khan, I.; Koley, D.; Saha, S.; Kondaiah, P.; Chakravarty, A. R. Nuclear Targeting Terpyridine Iron(II) Complexes for Cellular Imaging and Remarkable Photocytotoxicity. *Journal of Inorganic Biochemistry* **2012**, *116*, 77–87. https://doi.org/10.1016/j.jinorgbio.2012.06.006.

(84) Yang, J.; Clegg, J. K.; Jiang, Q.; Lui, X.; Yan, H.; Zhong, W.; Beves, J. E. Multi-Pyridine Decorated Fe(II) and Ru(II) Complexes by Pd(0)-Catalysed Cross Couplings: New Building Blocks for Metallosupramolecular Assemblies. *Dalton Trans.* **2013**, *42* (44), 15625–15636. https://doi.org/10.1039/C3DT52331D.

(85) Mehr, H. S.; Romano, N. C.; Altamimi, R.; Modarelli, J. M.; Modarelli, D. A. Core Substituted Naphthalene Diimide – Metallo Bisterpyridine Supramolecular Polymers: Synthesis, Photophysics and Morphology. *Dalton Trans.* **2015**, *44* (7), 3176–3184. https://doi.org/10.1039/C4DT02719A.

(86) Wang, Y.; Liu, T.; Chen, L.; Chao, D. Water-Assisted Highly Efficient Photocatalytic Reduction of CO2 to CO with Noble Metal-Free Bis(Terpyridine)Iron(II) Complexes and an Organic Photosensitizer. *Inorg. Chem.* **2021**, *60* (8), 5590–5597. https://doi.org/10.1021/acs.inorgchem.0c03503.

(87) Rodríguez-Jiménez, S.; Song, H.; Lam, E.; Wright, D.; Pannwitz, A.; Bonke, S. A.; Baumberg, J. J.; Bonnet, S.; Hammarström, L.; Reisner, E. Self-Assembled Liposomes Enhance Electron Transfer for Efficient Photocatalytic CO2 Reduction. *J. Am. Chem. Soc.* **2022**, *144* (21), 9399–9412. https://doi.org/10.1021/jacs.2c01725.

(88) Papp, M.; Keszthelyi, T.; Vancza, A.; Bajnóczi, É. G.; Kováts, É.; Németh, Z.; Bogdán, C.; Bazsó, G.; Rozgonyi, T.; Vankó, G. Molecular Engineering to Tune Functionality: The Case of Cl-Substituted [Fe(Terpy)2]2+. *Inorg. Chem.* **2023**, *62* (16), 6397–6410. https://doi.org/10.1021/acs.inorgchem.3c00271.

(89) Curiac, C.; Rodrigues, R. R.; Watson, J.; Hunt, L. A.; Devdass, A.; Jurss, J. W.; Hammer, N. I.; Fortenberry, R. C.; Delcamp, J. H. Iron Redox Shuttles with Wide Optical Gap Dyes for High-Voltage Dye-Sensitized Solar Cells. *ChemSusChem* **2021**, *14* (15), 3084–3096. https://doi.org/10.1002/cssc.202100884.

(90) Zimmer, P.; Burkhardt, L.; Friedrich, A.; Steube, J.; Neuba, A.; Schepper, R.; Müller, P.; Flörke, U.; Huber, M.; Lochbrunner, S.; Bauer, M. The Connection between NHC Ligand Count and Photophysical Properties in Fe(II) Photosensitizers: An Experimental Study. *Inorg. Chem.* **2018**, *57* (1), 360–373. https://doi.org/10.1021/acs.inorgchem.7b02624.

(91) Britz, A.; Gawelda, W.; Assefa, T. A.; Jamula, L. L.; Yarranton, J. T.; Galler, A.; Khakhulin, D.; Diez, M.; Harder, M.; Doumy, G.; March, A. M.; Bajnóczi, É.; Németh, Z.; Pápai, M.; Rozsályi, E.; Sárosiné Szemes, D.; Cho, H.; Mukherjee, S.; Liu, C.; Kim, T. K.; Schoenlein, R. W.; Southworth, S. H.; Young, L.; Jakubikova, E.; Huse, N.; Vankó, G.; Bressler, C.; McCusker, J. K. Using Ultrafast X-Ray Spectroscopy To Address Questions in Ligand-Field Theory: The Excited State Spin and Structure of [Fe(Dcpp)2]2+. *Inorg. Chem.* **2019**, *58* (14), 9341–9350. https://doi.org/10.1021/acs.inorgchem.9b01063.

(92) Steube, J.; Burkhardt, L.; Päpcke, A.; Moll, J.; Zimmer, P.; Schoch, R.; Wölper, C.; Heinze, K.; Lochbrunner, S.; Bauer, M. Excited-State Kinetics of an Air-Stable Cyclometalated Iron(II) Complex. *Chemistry – A European Journal* **2019**, *25* (51), 11826–11830. https://doi.org/10.1002/chem.201902488.
(93) Carey, M. C.; Adelman, S. L.; McCusker, J. K. Insights into the Excited State Dynamics of Fe(II) Polypyridyl Complexes from Variable-Temperature Ultrafast Spectroscopy. *Chem. Sci.* **2018**, *10* (1), 134–144. https://doi.org/10.1039/C8SC04025G.
(94) Shepard, S. G.; Fatur, S. M.; Rappé, A. K.; Damrauer, N. H. Highly Strained Iron(II) Polypyridines: Exploiting the Quintet Manifold To Extend the Lifetime of MLCT Excited States. *J. Am. Chem. Soc.* **2016**, *138* (9), 2949–2952. https://doi.org/10.1021/jacs.5b13524.
(95) Cook, L. J. K.; Tuna, F.; Halcrow, M. A. Iron(II) and Cobalt(II) Complexes of Tris-Azinyl Analogues of 2,2′:6′,2″-Terpyridine. *Dalton Trans.* **2013**, *42* (6), 2254–2265. https://doi.org/10.1039/C2DT31736B.
(96) Fatur, S. M.; Shepard, S. G.; Higgins, R. F.; Shores, M. P.; Damrauer, N. H. A Synthetically Tunable System To Control MLCT Excited-State Lifetimes and Spin States in Iron(II) Polypyridines. *J. Am. Chem. Soc.* **2017**, *139* (12), 4493–4505. https://doi.org/10.1021/jacs.7b00700.
(97) Duchanois, T.; Etienne, T.; Beley, M.; Assfeld, X.; Perpète, E. A.; Monari, A.; Gros, P. C. Heteroleptic Pyridyl-Carbene Iron Complexes with Tuneable Electronic Properties. *European Journal of Inorganic Chemistry* **2014**, *2014* (23), 3747–3753. https://doi.org/10.1002/ejic.201402356.
(98) Brown, A. M.; McCusker, C. E.; McCusker, J. K. Spectroelectrochemical Identification of Charge-Transfer Excited States in Transition Metal-Based Polypyridyl Complexes. *Dalton Trans.* **2014**, *43* (47), 17635–17646. https://doi.org/10.1039/C4DT02849J.
(99) Jamula, L. L.; Brown, A. M.; Guo, D.; McCusker, J. K. Synthesis and Characterization of a High-Symmetry Ferrous Polypyridyl Complex: Approaching the 5T2/3T1 Crossing Point for FeII. *Inorg. Chem.* **2014**, *53* (1), 15–17. https://doi.org/10.1021/ic402407k.
(100) Constable, E. C.; Housecroft, C. E.; Neuburger, M.; Phillips, D.; Raithby, P. R.; Schofield, E.; Sparr, E.; Tocher, D. A.; Zehnder, M.; Zimmermann, Y. Development of Supramolecular Structure through Alkylation of Pendant Pyridyl Functionality. *J. Chem. Soc., Dalton Trans.* **2000**, No. 13, 2219–2228. https://doi.org/10.1039/B000940G.
(101) Arena, G.; Calogero, G.; Campagna, S.; Monsù Scolaro, L.; Ricevuto, V.; Romeo, R. Synthesis, Characterization, Absorption Spectra, and Luminescence Properties of Organometallic Platinum(II) Terpyridine Complexes. *Inorg. Chem.* **1998**, *37* (11), 2763–2769. https://doi.org/10.1021/ic9704896.
(102) Li, Y.; Huffman, J. C.; Flood, A. H. Can Terdentate 2,6-Bis(1,2,3-Triazol-4-Yl)Pyridines Form Stable Coordination Compounds? *Chem. Commun.* **2007**, No. 26, 2692–2694. https://doi.org/10.1039/B703301J.
(103) Kang, J.; Xu, B.; Peng, Z.; Zhu, X.; Wei, Y.; Powell, D. R. Molecular and Polymeric Hybrids Based on Covalently Linked Polyoxometalates and Transition-Metal Complexes. *Angewandte Chemie International Edition* **2005**, *44* (42), 6902–6905. https://doi.org/10.1002/anie.200501924.
(104) Chow, H. S.; Constable, E. C.; Housecroft, C. E.; Neuburger, M.; Schaffner, S. Ligands and Complexes with Supramolecular Aromatic–Aromatic Interactions: Iron(II) and Ruthenium(II) Complexes of 2,2′:6′,2″-Terpyridines with Pendant Naphthalene Groups. *Dalton Trans.* **2006**, No. 23, 2881–2890. https://doi.org/10.1039/B515610F.
(105) Chambers, J.; Eaves, B.; Parker, D.; Claxton, R.; Ray, P. S.; Slattery, S. J. Inductive Influence of 4′-Terpyridyl Substituents on Redox and Spin State Properties of Iron(II) and Cobalt(II) Bis-Terpyridyl Complexes. *Inorganica Chimica Acta* **2006**, *359* (8), 2400–2406. https://doi.org/10.1016/j.ica.2005.12.065.
(106) Yutaka, T.; Mori, I.; Kurihara, M.; Tamai, N.; Nishihara, H. Photochemical Behavior of Azobenzene-Conjugated CoII, CoIII, and FeII Bis(Terpyridine) Complexes. *Inorg. Chem.* **2003**, *42* (20), 6306–6313. https://doi.org/10.1021/ic0260466.
(107) Arana, C.; Yan, S.; Keshavarz-K., M.; Potts, K. T.; Abruna, H. D. Electrocatalytic Reduction of Carbon Dioxide with Iron, Cobalt, and Nickel Complexes of Terdentate Ligands. *Inorg. Chem.* **1992**, *31* (17), 3680–3682. https://doi.org/10.1021/ic00043a034.
(108) Sjödin, M.; Gätjens, J.; Tabares, L. C.; Thuéry, P.; Pecoraro, V. L.; Un, S. Tuning the Redox Properties of Manganese(II) and Its Implications to the Electrochemistry of Manganese and Iron Superoxide Dismutases. *Inorg. Chem.* **2008**, *47* (7), 2897–2908. https://doi.org/10.1021/ic702428s.
(109) M. Rao, J.; Hughes, M. C.; Macero, D. J. Voltammetry of Terpyridine and Terosine Complexes of Cobalt(II) and Iron(II). *Inorganica Chimica Acta* **1976**, *16*, 231–236. https://doi.org/10.1016/S0020-1693(00)91719-5.
(110) Drew, M. G. B.; Foreman, M. R. St. J.; Geist, A.; Hudson, M. J.; Marken, F.; Norman, V.; Weigl, M. Synthesis, Structure, and Redox States of Homoleptic d-Block Metal Complexes with Bis-1,2,4-Triazin-3-Yl-Pyridine and 1,2,4-Triazin-3-Yl-Bipyridine Extractants. *Polyhedron* **2006**, *25* (4), 888–900. https://doi.org/10.1016/j.poly.2005.09.030.

(111) Lin, H.-C.; Straus, D. A.; Johnson, V. A.; Lu, J. E.; Lopez, L.; Terrill, R. H. Preparation and Electrochemistry of a Pyrene-Linked Iron Terpyridine and Its Anodic Redox Polymer. *Electrochimica Acta* **2012**, *62*, 140–146. https://doi.org/10.1016/j.electacta.2011.12.002.

(112) Deng, Y.; Yin ,Hang; Zhao ,Zhiyong; Wang ,Ruibing; and Liu, S. A Study of Binding Interactions between Terpyridine Derivatives and Cucurbit[10]Uril. *Supramolecular Chemistry* **2018**, *30* (8), 706–712. https://doi.org/10.1080/10610278.2018.1455977.

(113) Naka, K.; Sato, H.; Fujita, T.; Iyi, N.; Yamagishi, A. Induction of Circular Dichroism by Coadsorption of Chiral and Achiral Metal Complexes on a Colloidal Clay. *J. Phys. Chem. B* **2003**, *107* (33), 8469–8473. https://doi.org/10.1021/jp022412v.

(114) Davidson, G. J. E.; Loeb, S. J. Iron(II) Complexes Utilising Terpyridine Containing [2]Rotaxanes as Ligands. *Dalton Trans.* **2003**, No. 22, 4319–4323. https://doi.org/10.1039/B308553H.

(115) Constable, E. C.; Thompson, A. M. W. C. Pendant-Functionalised Ligands for Metallosupramolecular Assemblies; Ruthenium(II) and Osmium(II) Complexes of 4′-(4-Pyridy1)-2,2′ : 6′,2″-Terpyridine. *J. Chem. Soc., Dalton Trans.* **1994**, No. 9, 1409–1418. https://doi.org/10.1039/DT9940001409.

(116) Robb, M. G.; Brooker, S. Incorporation of Switchable Inorganic Building Blocks into Heterometallic Coordination Polymers. *Crystal Growth & Design* **2023**, *23* (3), 1848–1859. https://doi.org/10.1021/acs.cgd.2c01341.

(117) Braterman, P. S.; Song, J. I.; Peacock, R. D. Electronic Absorption Spectra of the Iron(II) Complexes of 2,2’-Bipyridine, 2,2’-Bipyrimidine, 1,10-Phenanthroline, and 2,2’:6’,2’’-Terpyridine and Their Reduction Products. *Inorg. Chem.* **1992**, *31* (4), 555–559. https://doi.org/10.1021/ic00030a006.

(118) Jensen, P. W.; Jørgensen, L. B. Resonance Raman Spectra of Some Iron(II) Imine Complexes. *Journal of Molecular Structure* **1982**, *79*, 87–92. https://doi.org/10.1016/0022-2860(82)85035-7.

(119) Watanabe, I.; Ono, K.; Ikeda, S. Photoelectron Emission Study of Iron(II) and Cobalt(II) Complexes in Aqueous Solution. Reorganization Energies. *Bulletin of the Chemical Society of Japan* **1991**, *64* (2), 352–357. https://doi.org/10.1246/bcsj.64.352.

(120) Bellam, R.; Jaganyi, D. Substitution Kinetics of [Fe(PDT/PPDT)(Phen)]2+ (n ≠ m; n,m = 1,2) with 2,2′-Bipyridine, 1,10-Phenanthroline, and 2,2′,6,2″-Terpyridine. *International Journal of Chemical Kinetics* **2017**, *49* (3), 182–196. https://doi.org/10.1002/kin.21066.

(121) Zhang, J.; Campolo, D.; Dumur, F.; Xiao, P.; Fouassier, J. P.; Gigmes, D.; Lalevée, J. Visible-Light-Sensitive Photoredox Catalysis by Iron Complexes: Applications in Cationic and Radical Polymerization Reactions. *Journal of Polymer Science Part A: Polymer Chemistry* **2016**, *54* (14), 2247–2253. https://doi.org/10.1002/pola.28098.

(122) Mondal, P. C.; Manna, A. K. Synthesis of Heteroleptic Terpyridyl Complexes of Fe(II) and Ru(II): Optical and Electrochemical Studies. *New J. Chem.* **2016**, *40* (7), 5775–5781. https://doi.org/10.1039/C5NJ03106K.

(123) Alemán, E. A.; Shreiner, C. D.; Rajesh, C. S.; Smith, T.; Garrison, S. A.; Modarelli, D. A. Photoinduced Electron-Transfer within Osmium(II) and Ruthenium(II) Bis-Terpyridine Donor Acceptor Dyads. *Dalton Trans.* **2009**, No. 33, 6562–6577. https://doi.org/10.1039/B903130H.

(124) Maestri, M.; Armaroli, N.; Balzani, V.; Constable, E. C.; Thompson, A. M. W. C. Complexes of the Ruthenium(II)-2,2’:6’,2’’-Terpyridine Family. Effect of Electron-Accepting and -Donating Substituents on the Photophysical and Electrochemical Properties. *Inorg. Chem.* **1995**, *34* (10), 2759–2767. https://doi.org/10.1021/ic00114a039.

(125) Thummel, R. P.; Hegde, V.; Jahng, Y. Influence of Remote Substituents on the Properties of Bis(Terpyridyl)Ruthenium(II) Complexes. *Inorg. Chem.* **1989**, *28* (16), 3264–3267. https://doi.org/10.1021/ic00315a037.

(126) Agnew, S. F.; Stone, M. L.; Crosby, G. A. Emission Spectra, Decay Times, and Polarization Ratios of Ruthenium(II) Complexes Containing Tridentate Ligands. *Chemical Physics Letters* **1982**, *85* (1), 57–60. https://doi.org/10.1016/0009-2614(82)83460-X.

(127) Stone, M. L.; Crosby, G. A. Charge-Transfer Luminescence from Ruthenium(II) Complexes Containing Tridentate Ligands. *Chemical Physics Letters* **1981**, *79* (1), 169–173. https://doi.org/10.1016/0009-2614(81)85312-2.

(128) Sasaki, Y.; Yanai, N.; Kimizuka, N. Osmium Complex–Chromophore Conjugates with Both Singlet-to-Triplet Absorption and Long Triplet Lifetime through Tuning of the Heavy-Atom Effect. *Inorg. Chem.* **2022**, *61* (16), 5982–5990. https://doi.org/10.1021/acs.inorgchem.1c03129.

(129) Benavides, P. A.; Matias, T. A.; Araki, K. Unexpected Lability of the [RuIII(Phtpy)Cl3] Complex. *Dalton Trans.* **2017**, *46* (44), 15567–15572. https://doi.org/10.1039/C7DT03658B.

(130) Koizumi, T.; Tanaka, K. Synthesis, Chemical- and Electrochemical Properties of Ruthenium(II) Complexes Bearing 2,6-Bis(2-Naphthyridyl)Pyridine. *Inorganica Chimica Acta* **2005**, *358* (6), 1999–2004. https://doi.org/10.1016/j.ica.2004.12.008.

(131) López, R.; Villagra, D.; Ferraudi, G.; Moya, S. A.; Guerrero, J. Preparation and Photophysical Properties of Precursors of Inorganic Macromolecules. Mono and Binuclear Complexes of Ru(II) and Terpyridine Derivatized with Thiophene and 4′-(5-Bromothiophene) Groups. *Inorganica Chimica Acta* **2004**, *357* (12), 3525–3531. https://doi.org/10.1016/j.ica.2004.02.038.

(132) Amouyal, E.; Bahout, M.; Calzaferri, G. *Excited states of M(II,d6)-4'-phenylterpyridine complexes: electron localization*. ACS Publications. https://doi.org/10.1021/j100173a017.

(133) Hissler, M.; El-ghayoury, A.; Harriman, A.; Ziessel, R. Fine-Tuning the Electronic Properties of Binuclear Bis(Terpyridyl)Ruthenium(II) Complexes. *Angewandte Chemie International Edition* **1998**, *37* (12), 1717–1720. https://doi.org/10.1002/(SICI)1521-3773(19980703)37:12<1717::AID-ANIE1717>3.0.CO;2-T.

(134) Chen, W.; Rein, F. N.; Rocha, R. C. Homogeneous Photocatalytic Oxidation of Alcohols by a Chromophore–Catalyst Dyad of Ruthenium Complexes. *Angewandte Chemie International Edition* **2009**, *48* (51), 9672–9675. https://doi.org/10.1002/anie.200904756.

(135) Awada, A.; Moreno-Betancourt, A.; Philouze, C.; Moreau, Y.; Jouvenot, D.; Loiseau, F. New Acridine-Based Tridentate Ligand for Ruthenium(II): Coordination with a Twist. *Inorg. Chem.* **2018**, *57* (24), 15430–15437. https://doi.org/10.1021/acs.inorgchem.8b02735.

(136) Ziegler, M.; Monney, V.; Stoeckli-Evans, H.; Zelewsky, A. V.; Sasaki, I.; Dupic, G.; Daran, J.-C.; Balavoine, G. G. A. Complexes of New Chiral Terpyridyl Ligands. Synthesis and Characterization of Their Ruthenium(II) and Rhodium(III) Complexes. *J. Chem. Soc., Dalton Trans.* **1999**, No. 5, 667–676. https://doi.org/10.1039/A900194H.

(137) Constable, E. C.; Harverson, P.; E.Housecroft, C. Convergent Syntheses of Topologically Linear Heterotri- and Heteropenta-Nuclear Complexes Based upon Bis{4′-(2,2′:6′,2″-Terpyridinyl)} Ether. *J. Chem. Soc., Dalton Trans.* **1999**, No. 21, 3693–3700. https://doi.org/10.1039/A906419B.

(138) Benniston, A. C.; Grosshenny, V.; Harriman, A.; Ziessel, R. Photophysical Properties of Closely-Coupled, Binuclear Ruthenium(II) Bis(2,2′:6′,2″-Terpyridine) Complexes. *Dalton Trans.* **2004**, No. 8, 1227–1232. https://doi.org/10.1039/B400931B.

(139) Peterson, J. R.; Smith, T. A.; Thordarson, P. Synthesis and Room Temperature Photo-Induced Electron Transfer in Biologically Active Bis(Terpyridine)Ruthenium(II)–Cytochrome c Bioconjugates and the Effect of Solvents on the Bioconjugation of Cytochrome c. *Org. Biomol. Chem.* **2009**, *8* (1), 151–162. https://doi.org/10.1039/B919289A.

(140) Bomben, P. G.; Robson, K. C. D.; Sedach, P. A.; Berlinguette, C. P. On the Viability of Cyclometalated Ru(II) Complexes for Light-Harvesting Applications. *Inorg. Chem.* **2009**, *48* (20), 9631–9643. https://doi.org/10.1021/ic900653q.

(141) Cui, B.-B.; Shao, J.-Y.; Zhong, Y.-W. Bis-Tridentate Ruthenium Complexes with a Redox-Active Amine Substituent: Electrochemical, Spectroscopic, and DFT/TDDFT Studies. *Organometallics* **2014**, *33* (16), 4220–4229. https://doi.org/10.1021/om500486v.

(142) Chao, D.; Fu, W.-F. Facile Synthesis of a Ruthenium Assembly and Its Application for Light-Driven Oxidation of Alcohols in Water. *Chem. Commun.* **2013**, *49* (37), 3872–3874. https://doi.org/10.1039/C3CC00305A.

(143) Muratsugu, S.; Nishihara, H. π-Conjugation Modification of Photochromic and Redox-Active Dimethyldihydropyrene by Phenyl- and Ethynyl-Terpyridines and Ru(Bis-Terpyridine) Complexes. *New J. Chem.* **2014**, *38* (12), 6114–6124. https://doi.org/10.1039/C4NJ01462F.

(144) Bolink, H. J.; Cappelli, L.; Coronado, E.; Gaviña, P. Observation of Electroluminescence at Room Temperature from a Ruthenium(II) Bis-Terpyridine Complex and Its Use for Preparing Light-Emitting Electrochemical Cells. *Inorg. Chem.* **2005**, *44* (17), 5966–5968. https://doi.org/10.1021/ic0507058.

(145) Harriman, A.; Ziessel, R. Making Photoactive Molecular-Scale Wires. *Chem. Commun.* **1996**, No. 15, 1707–1716. https://doi.org/10.1039/CC9960001707.

(146) Cerfontaine, S.; Marcélis, L.; Laramee-Milette, B.; Hanan, G. S.; Loiseau, F.; De Winter, J.; Gerbaux, P.; Elias, B. Converging Energy Transfer in Polynuclear Ru(II) Multiterpyridine Complexes: Significant Enhancement of Luminescent Properties. *Inorg. Chem.* **2018**, *57* (5), 2639–2653. https://doi.org/10.1021/acs.inorgchem.7b03040.

(147) Son, S. U.; Park, K. H.; Lee, Y.-S.; Kim, B. Y.; Choi, C. H.; Lah, M. S.; Jang, Y. H.; Jang, D.-J.; Chung, Y. K. Synthesis of Ru(II) Complexes of N-Heterocyclic Carbenes and Their Promising Photoluminescence Properties in Water. *Inorg. Chem.* **2004**, *43* (22), 6896–6898. https://doi.org/10.1021/ic049514f.

(148) Constable, E. C.; Ward, M. D. Synthesis and Co-Ordination Behaviour of 6′,6″-Bis(2-Pyridyl)-2,2′ : 4,4″ : 2″,2‴-Quaterpyridine; 'Back-to-Back' 2,2′ : 6′,2″-Terpyridine. *J. Chem. Soc., Dalton Trans.* **1990**, No. 4, 1405–1409. https://doi.org/10.1039/DT9900001405.

(149) Grosshenny, V.; Harriman, A.; Hissler, M.; Ziessel, R. Intramolecular Triplet Energy Transfer in Alkyne-Bridged Ru–Os Multinuclear Complexes: Switching between Dipole–Dipole and Electron-Exchange

Mechanisms. *J. Chem. Soc., Faraday Trans.* **1996**, *92* (12), 2223–2238. https://doi.org/10.1039/FT9969202223.
(150) Farlow, B.; Nile, T. A.; Walsh, J. L.; McPhail, A. T. Synthesis, x-Ray Structural Determination and Coordination Chemistry of 4′-Ferrocenyl-2,2′:6′,2″-Terpyridine. *Polyhedron* **1993**, *12* (23), 2891–2894. https://doi.org/10.1016/S0277-5387(00)80074-1.
(151) Hecker, C. R.; Gushurst, Ann. K. I.; McMillin, D. R. Phenyl Substituents and Excited-State Lifetimes in Ruthenium(II) Terpyridyls. *Inorg. Chem.* **1991**, *30* (3), 538–541. https://doi.org/10.1021/ic00003a037.
(152) Hjelm, J.; Handel, R. W.; Hagfeldt, A.; Constable, E. C.; Housecroft, C. E.; Forster, R. J. Conducting Polymers Containing In-Chain Metal Centers: Electropolymerization of Oligothienyl-Substituted {M(Tpy)2} Complexes and in Situ Conductivity Studies, M = Os(II), Ru(II). *Inorg. Chem.* **2005**, *44* (4), 1073–1081. https://doi.org/10.1021/ic049221m.
(153) Morris, D. E.; Hanck, K. W.; DeArmond, M. K. ESR Studies of the Redox Orbitals in Diimine Complexes of Iron(II) and Ruthenium(II). *J. Am. Chem. Soc.* **1983**, *105* (10), 3032–3038. https://doi.org/10.1021/ja00348a017.
(154) El-ghayoury, A.; Harriman, A.; Khatyr, A.; Ziessel, R. Intramolecular Triplet Energy Transfer in Metal Polypyridine Complexes Bearing Ethynylated Aromatic Groups. *J. Phys. Chem. A* **2000**, *104* (7), 1512–1523. https://doi.org/10.1021/jp9930447.
(155) Wadman, S. H.; Lutz, M.; Tooke, D. M.; Spek, A. L.; Hartl, F.; Havenith, R. W. A.; van Klink, G. P. M.; van Koten, G. Consequences of N,C,N′- and C,N,N′-Coordination Modes on Electronic and Photophysical Properties of Cyclometalated Aryl Ruthenium(II) Complexes. *Inorg. Chem.* **2009**, *48* (5), 1887–1900. https://doi.org/10.1021/ic801595m.
(156) Glazier, S.; Barron, J. A.; Houston, P. L.; Abruña, H. D. Photophysics of PAMAM-Based Dendrimers of Polypyridyl Complexes of Ruthenium. *J. Phys. Chem. B* **2002**, *106* (39), 9993–10003. https://doi.org/10.1021/jp013312c.
(157) Flores-Torres, S.; Hutchison, G. R.; Soltzberg, L. J.; Abruña, H. D. Ruthenium Molecular Wires with Conjugated Bridging Ligands: Onset of Band Formation in Linear Inorganic Conjugated Oligomers. *J. Am. Chem. Soc.* **2006**, *128* (5), 1513–1522. https://doi.org/10.1021/ja0552139.
(158) Norrby, T.; Börje, A.; Åkermark, B.; Hammarström, L.; Alsins, J.; Lashgari, K.; Norrestam, R.; Mårtensson, J.; Stenhagen, G. Synthesis, Structure, and Photophysical Properties of Novel Ruthenium(II) Carboxypyridine Type Complexes. *Inorg. Chem.* **1997**, *36* (25), 5850–5858. https://doi.org/10.1021/ic9705812.
(159) Koizumi, T.; Tomon, T.; Tanaka, K. Terpyridine-Analogous (N,N,C)-Tridentate Ligands: Synthesis, Structures, and Electrochemical Properties of Ruthenium(II) Complexes Bearing Tridentate Pyridinium and Pyridinylidene Ligands. *Organometallics* **2003**, *22* (5), 970–975. https://doi.org/10.1021/om020637m.
(160) Jameson, D. L.; Blaho, J. K.; Kruger, K. T.; Goldsby, K. A. Redox Regulation in Ruthenium(II) Complexes of 2,6-Bis(N-Pyrazolyl)Pyridine Ligands: Synthetically Versatile Analogs of 2,2':6',2''-Terpyridine. *Inorg. Chem.* **1989**, *28* (24), 4312–4314. https://doi.org/10.1021/ic00323a005.
(161) Liu, Y.; Hammitt, R.; Lutterman, D. A.; Joyce, L. E.; Thummel, R. P.; Turro, C. Ru(II) Complexes of New Tridentate Ligands: Unexpected High Yield of Sensitized 1O2. *Inorg. Chem.* **2009**, *48* (1), 375–385. https://doi.org/10.1021/ic801636u.
(162) Hutchison, K.; Morris, J. C.; Nile, T. A.; Walsh, J. L.; Thompson, D. W.; Petersen, J. D.; Schoonover, J. R. Spectroscopic and Photophysical Properties of Complexes of 4‘-Ferrocenyl-2,2‘:6‘,2‘ ‘-Terpyridine and Related Ligands. *Inorg. Chem.* **1999**, *38* (10), 2516–2523. https://doi.org/10.1021/ic980798b.
(163) Koivisto, B. D.; Robson, K. C. D.; Berlinguette, C. P. Systematic Manipulation of the Light-Harvesting Properties for Tridentate Cyclometalated Ruthenium(II) Complexes. *Inorg. Chem.* **2009**, *48* (20), 9644–9652. https://doi.org/10.1021/ic9007137.
(164) Robson, K. C. D.; Koivisto, B. D.; Yella, A.; Sporinova, B.; Nazeeruddin, M. K.; Baumgartner, T.; Grätzel, M.; Berlinguette, C. P. Design and Development of Functionalized Cyclometalated Ruthenium Chromophores for Light-Harvesting Applications. *Inorg. Chem.* **2011**, *50* (12), 5494–5508. https://doi.org/10.1021/ic200011m.
(165) Jakubikova, E.; Chen, W.; Dattelbaum, D. M.; Rein, F. N.; Rocha, R. C.; Martin, R. L.; Batista, E. R. Electronic Structure and Spectroscopy of [Ru(Tpy)2]2+, [Ru(Tpy)(Bpy)(H2O)]2+, and [Ru(Tpy)(Bpy)(Cl)]+. *Inorg. Chem.* **2009**, *48* (22), 10720–10725. https://doi.org/10.1021/ic901477m.
(166) Thompson, D. W.; Fleming, C. N.; Myron, B. D.; Meyer, T. J. Rigid Medium Stabilization of Metal-to-Ligand Charge Transfer Excited States. *J. Phys. Chem. B* **2007**, *111* (24), 6930–6941. https://doi.org/10.1021/jp068682l.
(167) Thummel, R. P.; Chirayil, S. Ruthenium(II) Complexes of Tetra-2-Pyridyl-1,4-Diazine. *Inorganica Chimica Acta* **1988**, *154* (1), 77–81. https://doi.org/10.1016/S0020-1693(00)85168-3.

(168) Sun, Y.; El Ojaimi, M.; Hammitt, R.; Thummel, R. P.; Turro, C. Effect of Ligands with Extended π-System on the Photophysical Properties of Ru(II) Complexes. *J. Phys. Chem. B* **2010**, *114* (45), 14664–14670. https://doi.org/10.1021/jp102613n.
(169) Zong, R.; Naud, F.; Segal, C.; Burke, J.; Wu, F.; Thummel, R. Design and Study of Bi[1,8]Naphthyridine Ligands as Potential Photooxidation Mediators in Ru(II) Polypyridyl Aquo Complexes. *Inorg. Chem.* **2004**, *43* (20), 6195–6202. https://doi.org/10.1021/ic040051n.
(170) Hung, C.-Y.; Wang, T.-L.; Jang, Y.; Kim, W. Y.; Schmehl, R. H.; Thummel, R. P. Dipyrido[4,3-b;5,6-b]Acridine Derivatives and Their Ruthenium(II) Complexes. *Inorg. Chem.* **1996**, *35* (20), 5953–5956. https://doi.org/10.1021/ic960409i.
(171) Zong, R.; Wang, B.; Thummel, R. P. Trans-[RuII(Dpp)Cl2]: A Convenient Reagent for the Preparation of Heteroleptic Ru(Dpp) Complexes, Where Dpp Is 2,9-Di(Pyrid-2′-Yl)-1,10-Phenanthroline. *Inorg. Chem.* **2012**, *51* (5), 3179–3185. https://doi.org/10.1021/ic202648h.
(172) Yang, W.-W.; Wang, L.; Zhong, Y.-W.; Yao, J. Tridentate Cyclometalated Ruthenium(II) Complexes of "Click" Ligand 1,3-Di(1,2,3-Triazol-4-Yl)Benzene. *Organometallics* **2011**, *30* (8), 2236–2240. https://doi.org/10.1021/om200039j.
(173) Yao, C.-J.; Yao, J.; Zhong, Y.-W. Electronic Communication between Two Amine Redox Centers Bridged by a Bis(Terpyridine)Ruthenium(II) Complex. *Inorg. Chem.* **2011**, *50* (15), 6847–6849. https://doi.org/10.1021/ic200701j.
(174) Yang, W.-W.; Zhong, Y.-W.; Yoshikawa, S.; Shao, J.-Y.; Masaoka, S.; Sakai, K.; Yao, J.; Haga, M. Tuning of Redox Potentials by Introducing a Cyclometalated Bond to Bis-Tridentate Ruthenium(II) Complexes Bearing Bis(N-Methylbenzimidazolyl)Benzene or -Pyridine Ligands. *Inorg. Chem.* **2012**, *51* (2), 890–899. https://doi.org/10.1021/ic2016885.
(175) Barbieri, A.; Ventura, B.; Barigelletti, F.; De Nicola, A.; Quesada, M.; Ziessel, R. Mononuclear and Binuclear Wirelike Ruthenium(II) Complexes with Oligo-Diethynyl-Thiophene Bridged Back-to-Back Terpyridine Ligands: Synthesis and Electrochemical and Photophysical Properties. *Inorg. Chem.* **2004**, *43* (23), 7359–7368. https://doi.org/10.1021/ic0493043.
(176) Chen, J.-L.; Chi, Y.; Chen, K.; Cheng, Y.-M.; Chung, M.-W.; Yu, Y.-C.; Lee, G.-H.; Chou, P.-T.; Shu, C.-F. New Series of Ruthenium(II) and Osmium(II) Complexes Showing Solid-State Phosphorescence in Far-Visible and Near-Infrared. *Inorg. Chem.* **2010**, *49* (3), 823–832. https://doi.org/10.1021/ic900586e.
(177) Islam, A.; Ikeda, N.; Yoshimura, A.; Ohno, T. Nonradiative Transition of Phosphorescent Charge-Transfer States of Ruthenium(II)-to-2,2‘-Biquinoline and Ruthenium(II)-to-2,2‘:6‘,2‘‘-Terpyridine in the Solid State. *Inorg. Chem.* **1998**, *37* (12), 3093–3098. https://doi.org/10.1021/ic9702429.
(178) Demas, J. N.; Crosby, G. A. On the Multiplicity of the Emitting State of Ruthenium(II) Complexes. *Journal of Molecular Spectroscopy* **1968**, *26* (1), 72–77. https://doi.org/10.1016/0022-2852(68)90144-6.
(179) Beley, M.; Collin, J.-P.; Sauvage, J.-P.; Sugihara, H.; Heisel, F.; Miehé, A. Photophysical and Photochemical Properties of Ruthenium and Osmium Complexes with Substituted Terpyridines. *J. Chem. Soc., Dalton Trans.* **1991**, No. 11, 3157–3159. https://doi.org/10.1039/DT9910003157.
(180) Winkler, J. R.; Netzel, T. L.; Creutz, C.; Sutin, N. Direct Observation of Metal-to-Ligand Charge-Transfer (MLCT) Excited States of Pentaammineruthenium(II) Complexes. *J. Am. Chem. Soc.* **1987**, *109* (8), 2381–2392. https://doi.org/10.1021/ja00242a023.
(181) Constable, E. C.; Housecroft, C. E.; Schneider, G. E.; Zampese, J. A.; Bolink, H. J.; Pertegás, A.; Roldan-Carmona, C. Red Emitting [Ir(C^N)2(N^N)]+ Complexes Employing Bidentate 2,2′:6′,2″-Terpyridine Ligands for Light-Emitting Electrochemical Cells. *Dalton Trans.* **2014**, *43* (12), 4653–4667. https://doi.org/10.1039/C3DT53477D.
(182) Metcalfe, C.; Spey, S.; Adams, H.; Thomas, J. A. Extended Terpyridyl and Triazine Complexes of D6-Metal Centres. *J. Chem. Soc., Dalton Trans.* **2002**, No. 24, 4732–4739. https://doi.org/10.1039/B208211J.
(183) Figgemeier, E.; Merz, L.; Hermann, B. A.; Zimmermann, Y. C.; Housecroft, C. E.; Güntherodt, H.-J.; Constable, E. C. Self-Assembled Monolayers of Ruthenium and Osmium Bis-Terpyridine ComplexesInsights of the Structure and Interaction Energies by Combining Scanning Tunneling Microscopy and Electrochemistry. *J. Phys. Chem. B* **2003**, *107* (5), 1157–1162. https://doi.org/10.1021/jp026522d.
(184) Mulfort, K. L.; Tiede, D. M. Supramolecular Cobaloxime Assemblies for H2 Photocatalysis: An Initial Solution State Structure−Function Analysis. *J. Phys. Chem. B* **2010**, *114* (45), 14572–14581. https://doi.org/10.1021/jp1023636.
(185) Jiang, Q.; Yang, X.; Xiang, P.; Dudek, M.; Matczyszyn, K.; Samoc, M.; Tian, X.; Zhang, Q.; Luo, Y.; Wang, D.; Shi, P. Self-Assembled Heterometallic Complexes Showing Enhanced Two-Photon Absorption and Their Distribution in Living Cells. *New J. Chem.* **2021**, *45* (11), 4994–5001. https://doi.org/10.1039/D0NJ05219A.
(186) Arm, K. J.; Williams, J. A. G. A Cross-Coupling Strategy for the Synthesis of Dimetallic Assemblies Containing Mixed Bipyridine–Terpyridine Bridging Ligands: Luminescence and Energy Transfer Properties. *Dalton Trans.* **2006**, No. 18, 2172–2174. https://doi.org/10.1039/B602022D.

(187) Barthelmes, K.; Kübel, J.; Winter, A.; Wächtler, M.; Friebe, C.; Dietzek, B.; Schubert, U. S. New Ruthenium Bis(Terpyridine) Methanofullerene and Pyrrolidinofullerene Complexes: Synthesis and Electrochemical and Photophysical Properties. *Inorg. Chem.* **2015**, *54* (7), 3159–3171. https://doi.org/10.1021/ic502431x.
(188) Yao, C.-J.; Zheng, R.-H.; Nie, H.-J.; Cui, B.-B.; Shi, Q.; Yao, J.; Zhong, Y.-W. A Combined Experimental and Computational Study of Linear Ruthenium(II) Coordination Oligomers with End-Capping Organic Redox Sites: Insight into the Light Absorption and Charge Delocalization. *Chemistry – A European Journal* **2013**, *19* (37), 12376–12387. https://doi.org/10.1002/chem.201301319.
(189) Maity, D.; Das, S.; Mardanya, S.; Baitalik, S. Synthesis, Structural Characterization, and Photophysical, Spectroelectrochemical, and Anion-Sensing Studies of Heteroleptic Ruthenium(II) Complexes Derived from 4′-Polyaromatic-Substituted Terpyridine Derivatives and 2,6-Bis(Benzimidazol-2-Yl)Pyridine. *Inorg. Chem.* **2013**, *52* (12), 6820–6838. https://doi.org/10.1021/ic3022326.
(190) Motoyama, D.; Yoshikawa, K.; Ozawa, H.; Tadokoro, M.; Haga, M. Energy-Storage Applications for a pH Gradient between Two Benzimidazole-Ligated Ruthenium Complexes That Engage in Proton-Coupled Electron-Transfer Reactions in Solution. *Inorg. Chem.* **2017**, *56* (11), 6419–6428. https://doi.org/10.1021/acs.inorgchem.7b00518.
(191) Bhaumik, C.; Das, S.; Saha, D.; Dutta, S.; Baitalik, S. Synthesis, Characterization, Photophysical, and Anion-Binding Studies of Luminescent Heteroleptic Bis-Tridentate Ruthenium(II) Complexes Based on 2,6-Bis(Benzimidazole-2-Yl)Pyridine and 4′-Substituted 2,2′:6′,2″ Terpyridine Derivatives. *Inorg. Chem.* **2010**, *49* (11), 5049–5062. https://doi.org/10.1021/ic100138s.
(192) Singh, A.; Chetia, B.; Mobin, S. M.; Das, G.; Iyer, P. K.; Mondal, B. Ruthenium Monoterpyridine Complexes with 2,6-Bis(Benzimidazol-2-Yl)Pyridine: Synthesis, Spectral Properties and Structure. *Polyhedron* **2008**, *27* (8), 1983–1988. https://doi.org/10.1016/j.poly.2008.03.008.
(193) Mishra, D.; Barbieri, A.; Sabatini, C.; Drew, M. G. B.; Figgie, H. M.; Sheldrick, W. S.; Chattopadhyay, S. K. Tuning of Redox Potential and Visible Absorption Band of Ruthenium(II) Complexes of (Benzimidazolyl) Derivatives: Synthesis, Characterization, Spectroscopic and Redox Properties, X-Ray Structures and DFT Calculations. *Inorganica Chimica Acta* **2007**, *360* (7), 2231–2244. https://doi.org/10.1016/j.ica.2006.11.009.
(194) Meng, T.-T.; Wang, H.; Zheng, Z.-B.; Wang, K.-Z. pH-Switchable “Off–On–Off” Near-Infrared Luminescence Based on a Dinuclear Ruthenium(II) Complex. *Inorg. Chem.* **2017**, *56* (9), 4775–4779. https://doi.org/10.1021/acs.inorgchem.7b00223.
(195) Karges, J.; Blacque, O.; Jakubaszek, M.; Goud, B.; Goldner, P.; Gasser, G. Systematic Investigation of the Antiproliferative Activity of a Series of Ruthenium Terpyridine Complexes. *Journal of Inorganic Biochemistry* **2019**, *198*, 110752. https://doi.org/10.1016/j.jinorgbio.2019.110752.
(196) Amabilino, S.; Tasse, M.; Lacroix, P. G.; Mallet-Ladeira, S.; Pimienta, V.; Akl, J.; Sasaki, I.; Malfant, I. Photorelease of Nitric Oxide (NO) on Ruthenium Nitrosyl Complexes with Phenyl Substituted Terpyridines. *New J. Chem.* **2017**, *41* (15), 7371–7383. https://doi.org/10.1039/C7NJ00866J.
(197) Hirano, T.; Ueda, K.; Mukaida, M.; Nagao, H.; Oi, T. Reactions of [RuCl2(NO)(Terpy)]+ (Terpy = 2,2′ : 6′,2″-Terpyridine) with Mono Anions Such as NO2−, Br− and N3−, and Structural Studies on Terpyridineruthenium Having a Nitrosyl Ligand. *J. Chem. Soc., Dalton Trans.* **2001**, No. 16, 2341–2345. https://doi.org/10.1039/B101546J.
(198) Nagao, H.; Enomoto, K.; Wakabayashi, Y.; Komiya, G.; Hirano, T.; Oi, T. Synthesis of Nitrosylruthenium Complexes Containing 2,2‘:6‘,2‘ ‘-Terpyridine by Reactions of Alkoxo Complexes with Acids. *Inorg. Chem.* **2007**, *46* (4), 1431–1439. https://doi.org/10.1021/ic061644w.
(199) Karidi, K.; Garoufis, A.; Tsipis, A.; Hadjiliadis, N.; Dulk, H. den; Reedijk, J. Synthesis, Characterization, in Vitro Antitumor Activity, DNA-Binding Properties and Electronic Structure (DFT) of the New Complex Cis-(Cl,Cl)[RuIICl2(NO+)(Terpy)]Cl. *Dalton Trans.* **2005**, No. 7, 1176–1187. https://doi.org/10.1039/B418838A.
(200) Lin, Z.; Li, Z.; Xiao, W.; Kong, L.; Xu, J.; Xia, Y.; Zhu, X.; Zhang, F.; Ou, Y.-P. Terpyridine Ruthenium–Triarylamine Asymmetrical Mixed-Valence Systems: Syntheses, (Spectro) Electrochemistry and Theoretical Calculations. *Journal of Organometallic Chemistry* **2023**, *993*, 122708. https://doi.org/10.1016/j.jorganchem.2023.122708.
(201) Tan, B.-C.; Wang, Q.; Jiang, L.-L.; Song, Y.; Wu, X.-T.; Sheng, T.-L. Influence of Changing the Remote Substituents on Charge Transfer Properties of Cyanide-Bridged Trinuclear Fe−Ru−Fe Complexes. *European Journal of Inorganic Chemistry* **2023**, *26* (12), e202300003. https://doi.org/10.1002/ejic.202300003.
(202) Collin, J. P.; Guillerez, S.; Sauvage, J. P.; Barigelletti, F.; De Cola, L.; Flamigni, L.; Balzani, V. Photoinduced Processes in Dyads and Triads Containing a Ruthenium(II)-Bis(Terpyridine) Photosensitizer Covalently Linked to Electron Donor and Acceptor Groups. *Inorg. Chem.* **1991**, *30* (22), 4230–4238. https://doi.org/10.1021/ic00022a026.

(203) Bonhôte, P.; Moser, J.-E.; Humphry-Baker, R.; Vlachopoulos, N.; Zakeeruddin, S. M.; Walder, L.; Grätzel, M. Long-Lived Photoinduced Charge Separation and Redox-Type Photochromism on Mesoporous Oxide Films Sensitized by Molecular Dyads. *J. Am. Chem. Soc.* **1999**, *121* (6), 1324–1336. https://doi.org/10.1021/ja981742j.
(204) Colombo, A.; Locatelli, D.; Roberto, D.; Tessore, F.; Ugo, R.; Cavazzini, M.; Quici, S.; Angelis, F. D.; Fantacci, S.; Ledoux-Rak, I.; Tancrez, N.; Zyss, J. New [(D-Terpyridine)-Ru-(D or A-Terpyridine)][4-EtPhCO2]2 Complexes (D = Electron Donor Group; A = Electron Acceptor Group) as Active Second-Order Non Linear Optical Chromophores. *Dalton Trans.* **2012**, *41* (22), 6707–6714. https://doi.org/10.1039/C2DT30183K.
(205) Dumur, F.; Mayer, C. R.; Hoang-Thi, K.; Ledoux-Rak, I.; Miomandre, F.; Clavier, G.; Dumas, E.; Méallet-Renault, R.; Frigoli, M.; Zyss, J.; Sécheresse, F. Electrochemical, Linear Optical, and Nonlinear Optical Properties and Interpretation by Density Functional Theory Calculations of (4-N,N-Dimethylaminostyryl)-Pyridinium Pendant Group Associated with Polypyridinic Ligands and Respective Multifunctional Metal Complexes (RuII or ZnII). *Inorg. Chem.* **2009**, *48* (17), 8120–8133. https://doi.org/10.1021/ic900060d.
(206) Lainé, P.; Bedioui, F.; Ochsenbein, P.; Marvaud, V.; Bonin, M.; Amouyal, E. A New Class of Functionalized Terpyridyl Ligands as Building Blocks for Photosensitized Supramolecular Architectures. Synthesis, Structural, and Electronic Characterizations. *J. Am. Chem. Soc.* **2002**, *124* (7), 1364–1377. https://doi.org/10.1021/ja011069p.
(207) Konstantaki, M.; Koudoumas, E.; Couris, S.; Lainé, P.; Amouyal, E.; Leach, S. Substantial Non-Linear Optical Response of New Polyads Based on Ru and Os Complexes of Modified Terpyridines. *J. Phys. Chem. B* **2001**, *105* (44), 10797–10804. https://doi.org/10.1021/jp010497e.
(208) Bonhôte, P.; Moser, J. E.; Vlachopoulos, N.; Walder, L.; Zakeeruddin, S. M.; Humphry-Baker, R.; Péchy, P.; Grätzel, M. Photoinduced Electron Transfer and Redox-Type Photochromism of a TiO2-Anchored Molecular Diad. *Chem. Commun.* **1996**, No. 10, 1163–1164. https://doi.org/10.1039/CC9960001163.
(209) Qiu, D.; Zhao, Q.; Bao, X.; Liu, K.; Wang, H.; Guo, Y.; Zhang, L.; Zeng, J.; Wang, H. Electropolymerization and Characterization of an Alternatively Conjugated Donor–Acceptor Metallopolymer: Poly-[Ru(4′-(4-(Diphenylamino)Phenyl)-2,2′:6′,2″-Terpyridine)2]2+. *Inorganic Chemistry Communications* **2011**, *14* (1), 296–299. https://doi.org/10.1016/j.inoche.2010.11.019.
(210) Rosero, W. A. A.; Guimaraes, R. R.; Matias, T. A.; Araki, K. Effect of Push-Pull Ruthenium Complex Adsorption Conformation on the Performance of Dye Sensitized Solar Cells. *J. Braz. Chem. Soc.* **2020**, *31*, 2250–2264. https://doi.org/10.21577/0103-5053.20200077.
(211) Rosenthal, M.; Lindner, J. K. N.; Gerstmann, U.; Meier, A.; Schmidt, W. G.; Wilhelm, R. A Photoredox Catalysed Heck Reaction via Hole Transfer from a Ru(II)-Bis(Terpyridine) Complex to Graphene Oxide. *RSC Adv.* **2020**, *10* (70), 42930–42937. https://doi.org/10.1039/D0RA08749A.
(212) Liang, X.; Qian, S.; Lou, Z.; Hu, R.; Hou, Y.; Chen, P. R.; Fan, X. Near Infrared Light-Triggered Photocatalytic Decaging for Remote-Controlled Spatiotemporal Activation in Living Mice. *Angewandte Chemie International Edition* **2023**, *62* (48), e202310920. https://doi.org/10.1002/anie.202310920.
(213) Cerfontaine, S.; Duez, Q.; Troian-Gautier, L.; Barozzino-Consiglio, G.; Loiseau, F.; Cornil, J.; De Winter, J.; Gerbaux, P.; Elias, B. Efficient Convergent Energy Transfer in a Stereoisomerically Pure Heptanuclear Luminescent Terpyridine-Based Ru(II)–Os(II) Dendrimer. *Inorg. Chem.* **2020**, *59* (19), 14536–14543. https://doi.org/10.1021/acs.inorgchem.0c02336.
(214) Chichak, K.; Branda, N. R. Self-Assembly of a Linear Multicomponent Porphyrin Array through Axial Coordination. *Chem. Commun.* **1999**, No. 6, 523–524. https://doi.org/10.1039/A901231A.
(215) Miyake, Y.; Nakajima, K.; Sasaki, K.; Saito, R.; Nakanishi, H.; Nishibayashi, Y. Design and Synthesis of Diphosphine Ligands Bearing an Osmium(II) Bis(Terpyridyl) Moiety as a Light-Harvesting Unit: Application to Photocatalytic Production of Dihydrogen. *Organometallics* **2009**, *28* (17), 5240–5243. https://doi.org/10.1021/om900489d.
(216) Kober, E. M.; Marshall, J. L.; Dressick, W. J.; Sullivan, B. P.; Caspar, J. V.; Meyer, T. J. Synthetic Control of Excited States. Nonchromophoric Ligand Variations in Polypyridyl Complexes of Osmium(II). *Inorg. Chem.* **1985**, *24* (18), 2755–2763. https://doi.org/10.1021/ic00212a010.
(217) Haruki, R.; Sasaki, Y.; Masutani, K.; Yanai, N.; Kimizuka, N. Leaping across the Visible Range: Near-Infrared-to-Violet Photon Upconversion Employing a Silyl-Substituted Anthracene. *Chem. Commun.* **2020**, *56* (51), 7017–7020. https://doi.org/10.1039/D0CC02240C.
(218) Lacky, D. E.; Pankuch, B. J.; Crosby, G. A. Charge-Transfer Excited States of Osmium(II) Complexes. 2. Quantum-Yield and Decay-Time Measurements. *J. Phys. Chem.* **1980**, *84* (16), 2068–2074. https://doi.org/10.1021/j100453a014.
(219) Liu, D. K.; Brunschwig, B. S.; Creutz, Carol.; Sutin, Norman. Formation of Electronically Excited Products in Electron-Transfer Reactions: Reaction of Polypyridine Complexes of Cobalt(I) and

Ruthenium(III) in Acetonitrile. *J. Am. Chem. Soc.* **1986**, *108* (8), 1749–1755. https://doi.org/10.1021/ja00268a006.
(220) Lainé, P.; Amouyal, E. Photophysical Properties of Osmium(II) Complexes with the Novel 4′-p-Phenylterpyridine-Triarylpyridinium Ligand. *Chem. Commun.* **1999**, No. 10, 935–936. https://doi.org/10.1039/A901237K.
(221) Barbour, J. C.; Kim, A. J. I.; deVries, E.; Shaner, S. E.; Lovaasen, B. M. Chromium(III) Bis-Arylterpyridyl Complexes with Enhanced Visible Absorption via Incorporation of Intraligand Charge-Transfer Transitions. *Inorg. Chem.* **2017**, *56* (14), 8212–8222. https://doi.org/10.1021/acs.inorgchem.7b00953.
(222) Constable, E. C.; Housecroft, C. E.; Neuburger, M.; Schönle, J.; Zampese, J. A. The Surprising Lability of Bis(2,2′:6′,2″-Terpyridine)Chromium(III) Complexes. *Dalton Trans.* **2014**, *43* (19), 7227–7235. https://doi.org/10.1039/C4DT00200H.
(223) Jiménez, J.-R.; Doistau, B.; Besnard, C.; Piguet, C. Versatile Heteroleptic Bis-Terdentate Cr(III) Chromophores Displaying Room Temperature Millisecond Excited State Lifetimes. *Chem. Commun.* **2018**, *54* (94), 13228–13231. https://doi.org/10.1039/C8CC07671E.
(224) Ohno, T.; Kato, S.; Kaizaki, S.; Hanazaki, I. Singlet-Triplet Transitions of Aromatic Compounds Coordinating to a Paramagnetic Chromium(III) Ion. *Inorg. Chem.* **1986**, *25* (22), 3853–3858. https://doi.org/10.1021/ic00242a005.
(225) Vaidyanathan, V. G.; Nair, B. U. Nucleobase Oxidation of DNA by (Terpyridyl)Chromium(III) Derivatives. *European Journal of Inorganic Chemistry* **2004**, *2004* (9), 1840–1846. https://doi.org/10.1002/ejic.200300718.
(226) Hughes, M. C.; Macero, D. J. Electrochemical Evidence for Reversible Five-Membered Electron Transfer Chains in Octahedral Chromium Complexes. *Inorg. Chem.* **1976**, *15* (9), 2040–2044. https://doi.org/10.1021/ic50163a005.
(227) Rao, J. M.; Hughes, M. C.; Macero, D. J. Redox Behavior of Aromatic Tridentate Imine Ligand Complexes of Manganese and Chromium. *Inorganica Chimica Acta* **1976**, *18*, 127–131. https://doi.org/10.1016/S0020-1693(00)95591-9.
(228) Schönle, J.; Constable, E. C.; Housecroft, C. E.; Prescimone, A.; Zampese, J. A. Homoleptic and Heteroleptic Complexes of Chromium(III) Containing 4′-Diphenylamino-2,2′:6′,2″-Terpyridine Ligands. *Polyhedron* **2015**, *89*, 182–188. https://doi.org/10.1016/j.poly.2015.01.015.
(229) Farran, R.; Le-Quang, L.; Mouesca, J.-M.; Maurel, V.; Jouvenot, D.; Loiseau, F.; Deronzier, A.; Chauvin, J. [Cr(Ttpy)2]3+ as a Multi-Electron Reservoir for Photoinduced Charge Accumulation. *Dalton Trans.* **2019**, *48* (20), 6800–6811. https://doi.org/10.1039/C9DT00848A.
(230) Li, Z.-Q.; Gong, Z.-L.; Liang, T.; Bernhard, S.; Zhong, Y.-W.; Yao, J. Circularly Polarized Phosphorescence and Photon Transport of Micro/Nanocrystals of Ruthenium and Iridium Complexes with Chiral Anions. *Sci. China Chem.* **2023**, *66* (10), 2892–2902. https://doi.org/10.1007/s11426-023-1723-7.
(231) Palion-Gazda, J.; Kwiecień, A.; Choroba, K.; Penkala, M.; Kryczka, A.; Machura, B. The Role of Intraligand Charge Transfer Processes in Iridium(III) Complexes with Morpholine-Decorated 4′-Phenyl-2,2′:6′,2″-Terpyridine. *Molecules* **2024**, *29* (13), 3074. https://doi.org/10.3390/molecules29133074.
(232) Wei, L.; Kushwaha, R.; Dao, A.; Fan, Z.; Banerjee, S.; Huang, H. Axisymmetric Bis-Tridentate Ir(III) Photoredox Catalysts for Anticancer Phototherapy under Hypoxia. *Chem. Commun.* **2023**, *59* (21), 3083–3086. https://doi.org/10.1039/D2CC06721H.
(233) Ito, W.; Hattori, S.; Kondo, M.; Sakagami, H.; Kobayashi, O.; Ishimoto, T.; Shinozaki, K. Dual Emission from an Iridium(III) Complex/Counter Anion Ion Pair. *Dalton Trans.* **2021**, *50* (5), 1887–1894. https://doi.org/10.1039/D1DT00021G.
(234) Titos-Padilla, S.; Colacio, E.; Pope, S. J. A.; Delgado, J. J.; Melgosa, M.; Herrera, J. M. Photophysical Properties of [Ir(Tpy)2]3+-Doped Silica Nanoparticles and Synthesis of a Colour-Tunable Material Based on an Ir(Core)–Eu(Shell) Derivative. *J. Mater. Chem. C* **2013**, *1* (24), 3808–3815. https://doi.org/10.1039/C3TC30466C.
(235) Yoshikawa, N.; Yamabe, S.; Kanehisa, N.; Kai, Y.; Takashima, H.; Tsukahara, K. Structures of Polypyridine Mononuclear IrIII Complexes in the Ground State and the Lowest Triplet State. *Inorganica Chimica Acta* **2009**, *362* (2), 361–371. https://doi.org/10.1016/j.ica.2008.04.021.
(236) Yoshikawa, N.; Yamabe, S.; Kanehisa, N.; Kai, Y.; Takashima, H.; Tsukahara, K. Synthesis, Characterization, and DFT Investigation of IrIII Tolylterpyridine Complexes. *European Journal of Inorganic Chemistry* **2007**, *2007* (13), 1911–1919. https://doi.org/10.1002/ejic.200600995.
(237) Ayala, N. P.; Flynn, C. M. Jr.; Sacksteder, L.; Demas, J. N.; DeGraff, B. A. Synthesis, Luminescence, and Excited-State Complexes of the Tris(1,10-Phenanthroline)- and Bis(Terpyridine)Iridium(III) Cations. *J. Am. Chem. Soc.* **1990**, *112* (10), 3837–3844. https://doi.org/10.1021/ja00166a018.

(238) Hattori, S.; Kondo, M.; Sekine, A.; Shinozaki, K. Vapochromism of an Iridium(III) Bis-Terpyridine Complex Based on the Modulation of Halide-to-Ligand Charge Transfer Transition. *Dalton Trans.* **2022**, *51* (18), 7068–7075. https://doi.org/10.1039/D2DT00368F.
(239) Yoshikawa, N.; Yamabe, S.; Kanehisa, N.; Inoue, T.; Takashima, H.; Tsukahara, K. Detailed Description of the Metal-to-Ligand Charge-Transfer State in Monoterpyridine IrIII Complexes. *European Journal of Inorganic Chemistry* **2009**, *2009* (14), 2067–2073. https://doi.org/10.1002/ejic.200801139.
(240) Liu, B.; Jabed, M. A.; Kilina, S.; Sun, W. Synthesis, Photophysics, and Reverse Saturable Absorption of Trans-Bis-Cyclometalated Iridium(III) Complexes (C^N^C)Ir(R-Tpy)+ (Tpy = 2,2′:6′,2″-Terpyridine) with Broadband Excited-State Absorption. *Inorg. Chem.* **2020**, *59* (12), 8532–8542. https://doi.org/10.1021/acs.inorgchem.0c00961.
(241) Chirdon, D. N.; Transue, W. J.; Kagalwala, H. N.; Kaur, A.; Maurer, A. B.; Pintauer, T.; Bernhard, S. [Ir(N^N^N)(C^N)L]+: A New Family of Luminophores Combining Tunability and Enhanced Photostability. *Inorg. Chem.* **2014**, *53* (3), 1487–1499. https://doi.org/10.1021/ic402411g.
(242) Palion-Gazda, J.; Kwiecień, A.; Choroba, K.; Penkala, M.; Erfurt, K.; Machura, B. Effect of the Appended Morpholinyl Group on Photophysical Behavior of Mono- and Bis-Cyclometalated Terpyridine Iridium(III) Chromophores. *Inorg. Chem.* **2025**, *64* (1), 646–661. https://doi.org/10.1021/acs.inorgchem.4c03769.
(243) Genoni, A.; Chirdon, D. N.; Boniolo, M.; Sartorel, A.; Bernhard, S.; Bonchio, M. Tuning Iridium Photocatalysts and Light Irradiation for Enhanced CO2 Reduction. *ACS Catal.* **2017**, *7* (1), 154–160. https://doi.org/10.1021/acscatal.6b03227.
(244) Kuang, S.; Sun, L.; Zhang, X.; Liao, X.; Rees, T. W.; Zeng, L.; Chen, Y.; Zhang, X.; Ji, L.; Chao, H. A Mitochondrion-Localized Two-Photon Photosensitizer Generating Carbon Radicals Against Hypoxic Tumors. *Angewandte Chemie International Edition* **2020**, *59* (46), 20697–20703. https://doi.org/10.1002/anie.202009888.
(245) Müller, V. V. L.; Moreth, D.; Kowalski, K.; Kowalczyk, A.; Gapińska, M.; Kutta, R. J.; Nuernberger, P.; Schatzschneider, U. Tuning The Intracellular Distribution of [3+2+1] Iridium(III) Complexes In Bacterial And Mammalian Cells By iClick Reaction With Biomolecular Carriers Functionalized With Alkynone Groups. *Chemistry – A European Journal* **2024**, *30* (56), e202401603. https://doi.org/10.1002/chem.202401603.
(246) Fan, Z.; Rong, Y.; Sadhukhan, T.; Liang, S.; Li, W.; Yuan, Z.; Zhu, Z.; Guo, S.; Ji, S.; Wang, J.; Kushwaha, R.; Banerjee, S.; Raghavachari, K.; Huang, H. Single-Cell Quantification of a Highly Biocompatible Dinuclear Iridium(III) Complex for Photocatalytic Cancer Therapy. *Angewandte Chemie International Edition* **2022**, *61* (23), e202202098. https://doi.org/10.1002/anie.202202098.
(247) Takizawa, S.; Katoh, S.; Okazawa, A.; Ikuta, N.; Matsushima, S.; Zeng, F.; Murata, S. Triplet Excited States Modulated by Push–Pull Substituents in Monocyclometalated Iridium(III) Photosensitizers. *Inorg. Chem.* **2021**, *60* (7), 4891–4903. https://doi.org/10.1021/acs.inorgchem.0c03802.
(248) Sato, S.; Morikawa, T.; Kajino, T.; Ishitani, O. A Highly Efficient Mononuclear Iridium Complex Photocatalyst for CO2 Reduction under Visible Light. *Angewandte Chemie International Edition* **2013**, *52* (3), 988–992. https://doi.org/10.1002/anie.201206137.
(249) Zhang, Q.; Lu, X.; Wang, H.; Tian, X.; Wang, A.; Zhou, H.; Wu, J.; Tian, Y. A Benzoic Acid Terpyridine-Based Cyclometalated Iridium(III) Complex as a Two-Photon Fluorescence Probe for Imaging Nuclear Histidine. *Chem. Commun.* **2018**, *54* (30), 3771–3774. https://doi.org/10.1039/C8CC00908B.
(250) Leslie, W.; Poole, R. A.; Murray, P. R.; Yellowlees, L. J.; Beeby, A.; Williams, J. A. G. Near Infra-Red Luminescence from Bis-Terpyridyl Iridium(III) Complexes Incorporating Electron-Rich Pendants. *Polyhedron* **2004**, *23* (17), 2769–2777. https://doi.org/10.1016/j.poly.2004.08.009.
(251) Goldstein, D. C.; Cheng, Y. Y.; Schmidt, T. W.; Bhadbhade, M.; Thordarson, P. Photophysical Properties of a New Series of Water Soluble Iridium Bisterpyridine Complexes Functionalised at the 4′ Position. *Dalton Trans.* **2011**, *40* (9), 2053–2061. https://doi.org/10.1039/C0DT01317J.
(252) Baranoff, E.; Dixon, I. M.; Collin, J.-P.; Sauvage, J.-P.; Ventura, B.; Flamigni, L. Dyads Containing Iridium(III) Bis-Terpyridine as Photoactive Center: Synthesis and Electron Transfer Study. *Inorg. Chem.* **2004**, *43* (10), 3057–3066. https://doi.org/10.1021/ic0351038.
(253) Kumar Yadav, A.; Singh, V.; Acharjee, S.; Saha, S.; Kushwaha, R.; Dutta, A.; Koch, B.; Banerjee, S. Sonodynamic Cancer Therapy by Mn(I)-Tricarbonyl Complexes via Ultrasound-Triggered CO Release and ROS Generation. *Chemistry – A European Journal* **2025**, *31* (3), e202403454. https://doi.org/10.1002/chem.202403454.
(254) Compain, J.-D.; Bourrez, M.; Haukka, M.; Deronzier, A.; Chardon-Noblat, S. Manganese Carbonyl Terpyridyl Complexes: Their Synthesis, Characterization and Potential Application as CO-Release Molecules. *Chem. Commun.* **2014**, *50* (19), 2539–2542. https://doi.org/10.1039/C4CC00197D.
(255) Machan, C. W.; Kubiak, C. P. Electrocatalytic Reduction of Carbon Dioxide with Mn(Terpyridine) Carbonyl Complexes. *Dalton Trans.* **2016**, *45* (43), 17179–17186. https://doi.org/10.1039/C6DT03243E.

(256) Palion-Gazda, J.; Szłapa-Kula, A.; Penkala, M.; Erfurt, K.; Machura, B. Photoinduced Processes in Rhenium(I) Terpyridine Complexes Bearing Remote Amine Groups: New Insights from Transient Absorption Spectroscopy. *Molecules* **2022**, *27* (21), 7147. https://doi.org/10.3390/molecules27217147.
(257) Fernández-Terán, R.; Sévery, L. Living Long and Prosperous: Productive Intraligand Charge-Transfer States from a Rhenium(I) Terpyridine Photosensitizer with Enhanced Light Absorption. *Inorg. Chem.* **2021**, *60* (3), 1334–1343. https://doi.org/10.1021/acs.inorgchem.0c01939.
(258) Kushwaha, R.; Upadhyay, A.; Saha, S.; Yadav, A. K.; Bera, A.; Dutta, A.; Banerjee, S. Cancer Phototherapy by CO Releasing Terpyridine-Based Re(I) Tricarbonyl Complexes via ROS Generation and NADH Oxidation. *Dalton Trans.* **2024**, *53* (32), 13591–13601. https://doi.org/10.1039/D4DT01309C.
(259) Juris, A.; Campagna, S.; Bidd, I.; Lehn, J. M.; Ziessel, R. Synthesis and Photophysical and Electrochemical Properties of New Halotricarbonyl(Polypyridine)Rhenium(I) Complexes. *Inorg. Chem.* **1988**, *27* (22), 4007–4011. https://doi.org/10.1021/ic00295a022.
(260) Klein, A.; Vogler, C.; Kaim, W. The δ in 18 + δ Electron Complexes: Importance of the Metal/Ligand Interface for the Substitutional Reactivity of "Re(0)" Complexes (α-Diimine-)ReI(CO)3(X). *Organometallics* **1996**, *15* (1), 236–244. https://doi.org/10.1021/om950500e.
(261) Amoroso, A. J.; Banu, A.; Coogan, M. P.; Edwards, P. G.; Hossain, G.; Malik, K. M. A. Functionalisation of Terpyridine Complexes Containing the Re(CO)3+ Moiety. *Dalton Trans.* **2010**, *39* (30), 6993–7003. https://doi.org/10.1039/C0DT00174K.
(262) Bulsink, P.; Al-Ghamdi, A.; Joshi, P.; Korobkov, I.; Woo, T.; Richeson, D. Capturing Re(I) in an Neutral N,N,N Pincer Scaffold and Resulting Enhanced Absorption of Visible Light. *Dalton Trans.* **2016**, *45* (21), 8885–8896. https://doi.org/10.1039/C6DT00661B.
(263) Kushwaha, R.; Upadhyay, A.; Peters, S.; Yadav, A. K.; Mishra, A.; Bera, A.; Sadhukhan, T.; Banerjee, S. Visible and Red Light-Triggered Anticancer Profile of a Ferrocene-Re(I)-Tricarbonyl Conjugate: Experimental and Theoretical Studies. *Langmuir* **2024**, *40* (23), 12226–12238. https://doi.org/10.1021/acs.langmuir.4c01296.
(264) Auvray, T.; Del Secco, B.; Dubreuil, A.; Zaccheroni, N.; Hanan, G. S. In-Depth Study of the Electronic Properties of NIR-Emissive κ3N Terpyridine Rhenium(I) Dicarbonyl Complexes. *Inorg. Chem.* **2021**, *60* (1), 70–79. https://doi.org/10.1021/acs.inorgchem.0c02188.
(265) Fernández-Terán, R. J.; Sévery, L. Coordination Environment Prevents Access to Intraligand Charge-Transfer States through Remote Substitution in Rhenium(I) Terpyridinedicarbonyl Complexes. *Inorg. Chem.* **2021**, *60* (3), 1325–1333. https://doi.org/10.1021/acs.inorgchem.0c02914.
(266) Black, D. R.; Hightower, S. E. Preparation and Characterization of Rhenium(I) Dicarbonyl Complexes Based on the Meridionally-Coordinated Terpyridine Ligand. *Inorganic Chemistry Communications* **2012**, *24*, 16–19. https://doi.org/10.1016/j.inoche.2012.07.034.
(267) Frenzel, B. A.; Schumaker, J. E.; Black, D. R.; Hightower, S. E. Synthesis, Spectroscopic, Electrochemical and Computational Studies of Rhenium(I) Dicarbonyl Complexes Based on Meridionally-Coordinated 2,2′:6′,2″-Terpyridine. *Dalton Trans.* **2013**, *42* (34), 12440–12451. https://doi.org/10.1039/C3DT51251G.
(268) Klemens, T.; Świtlicka-Olszewska, A.; Machura, B.; Grucela, M.; Janeczek, H.; Schab-Balcerzak, E.; Szlapa, A.; Kula, S.; Krompiec, S.; Smolarek, K.; Kowalska, D.; Mackowski, S.; Erfurt, K.; Lodowski, P. Synthesis, Photophysical Properties and Application in Organic Light Emitting Devices of Rhenium(I) Carbonyls Incorporating Functionalized 2,2′:6′,2″-Terpyridines. *RSC Adv.* **2016**, *6* (61), 56335–56352. https://doi.org/10.1039/C6RA08981J.
(269) Klemens, T.; Świtlicka, A.; Szlapa-Kula, A.; Łapok, Ł.; Obłoza, M.; Siwy, M.; Szalkowski, M.; Maćkowski, S.; Libera, M.; Schab-Balcerzak, E.; Machura, B. Tuning Optical Properties of Re(I) Carbonyl Complexes by Modifying Push–Pull Ligands Structure. *Organometallics* **2019**, *38* (21), 4206–4223. https://doi.org/10.1021/acs.organomet.9b00517.
(270) Mansour, A. M.; Radacki, K. Terpyridine Based ReX(CO)3 Compounds (X = Br–, N3– and Triazolate): Spectroscopic and DFT Studies. *Polyhedron* **2021**, *194*, 114954. https://doi.org/10.1016/j.poly.2020.114954.
(271) Michalec, J. F.; Bejune, S. A.; McMillin, D. R. Multiple Ligand-Based Emissions from a Platinum(II) Terpyridine Complex Attached to Pyrene. *Inorg. Chem.* **2000**, *39* (13), 2708–2709. https://doi.org/10.1021/ic000304m.
(272) Mitra, K.; Basu, U.; Khan, I.; Maity, B.; Kondaiah, P.; Chakravarty, A. R. Remarkable Anticancer Activity of Ferrocenyl-Terpyridine Platinum(II) Complexes in Visible Light with Low Dark Toxicity. *Dalton Trans.* **2013**, *43* (2), 751–763. https://doi.org/10.1039/C3DT51922H.
(273) Ramu, V.; Gautam, S.; Garai, A.; Kondaiah, P.; Chakravarty, A. R. Glucose-Appended Platinum(II)-BODIPY Conjugates for Targeted Photodynamic Therapy in Red Light. *Inorg. Chem.* **2018**, *57* (4), 1717–1726. https://doi.org/10.1021/acs.inorgchem.7b02249.

(274) Tong, K.-C.; Wan, P.-K.; Lok, C.-N.; Che, C.-M. Dynamic Supramolecular Self-Assembly of Platinum(II) Complexes Perturbs an Autophagy–Lysosomal System and Triggers Cancer Cell Death. *Chem. Sci.* **2021**, *12* (46), 15229–15238. https://doi.org/10.1039/D1SC02841C.
(275) Shingade, V. M.; Grove, L. J.; Connick, W. B. Luminescent Pt(2,6-Bis(N-Methylbenzimidazol-2-Yl)Pyridine)X+: A Comparison with the Spectroscopic and Electrochemical Properties of Pt(Tpy)X+ (X = Cl, CCPh, Ph, or CH3). *Dalton Trans.* **2020**, *49* (28), 9651–9661. https://doi.org/10.1039/D0DT01496F.
(276) Morel, E.; Beauvineau, C.; Naud-Martin, D.; Landras-Guetta, C.; Verga, D.; Ghosh, D.; Achelle, S.; Mahuteau-Betzer, F.; Bombard, S.; Teulade-Fichou, M.-P. Selectivity of Terpyridine Platinum Anticancer Drugs for G-Quadruplex DNA. *Molecules* **2019**, *24* (3), 404. https://doi.org/10.3390/molecules24030404.
(277) Yin, L.; Zhang, J.; Yao, J.; Li, H. A Designed TEMPO-Derivate Catalyst with Switchable Signals of EPR and Photoluminescence: Application in the Mechanism of Alcohol Oxidation. *ChemCatChem* **2018**, *10* (16), 3513–3519. https://doi.org/10.1002/cctc.201800345.
(278) Hung, F.-F.; Wu, S.-X.; To, W.-P.; Kwong, W.-L.; Guan, X.; Lu, W.; Low, K.-H.; Che, C.-M. Palladium(II) Acetylide Complexes with Pincer-Type Ligands: Photophysical Properties, Intermolecular Interactions, and Photo-Cytotoxicity. *Chemistry – An Asian Journal* **2017**, *12* (1), 145–158. https://doi.org/10.1002/asia.201601414.
(279) Phillips, V.; Baddour, F. G.; Lasanta, T.; López-de-Luzuriaga, J. M.; Bacon, J. W.; Golen, J. A.; Rheingold, A. L.; Doerrer, L. H. Metal–Metal Stacking Patterns between and with [Pt(Tpy)X]+ Cations. *Inorganica Chimica Acta* **2010**, *364* (1), 195–204. https://doi.org/10.1016/j.ica.2010.07.038.
(280) Göransson, E.; Boixel, J.; Monnereau, C.; Blart, E.; Pellegrin, Y.; Becker, H.-C.; Hammarström, L.; Odobel, F. Photoinduced Electron Transfer in Zn(II)porphyrin−Bridge−Pt(II)Acetylide Complexes: Variation in Rate with Anchoring Group and Position of the Bridge. *Inorg. Chem.* **2010**, *49* (21), 9823–9832. https://doi.org/10.1021/ic100605t.
(281) Yuen, M.-Y.; Roy, V. a. L.; Lu, W.; Kui, S. C. F.; Tong, G. S. M.; So, M.-H.; Chui, S. S.-Y.; Muccini, M.; Ning, J. Q.; Xu, S. J.; Che, C.-M. Semiconducting and Electroluminescent Nanowires Self-Assembled from Organoplatinum(II) Complexes. *Angewandte Chemie International Edition* **2008**, *47* (51), 9895–9899. https://doi.org/10.1002/anie.200802981.
(282) Yam, V. W.-W.; Tang, R. P.-L.; Wong, K. M.-C.; Cheung, K.-K. Synthesis, Luminescence, Electrochemistry, and Ion-Binding Studies of Platinum(II) Terpyridyl Acetylide Complexes. *Organometallics* **2001**, *20* (22), 4476–4482. https://doi.org/10.1021/om010336x.
(283) Moriuchi, T.; Yamada, M.; Yoshii, K.; Hirao, T. Controlled Emission of Platinum(II) Terpyridyl Complexes with Poly-l-Glutamic Acid. *Journal of Organometallic Chemistry* **2010**, *695* (23), 2562–2566. https://doi.org/10.1016/j.jorganchem.2010.07.024.
(284) Yu, C.; Wong, K. M.-C.; Chan, K. H.-Y.; Yam, V. W.-W. Polymer-Induced Self-Assembly of Alkynylplatinum(II) Terpyridyl Complexes by Metal···Metal/Π···π Interactions. *Angewandte Chemie International Edition* **2005**, *44* (5), 791–794. https://doi.org/10.1002/anie.200461261.
(285) Sánchez-Cano, C.; Hannon, M. J. Cytotoxicity, Cellular Localisation and Biomolecular Interaction of Non-Covalent Metallo-Intercalators with Appended Sex Hormone Steroid Vectors. *Dalton Trans.* **2009**, No. 48, 10765–10773. https://doi.org/10.1039/B912711A.
(286) Du, P.; Schneider, J.; Jarosz, P.; Zhang, J.; Brennessel, W. W.; Eisenberg, R. Photoinduced Electron Transfer in Platinum(II) Terpyridyl Acetylide Chromophores: Reductive and Oxidative Quenching and Hydrogen Production. *J. Phys. Chem. B* **2007**, *111* (24), 6887–6894. https://doi.org/10.1021/jp072187n.
(287) Yang, Q.-Z.; Wu, L.-Z.; Wu, Z.-X.; Zhang, L.-P.; Tung, C.-H. Long-Lived Emission from Platinum(II) Terpyridyl Acetylide Complexes. *Inorg. Chem.* **2002**, *41* (22), 5653–5655. https://doi.org/10.1021/ic025580a.
(288) Yang, Q.-Z.; Tong, Q.-X.; Wu, L.-Z.; Wu, Z.-X.; Zhang, L.-P.; Tung, C.-H. Switch of the Lowest Excited-States of Terpyridylplatinum(II) Acetylide Complexes Bearing Amino or Azacrown Moieties by Proton and Cations. *European Journal of Inorganic Chemistry* **2004**, *2004* (9), 1948–1954. https://doi.org/10.1002/ejic.200300878.
(289) Büchner, R.; Field, J. S.; Haines, R. J.; Ledwaba, L. P.; McGuire, R.; McMillin, D. R.; Munro, O. Q. Synthesis, Crystal Structure and Solid State Photoluminescence of [Pt(Trpy)(CCPh)]SbF6 (Trpy=2,2′:6′,2″-Terpyridine). *Inorganica Chimica Acta* **2007**, *360* (5), 1633–1638. https://doi.org/10.1016/j.ica.2006.08.061.
(290) Maroń, A. M.; Choroba, K.; Małecki, J. G.; Kula, S.; Malicka, E. Platinum(II) Coordination Compound with 4′-[4-(Dimethylamino)Phenyl]-2,2′:6′,2″-Terpyridine – The New Insight into the Luminescence Behavior and Substituent Effect. *Polyhedron* **2020**, *182*, 114502. https://doi.org/10.1016/j.poly.2020.114502.
(291) Shi, P.; Jiang, Q.; Zhang, Q.; Tian, Y. Synthesis, Characterization, Emission and DNA Binding Properties of Four Alkynylplatinum(II) Terpyridine Complexes. *Journal of Organometallic Chemistry* **2016**, *804*, 66–72. https://doi.org/10.1016/j.jorganchem.2015.12.017.
(292) Gu, H.; Sun, X.; Zhao, Q.; Wang, H.; Cheng, X.; Yang, C.; Qiu, D. Near-IR Electrochromic Film with High Optical Contrast and Stability Prepared by Oxidative Electropolymerization of Triphenylamine

Modified Terpyridine Platinum(II) Chloride. *Molecules* **2023**, *28* (24), 8027. https://doi.org/10.3390/molecules28248027.

(293) Zou, H.-H.; Wang, L.; Long, Z.-X.; Qin, Q.-P.; Song, Z.-K.; Xie, T.; Zhang, S.-H.; Liu, Y.-C.; Lin, B.; Chen, Z.-F. Preparation of 4-([2,2′:6′,2″-Terpyridin]-4′-Yl)-N,N-Diethylaniline NiII and PtII Complexes and Exploration of Their *in Vitro* Cytotoxic Activities. *European Journal of Medicinal Chemistry* **2016**, *108*, 1–12. https://doi.org/10.1016/j.ejmech.2015.11.005.
